# Design and Optimization of a *Monkeypox virus* Specific Serological Assay

**DOI:** 10.3390/pathogens12030396

**Published:** 2023-03-01

**Authors:** Taha Y. Taha, Michael B. Townsend, Jan Pohl, Kevin L. Karem, Inger K. Damon, Placide Mbala Kingebeni, Jean-Jacques Muyembe Tamfum, James W. Martin, Phillip R. Pittman, John W. Huggins, Panayampalli S. Satheshkumar, Dennis A. Bagarozzi, Mary G. Reynolds, Laura J. Hughes

**Affiliations:** 1 Reagent and Diagnostic Services Branch, Division of Scientific Resources, National Center for Emerging and Zoonotic Infectious Diseases, Centers for Disease Control and Prevention, Atlanta, GA 30333, USA; 2 Poxvirus and Rabies Branch, Division of High Consequence Pathogens and Pathology, National Center for Emerging and Zoonotic Infectious Diseases, Centers for Disease Control and Prevention, Atlanta, GA 30333, USA; 3 Biotechnology Core Facility Branch, Division of Scientific Resources, National Center for Emerging and Zoonotic Infectious Diseases, Centers for Disease Control and Prevention, Atlanta, GA 30333, USA; 4 Institut National de Recherche Biomédicale, Ministère de la Santé Publique, Kinshasa P.O. Box 1197, Democratic Republic of the Congo; 5 Department of Clinical Research, Division of Medicine, U.S. Army Medical Research Institute of Infectious Diseases (USAMRIID), Fort Detrick, MD 21702, USA

**Keywords:** Mpox, ELISA, epidemiology, species specific immune response

## Abstract

Monkeypox virus (MPXV), a member of the *Orthopoxvirus* (OPXV) genus, is a zoonotic virus, endemic to central and western Africa that can cause smallpox-like symptoms in humans with fatal outcomes in up to 15% of patients. The incidence of MPXV infections in the Democratic Republic of the Congo, where the majority of cases have occurred historically, has been estimated to have increased as much as 20-fold since the end of smallpox vaccination in 1980. Considering the risk global travel carries for future disease outbreaks, accurate epidemiological surveillance of MPXV is warranted as demonstrated by the recent Mpox outbreak, where the majority of cases were occurring in non-endemic areas. Serological differentiation between childhood vaccination and recent infection with MPXV or other OPXVs is difficult due to the high level of conservation within OPXV proteins. Here, a peptide-based serological assay was developed to specifically detect exposure to MPXV. A comparative analysis of immunogenic proteins across human OPXVs identified a large subset of proteins that could potentially be specifically recognized in response to a MPXV infection. Peptides were chosen based upon MPXV sequence specificity and predicted immunogenicity. Peptides individually and combined were screened in an ELISA against serum from well-characterized Mpox outbreaks, vaccinee sera, and smallpox sera collected prior to eradication. One peptide combination was successful with ~86% sensitivity and ~90% specificity. The performance of the assay was assessed against the OPXV IgG ELISA in the context of a serosurvey by retrospectively screening a set of serum specimens from the region in Ghana believed to have harbored the MPXV-infected rodents involved in the 2003 United States outbreak.

## 1. Introduction

Mpox is a zoonotic disease caused by exposure to monkeypox virus (MPXV), which was first reported in humans in 1970 in the Democratic Republic of the Congo (DRC) during the smallpox eradication efforts [1]. Until 2003, most outbreaks occurred in the DRC, where the incidence of the infection has increased 20-fold since smallpox eradication [2,3]. In May 2003, an outbreak occurred in the Midwestern United States in individuals who had close contact with pet prairie dogs and other mammals [4,5]. Most recently, MPXV has been associated with multiple clusters internationally, outside traditional endemic areas, including the United States [6,7,8,9]. Albeit less common than in smallpox, human-to-human transmission of MPXV is a concern and has been reported during multiple outbreaks [10] and was the primary driver for the 2022 Mpox outbreak [11,12,13]. Therefore, the emergence of MPXV is a public health issue for populations in endemic and non-endemic areas.

The main line of defense against infection is by vaccination with the Vaccinia virus (VACV), which has an estimated 85% efficacy [14]. However, vaccination ceased worldwide in 1980 [14]. The increasing number of outbreaks [6,7,8,9,10,15] and potential for widespread infection in the context of waning herd immunity and an increasing unvaccinated population warrants epidemiological surveillance of MPXV. Monitoring the incidence and prevalence of infection outside of traditional diagnostic testing may give insight into the extent of human-to-human transmission, geographical spread, and possible mutations leading to increased transmissibility and virulence.

Prior to the 2022 Mpox outbreak, the incidence, prevalence, and human-to-human transmission were unknown because of the difficulty of repeat visits to endemic areas and specific detection of MPXV exposure, and this can still be challenging even in non-endemic areas. A critical barrier to understanding the scope of MPXV infection is the lack of a serological assay that differentiates exposure to MPXV from other OPXV including VACV, variola virus (VARV), and cowpox virus (CPXV) [16,17,18]. OPXV are highly conserved with a >90% similarity at the amino acid level between MPXV and VARV [19]. Existing serological assays are OPXV cross-reactive, and the development of reliable MPXV-specific assays has been difficult [2,16,20,21,22]. OPXV possess various proteins capable of inducing an immunological response, including intracellular mature virion proteins (L1, A17, D8, H3, A13, and A28) and extracellular enveloped virion proteins (A33 and B5) [23,24,25,26,27,28]. Although many of these proteins do not produce neutralizing antibody response [23,29], they represent a unique tool to capture virus-specific antibodies in human sera.

Among many assays used to identify exposure to OPXV, a peptide-based ELISA specific for MPXV antibodies is an optimal choice because it eliminates the need for virus in culture, is more conducive for use in serosurveys in endemic areas and can provide high throughput data. We have previously epitope-mapped MPXV-specific antibodies and demonstrated that slight differences in amino acids can have a dramatic effect on reactivity of an antibody to certain OPXV species [30]. Here, we present the design of a MPXV specific peptide-based ELISA, with optimization through testing a well-characterized population from an Mpox outbreak in the DRC, and implementation via a retrospective analysis of a Ghanian population from the region believed to have housed the mammals implicated in the 2003 U. S. Mpox outbreak. 

## 2. Materials and Methods

### 2.1. ParticipantSelection

#### 2.1.1. 2003 United States Mpox Outbreak Participants

Serum specimens were collected by the CDC during the 2003 U.S. Mpox outbreak. Participants who had not been previously vaccinated and were IgG+ by OPXV ELISA [16] were selected to prescreen the designed MPXV specific peptides. Pooled serum from eight participants was utilized as an internal positive control for the MPXV specific peptide-based ELISA to account for inter-plate variation. 

#### 2.1.2. VARV Outbreak Participants

Serum specimens were aliquoted from archival specimens at the CDC. 

#### 2.1.3. Vaccine Study Participants

Participants were selected from a study conducted at the CDC to assess immunological response to the smallpox vaccine (i.e., VACV). Participants (n = 26) consented to blood collection on day 0 (prior to vaccination), and at an average of 1.5 years post-vaccination. Of the 26 study participants, 24 had received a smallpox vaccination greater than 20 years previously and none had presented with signs or symptoms of previous OPXV infection. The remaining two participants’ sera utilized were collected more than 1 year following primary vaccination. All serum for the study was prescreened by OPXV IgM and IgG ELISA to assess immune status.

#### 2.1.4. DRC Surveillance Participants

Participants were selected from a prospective Mpox observational study in the Kole Region of the DRC in 2007–2011 that was investigated by the U.S. Army Medical Research Institute of Infectious Diseases (USAMRIID) to clinically characterize MPXV infections [31]. Participants who experienced signs and symptoms of MPXV infection (n = 35) consented to donating paired blood samples shortly after rash onset (within 1 month) and at later time points (3–5 months) post-rash onset (PRO). MPXV infection was confirmed in these participants by MPXV specific PCR assay. Serum specimens from these participants were also screened by OPXV IgM and IgG ELISA to determine antibody responses. Participants who did not present with signs and symptoms and who had negative PCR and ELISA results were selected as OPXV naive controls (n = 5). 

#### 2.1.5. Ghanian Study Participants

Participants were selected from a CDC epidemiological study [32] to determine the geographical origin of the MPXV-infected rodents implicated in the 2003 U. S. Mpox outbreak and whether certain rodent species harbor MPXV in nature. Serum samples were received from 90 participants who did not have signs and symptoms of an OPXV infection. The subjects were prescreened by OPXV IgM and IgG ELISA to determine OPXV exposure.

### 2.2. IRB

Use of remainder specimens was reviewed by CDC and was conducted in accordance with applicable federal law and CDC policy. Use of CDC vaccine study specimens (Protocol 3349) was reviewed and approved by CDC Institutional Review Board [33,34,35,36,37]. Ghanian sample collection and testing were approved by CDC IRB (Protocol 4043). Collection and use of Kole MPX specimens (USAMRID FY03-13) was reviewed and approved by the Human Use Committee, USAMRID Command IRB, and the Ethics Committee at University of Kinshasa School of Public Health. 

### 2.3. Peptide Design and Synthesis

Proteins that potentially differentiate immune responses against different OPXV were selected for use in assay design [38]. Specifically, protein microarray results from positive (MPX positive, vaccine naïve) and negative (MPX naïve, vaccine positive) control prairie dogs were compared to identify differential antibody responses at the protein level. Subsequently identified OPXV proteins from several strains of MPXV, VARV, VACV, and CPXV (Table 1) were aligned using Geneious Version 5.4.4 [39], and antigenicity was predicted with the software’s antigenic function utilizing the method of Kolaskar and Tongaonkar [40]. All peptides predicted to be antigenic and specific for MPXV were synthesized at the Biotechnology Core Facility at the CDC containing an N-terminal biotin moiety followed by β-alanine as described previously [30]. All peptides were received as trifluoroacetate (TFA) salts and diluted in water to prepare a stock 5 mg/mL solution.

### 2.4. OPXV ELISA

Serum was assessed for IgG and IgM response with OPXV antigen in accordance with published methods [16]. Serum was heat-treated (56 °C for 1 h) prior to use.

### 2.5. MPXV Peptide ELISA Protocol and Optimization

Peptides (100 µL of 0.5 µg/mL working stocks in phosphate-buffered saline pH 7.4 [PBS]) were coated onto a commercial streptavidin-coated ELISA 96-well plate (Pierce) overnight at 4 °C. PBS (100 µL) was added to half of the plate to be utilized as background for each serum specimen. Wells were then washed 4 times with phosphate-buffered saline with 0.1% Tween 20 pH 7.4 (PBST) and blocked with 1% bovine serum albumin, 1% normal goat serum and 5% nonfat milk in PBS. Serum was diluted in blocking buffer (1:100) and loaded in duplicate. Pooled serum from 8 MPXV-infected individuals from the United States 2003 Mpox outbreak was included in all plates as an internal positive control. After 1 h incubation at room temperature, the plates were washed 4 times with PBST. Horseradish peroxidase-conjugated goat anti-human IgG secondary antibody (KPL) was diluted in blocking buffer (1:5000) and added to each well. After 45 min incubation at room temperature, plates were washed 4 times with PBST and 100 µL TMB substrate solution (KPL) was added to each well. A total of 100 µL of 1% HCl stop solution (KPL) was added to each well to halt the reaction per manufacturer’s instructions. Optical density was recorded at a wavelength of 450 nm (OD_450_) using a SoftMax Pro 5 microplate reader (Molecular Devices).

### 2.6. Statistical Analysis

The signal for each serum specimen was calculated by subtracting the background value for each specimen from the average OD_450_ of duplicate sample wells. The background value was the sum of the average OD_450_ of duplicate background wells for each sample, plus three times the standard deviation of these wells to account for error [41]. Background wells were serum reactivity to the streptavidin-coated wells in the absence of peptide to account for variability across individuals. The signal for each specimen was then normalized for each plate according to the signal obtained from the pooled serum samples from the 2003 United States Mpox outbreak. Sensitivity and specificity were calculated as shown below:

$$\%\;Sensitivity=\left(\;\frac{\#\;of\;True\;Positives}{\#\;of\;True\;Positives+\#\;of\;False\;Negatives}\;\right)*100$$

$$\%\;Specificity=\left(\;\frac{\#\;of\;True\;Negatives}{\#\;of\;True\;Negatives+\#\;of\;False\;Positives}\;\right)*100$$

## 3. Results

### 3.1. Design and Validation of MPXV Specific Peptides

Immunization of prairie dogs with various OPXV vaccines (Dryvax, Acam2000, Imvamune) elicited strong immunological responses to many OPXV proteins [38]. In addition, prairie dogs challenged with MPXV (Congo Basin MPXV-ROC-2003-385) exhibited differential immune profiles compared to prairie dogs immunized with VACV vaccine (Dryvax^®^) [38]. Comparison of these profiles provided characterization of differential immune targets. Proteins with a microarray fluorescence intensity (MFI) of at least 150 units were considered reactive. Proteins with <50% reactivity (i.e., percent of prairie dogs exhibiting immunologic response) with VACV and ≥80% reactivity with MPXV were selected for further analysis (Supplementary Table S1). Proteins not meeting these criteria, but which had strong reactivity (mean MFI > 1500 units) or more than 50% difference of mean MFI between MPXV and VACV, were also selected (Supplementary Table S1). L1 was selected because it is a conserved protein that is required for viral entry into cells and, therefore, predicted to contain immunogenic epitopes [42]. B21 (OPG211) was selected because it was utilized previously in a peptide-based ELISA and, therefore, predicted to be immunogenic [17,43].

To design peptides specific for MPXV, the sequence of each protein (Supplementary Table S1) was aligned for MPXV with various strains of VARV, VACV, and CPXV (Table 1). MPXV strains were selected to include both Clade I and Clade II [44,45]. Sequence alignments along with antigenicity predictions are available in the (Supplementary Material Figures S1–S26). A series of peptides predicted to be antigenic and specific for MPXV (i.e., residues conserved or were substituted in MPXV strains but not found in the consensus OPXV sequence) were synthesized and tagged with biotin (Table 2). An additional peptide from A27 previously used by us [30] was also added to the list of peptides to be screened. As a control, a randomly generated peptide was added to assess nonspecific binding.

**Figure 1:**
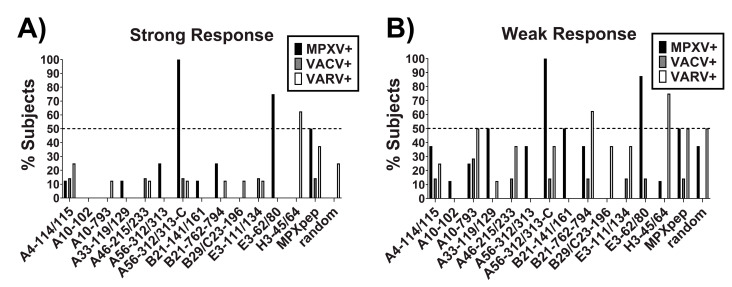
Prescreen of synthetic peptides in a peptide-based ELISA. Synthetic peptides were plated onto a 96-well plate and then incubated with serum samples from subjects known to have been infected with MPXV (n = 8), VACV (n = 7) or VARV (n = 8). Data were analyzed as percentage of subjects eliciting a strong response (**A**, OD450 > 0.3) or a weak response (**B**, OD450 > 0.1). Peptides meeting the criteria of >50% response rate to MPXV+ sera and <50% response rate to VACV+ and VARV+ sera were selected as candidate peptides for developing the MPXV specific peptide-based ELISA.

The peptides were prescreened with sera from OPXV-infected individuals to assess the sensitivity and specificity of each peptide towards MPXV. The sera chosen was from the 2003 United States Mpox outbreak (n = 8; no previous vaccination and OPXV IgG+ by OPXV IgG ELISA), the CDC VACV vaccine study (n = 7; recent and childhood vaccinees), and VARV archival specimens (n = 8; subjects with active or recent smallpox infection at the time of collection). A preliminary peptide concentration of 50 ng per well was utilized based on previous peptide-based ELISA reports [17,43]. The ELISA data obtained were characterized as a strong response (OD_450_ cutoff value [COV] of 0.3) and a weak response (OD_450_ COV of 0.1) of each of the OPXV sera screened (Figure 1). Of the peptides screened, only A56-312/313-C (A56) and E3-62/80 (E3) met our selection criteria of >50% response rate with the MPXV-positive sera and <50% response rate with the VACV- and VARV-positive sera when considering both strong and weak responses (Figure 1A,B, respectively). Although peptide A33-119/129 (A33) did not meet our selection criteria, it captured MPXV-positive sera that were not reported positive by peptide E3 and, hence, peptides A56, A33, and E3 represented a promising combination and were selected for further validation and optimization.

### 3.2. Validation and Optimization of MPXV Specific Peptide-Based ELISA

**Figure 2:**
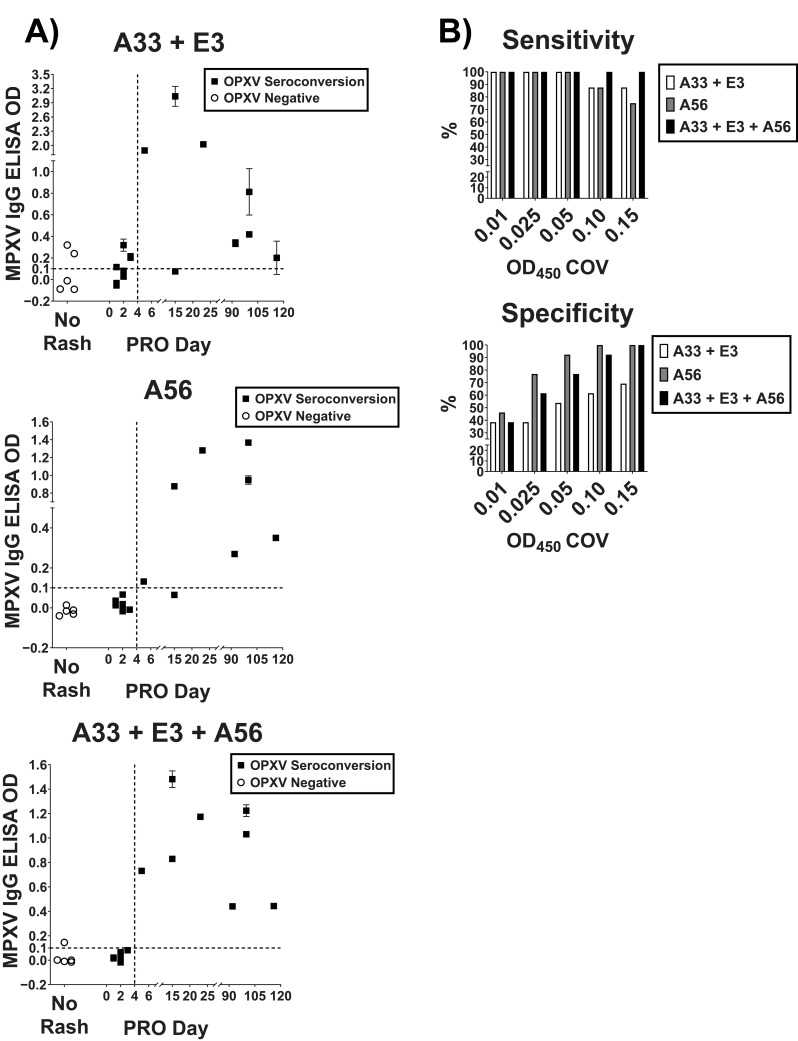
Validation of MPXV specific peptide combinations. Peptides exhibiting specificity in the synthetic peptides prescreen were utilized in a peptide-based ELISA to screen the DRC samples that were OPXV Negative (no signs and symptoms, negative OPXV IgG ELISA result) or OPXV Seroconversion (symptomatic, initial negative OPXV IgG ELISA result followed by a positive result > 4 days PRO). (**A**) MPXV IgG ELISA OD450 values for each of the peptide combinations (A33 + E3 and A33 + E3 + A56) and peptide A56. The 0.1 COV was chosen arbitrarily. (**B**) Sensitivity and specificity percentages for various COVs. For some points, the error bars would be shorter than the height of the symbol. In these cases, the error bars are omitted.

Peptide A56, a combination of peptides A33 and E3, and a combination of all three peptides were used to screen a subset of paired serum samples from a Mpox outbreak in the DRC [31] that were drawn from a clinically well-characterized population at various time points post-rash onset (PRO). The sera were first screened with the OPXV IgG ELISA to determine whether subjects had produced an antibody response to OXPV [31]. Three types of participant groups were observed. First, participants that showed no signs and symptoms of infection and were OPXV IgG negative were labeled “OPXV Negative”. Second, participants that were symptomatic and had an initial negative OPXV IgG ELISA result followed by a positive result > 4 days PRO were labeled “OPXV Seroconversion”. Third, participants that were symptomatic and had a positive OPXV IgG ELISA result were labeled “OPXV Positive”. To perform a preliminary analysis of the selected MPXV-specific candidate peptides in a peptide-based ELISA, we selected the OPXV Negative and OPXV seroconversion specimens. The assay was performed using the peptides A33 and E3, peptide A56, and a combination of all three peptides (Figure 2A). The combination of all three peptides gave the best sensitivity without significantly decreasing specificity at multiple OD_450_ COVs (Figure 2B).

**Figure 3:**
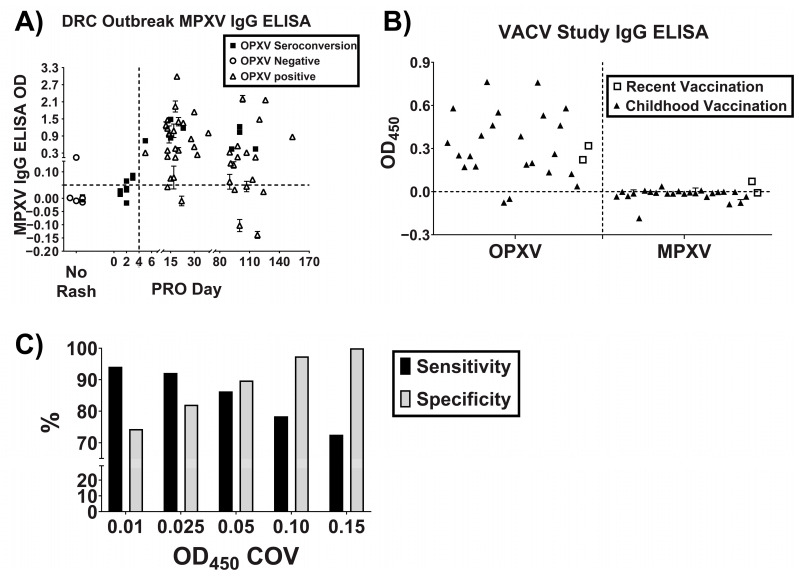
Optimization of MPXV specific peptide-based ELISA. Combination of peptides A33 + E3 + A56 were utilized in a peptide-based ELISA to screen additional samples towards determining an optimal ELISA OD450 COV. (**A**) MPXV IgG peptide-based ELISA OD450 values for all of the DRC outbreak samples, including OPXV Negative (no signs and symptoms, negative OPXV IgG ELISA result), OPXV Seroconversion (symptomatic, initial negative OPXV IgG ELISA result followed by a positive result > 4 days PRO), and OPXV Positive (symptomatic, positive OPXV IgG ELISA result and did not have an initial negative result). (**B**) OPXV IgG ELISA and MPXV peptide- based ELISA data for the vaccine study samples including childhood (n = 24) and recent (n = 2) VACV vaccinees. **(C)** Sensitivity and specificity percentages for various COVs based on the DRC outbreak samples as well as the vaccine study samples. For some points, the error bars would be shorter than the height of the symbol. In these cases, the error bars are omitted.

Therefore, it was decided to proceed with testing additional serum samples using the combination of all three peptides to identify a reliable OD_450_ COV and to further assess the sensitivity and specificity of the assay. All of the DRC outbreak specimens were screened with the MPXV peptide-based ELISA and stratified by PRO-day to investigate the IgG response further (Figure 3A). One of the most common drawbacks of MPXV serological assays is cross-reactivity with sera from previously vaccinated individuals. To help assess this cross-reactivity, serum specimens from an ongoing VACV vaccine study were included. Participants included childhood as well as recent vaccine recipients. The sera were first screened with the OPXV IgG ELISA, and almost all were OPXV IgG-positive, with the exception of two specimens that were equivocal (Figure 3B). The vaccine study specimens were screened with the MPXV peptide-based ELISA, and all samples were non-reactive (Figure 3B). Taken together, a sensitivity of ~86% and a specificity of ~90% using an OD_450_ COV of 0.05 was achieved (Figure 3C). Serum specimens with an OD_450_ value below 0.05 and a standard deviation that crossed the COV were considered equivocal.

### 3.3. Retrospective Analysis of a Population with Unknown MPXV Exposure

**Figure 4:**
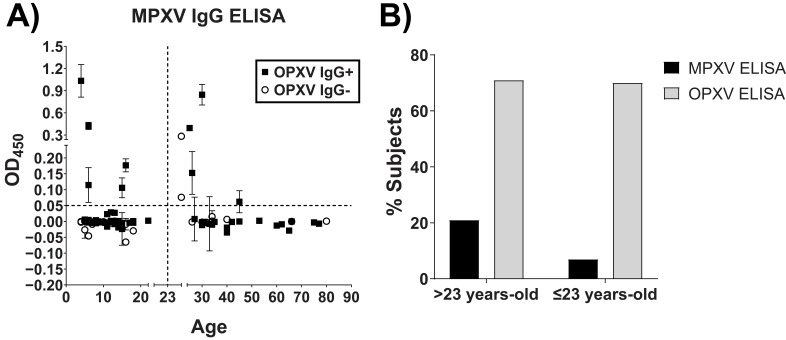
MPXV exposure characterization of the Ghanian population. (**A**) MPXV IgG ELISA screening. MPXV peptide-based IgG ELISA was conducted with the peptide combination of A33 + E3 + A56 (50 ng/well) and an optimized OD450 COV of 0.05. (**B**) Percentage of samples positive by the MPXV-specific IgG ELISA compared with the OPXV IgG ELISA [32]. For some points, the error bars would be shorter than the height of the symbol. In these cases, the error bars are omitted.

A subset of 90 serum samples from a Ghanian population (Table 3) with suspected exposure to MPXV, or other OPXV, were retrospectively analyzed using the MPXV specific peptide-based ELISA [32]. This population had no known recent MPXV infection as determined by the absence of active or recalled signs and symptoms and negative OPXV IgM. The serum samples were subsequently assessed using the MPXV-specific peptide-based ELISA and were stratified by age (23 years was the cutoff at the time of specimen collection for the likelihood of a subject being previously vaccinated with VACV for this population) as well as OPXV IgG status (Figure 4A and Table 3). Analysis of the population older than 23 years old (n = 34) showed that a significantly lower proportion of subjects tested positive for exposure to MPXV using the peptide-based ELISA (n = 7, 21%) compared with exposure to OPXV using whole virus (n = 24, 71%) (Figure 4B). Similar results were obtained when analyzing the population that were 23 years old and younger (n = 56) with 7% (n = 4) who were determined to have been exposed to MPXV compared with 70% (n = 39) exposed to OPXV (Figure 4B). Of the 11 participants testing positive for MPXV exposure, only 2 participants tested negative for OPXV exposure by the OPXV IgG ELISA. One of these two participants was an animal trapper who was determined to have had extended exposure to animals possibly infected with MPXV after epidemiological interviews [32]. Only 2 participants were determined to be equivocal by the MPXV-specific peptide-based ELISA and one of these subjects was an animal caretaker who also had extended exposure to animals possibly infected with MPXV.

## 4. Discussion

Historically, characterization of exposure to OPXV has been performed with multiple assays, including hemagluttination inhibition [21], immunofluorescence [20], Western blot [18], radioimmunoassay [21,22], and IgM and IgG ELISA [16,17,18,39]. These assays suffer from limitations including the need for culturing virus, highly skilled laboratorians, advanced equipment, and reagents. Additionally, some infected individuals may not immediately seek medical attention, and after the infection has resolved, serological assays would not be capable of determining the specificity of the OPXV exposure. There is a need for reliable assays that can retrospectively define the scope of an outbreak. 

Given these limitations and the advances in peptide synthesis and ELISA technology, a peptide-based ELISA would better serve as a serological assay to characterize exposure to MPXV. Peptide-based serological assays are well-established and have been utilized for specific identification of a myriad of viral and bacterial infections [47,48,49,50,51,52]. A peptide-based ELISA has been reported previously utilizing proteins that were found in MPXV but not in VACV (D2L, B18R, N2R, N3R, and B21R) [17,43,53]. However, the assay has not been tested with sera from other OPXV and the proteins selected were part of the VARV and CPXV proteomes [54]. As a result, the peptides were likely cross-reactive with VARV and CPXV and the assay may not have been effective in OPXV endemic areas including Europe (CPXV) [55,56] and Africa (MPXV) [2,57]. Here, we presented the design of a robust peptide-based IgG ELISA capable of detecting MPXV specifically and suitable for serosurveys in endemic areas. IgG is the ideal target because it peaks relatively early in the course of infection, or as early as two days post-rash onset [16], and remains abundant for decades in previously infected individuals [58,59,60,61,62].

Humoral characterization of VACV vaccination and MPXV challenge in prairie dogs led to the selection of 26 OPXV proteins predicted to have antigenic epitopes specific for MPXV. We used a bioinformatics approach to design peptides predicted to be both antigenic and specific for MPXV for each of these OPXV proteins. This yielded a total of thirteen synthetic peptides from nine different OPXV proteins. We demonstrated the validity of the peptides by utilizing them in an indirect ELISA format and screened sera from individuals known to have been exposed to MPXV, VARV, or VACV. Contrary to previously reported assays, we assessed the background binding of each serum sample to the plate surface individually, considering the inherent variation between human sera. Inter-plate variation was also accounted for by normalizing the signal from each sample to an internal positive control composed of pooled sera from the 2003 United States Mpox outbreak. We performed the initial experiments with MPXV-, VARV-, or VACV-positive sera to rule out cross-reactivity with other OPXV. In endemic areas, vaccination efforts ceased in 1980 and there is a high chance of encountering individuals with prior vaccination and, therefore, a higher chance of cross-reactivity. Exposure to VARV is another cross-reactivity concern that we wanted to account for due to the potential of encountering smallpox survivors and in the event of a reemergence of this virus. The peptide prescreen demonstrated that only two of the peptides reacted strongly with the MPXV positive sera and were minimally cross-reactive with VACV and VARV sera. One additional peptide was selected as it captured some of the MPXV positive sera that were not captured by the other two peptides. After further optimization, we elected to use a combination of all three peptides to provide a higher recognition of anti-MPXV human IgG. A concern of using a combination of peptides would be an increase in nonspecific binding of IgG. To overcome this, we maintained the total peptide concentration at 50 ng. We determined the optimum concentration of the synthetic peptides to be 50 ng per well; increasing the concentration increased sensitivity but resulted in a drastic decrease in specificity (Figure S27).

The validity of the assay was confirmed by correlation with the results of the OPXV IgG ELISA screen of a Mpox outbreak in the DRC in which the participants (not previously vaccinated) were PCR-confirmed to have been infected with MPXV. The patient population was well-characterized clinically and diagnostically [31]. The assay captured the seroconversion of subjects that provided paired serum samples early (<4 days) and late (>4 days) PRO. Some serum samples had relatively high OD_450_ values and would be considered positive at a lower COV. After repeating the screening for these samples and ruling out assay variation or error, we speculated that the reason some of the samples had higher OD_450_ values might have been due to patients reporting the rash later than the initial symptom onset. That is, some of the samples who had drawn early PRO might have already started to experience seroconversion. Some of the serum later PRO samples collected were negative in our assay and this could have been due to slight decrease in IgG response after the disease resolved [43] or to low reactivity with the peptide MPXV epitopes. The specificity of the assay was tested further by screening a set of serum samples from a CDC VACV vaccine study that included childhood vaccinees and greater than one year post vaccination. With an OD_450_ COV of 0.05, we were able to achieve a sensitivity of 86.3% and specificity of 89.7% with the peptide-based MPXV IgG ELISA utilizing data from both the DRC outbreak samples as well as the VACV vaccine study samples. This evidence supported the specificity of the assay and its utility in serosurveys.

The performance of the MPXV-specific peptide-based ELISA was assessed in the setting of a serosurvey by screening a set of serum specimens from the regions in Ghana believed to have harbored the MPXV-infected rodents involved in the 2003 United States outbreak [32]. We screened a cohort of 90 participants (Table 3) who were believed to be unvaccinated previously (through epidemiologic interviews as well as inspections for a vaccination scar) and were not recently infected with an OPXV, as confirmed by the OPXV IgM ELISA. The age of the participants ranged from 4 to 80 years old and covered the anticipated age when vaccination efforts ceased. Participants older than 23 years old (n = 34) might have received a smallpox vaccination, while participants 23 years old or younger (n = 56) were unlikely to have been vaccinated for smallpox. Of the >23-year-old (n = 34) and ≤23-year-old participants (n = 56), a significantly lower percentage were determined to have been exposed to MPXV (21% and 7% vs. 71% and 70%) vs. OPXV, respectively. Of the total 63 participants positive for OPXV IgG, it is likely that 39 participants might have had exposure to MPXV. These participants were ≤23 years-old and, therefore, likely unvaccinated, and their IgG levels were lower compared with Mpox patients 1 year after infection [32]. Of the 11 participants who tested positive or equivocal for MPXV exposure, only two participants tested negative for OPXV exposure via the OPXV IgG ELISA. One of the participants was a resident of an affected community. The other participant was an animal trapper, suggesting that MPXV exposure had likely occurred, given the common occupational exposure to animals and the fact that several animal species tested positive for OPXV by PCR [32]. The average age for the individuals who were MPXV-positive or equivocal by the MPXV IgG ELISA was 21.6 years. One explanation for the discrepancy within this age group between the OPXV IgG ELISA and our assay is that the OPXV IgG ELISA calculated background for the entire plate, while our assay calculated not only general plate background, but also the background attributed to each individual specimen, thereby reducing any concerns for variability between individuals. Other possible explanations that we cannot rule out are that the exposure to MPXV could have led to a sub-clinical infection, yet still produced a serological response measured by OPXV IgG ELISA, exposure to a previously undetected circulating OPXV or that the time post-exposure for the retrospective analysis was longer than seen with the sample set used to validate the assay causing a loss in sensitivity over time with the MPXV IgG ELISA. 

There were several limitations to the development of this assay. First, the lack of availability of serum samples from participants actively infected with or previously exposed to CPXV. This would have provided further validation that the assay was specific to MPXV and not any of the other OPXV known to infect humans (VACV, VARV, and CPXV). Second, the analysis of OPXV proteins predicted to have MPXV-specific epitopes was performed bioinformatically and not experimentally. A third limitation was that the dilution factor for the sera was not investigated fully. However, in our hands, serum dilution of 1:100 provides optimal signal and is in line with previous work [16]. Lastly, a potential limitation is the fact that the serum specimens tested to validate the assay were from individuals with a recent MPXV exposure, but it had been a long time since their vaccination. This suggests that the validated assay may not perform equally in recently vaccinated individuals and/or those with a long time since MPXV exposure. However, utilizing the assay to screen the Ghanian population, which did not have a history of recent MPXV exposure, demonstrated that the assay may still be useful in similar populations. Further studies should be considered to address these limitations.

In summary, the work presented here validates the utility of a novel peptide-based IgG ELISA in detecting exposure to MPXV, specifically in the context of a serosurvey. We demonstrated the assay’s ability in capturing seroconversion in patients actively infected with MPXV. We also showed that the assay did not cross-react with sera from vaccinated participants (1.5–30+ years post-vaccination) or smallpox survivors. The application of the assay in screening a population suspected to have been exposed to MPXV demonstrated the additional depth of information the assay provided to currently used assays in serosurveys.

## 5. Disclaimer

Opinions, interpretations, conclusions, and recommendations are those of the authors and are not necessarily endorsed by DOD or the Centers for Disease Control and Prevention. Research on human participants was conducted in compliance with U.S. Department of Defense, federal, and state statutes and regulations relating to the protection of human participants &and adheres to principles identified in the Belmont Report (1979). 

## Figures and Tables

**Table 1:** OPXV strains selected for alignment and prediction of MPXV specific antigenic peptides.

OPXV Strain	Place of Isolation	Year	Clinical Isolate	Host	Accession Number
CPXV-AUS1999-867	Texing, Austria	1999	Local lesions	Cat	HQ407377
CPXV-BR	Brighton, United Kingdom	1937	Local lesions	Human	NC_003663
CPXV-GER1980	Hameln, Germany	1980	Local lesions	Elephant	HQ420895
CPXV-GER1998-2	Eckental, Germany	1998	Local lesions	Human	HQ420897
CPXV-GER2002-MKY	Göttingen, Germany	2002	Fatal generalization	Marmoset	HQ420898
CPXV-GER1991	Munich, Germany	1991	Local lesions	Human	DQ437593
CPXV-GRI	Moscow, Russia	1990	Local lesions	Human	X 94355
CPXV-GER1990	Bonn, Germany	1990	Fatal generalization	Human	HQ420896
MPXV-COG2003-358	Impfondo, Republic of the Congo	2003	Local lesions	Human	DQ011154;
MPXV-COP58	Copenhagen, Denmark	1958	Local lesions	Monkey	AY753185
MPXV-USA2003-039	Wisconsin, USA	2003	Local lesions	Human	DQ011157
MPXV-WR267	Washington, DC, USA	1962	Local lesions	Monkey	AY603973
MPXV-ZAR	Kasai Oriental, Zaire	1996	Local lesions	Human	NC_003310
MPXV-ZAR-1979-005	Equateur, Zaire	1978	Local lesions	Human	DQ011155
VACV-ACAM2000	New York, USA	2004	Cell culture	Cow (Dryvax)	AY313847
VACV-COP	New York, USA	1913	Cell culture	Cow (Dryvax)	M35027
VACV-MVA	Munich, Germany	1971	Chicken embryo fibroblasts culture	-	DQ983236
VARV-BEN68	Benin	1968	Local lesions	Human	DQ441416
VARV-BGD-Banu	Bangladesh	1975	Local lesions	Human	DQ437581
VARV-JPV51-hrpr	Japan	1951	Local lesions	Human	DQ441430

**Table 2:** Characteristics of MPXV specific synthetic peptides.

Peptide	Protein	Sequence	Substitution(s)	Length
A4-114/115	A4 (OPG130)	Biotin-β-Ala-PTPAILLPSSTAPVLKPRQQTNT	T114V, P115L	23
A10-102	A10 (OPG136)	Biotin-β-Ala-NAGNIDIINHPINISSETNPIIN	T102H	23
A10-793	A10 (OPG136)	Biotin-β-Ala-TIERIFNAKVCDDVKASMLEKY	G793C	22
A33-119/129	A33 (OPG161)	Biotin-β-Ala-GSCYILHSDYKSFEDAKANCAAESS	Q119K, L120S, S122E, T129A	25
A46-215/223	A46 (OPG176)	Biotin-β-Ala-LRGHTDSIEDEFDHFEDDDSST	E215D, Y223H	22
A56-312/313	A56 (OPG185)	Biotin-β-Ala-SAVAIFCITYYICNKHPRKYKTENKV	R312H, S313P	26
A56-312/313-C *	A56 (OPG185)	Biotin-β-Ala-SAVAIFCITYYICNKHPRKYKTENKV	R312H, S313P	26
B21-141/161	B21 (OPG211)	Biotin-β-Ala-TVITTEELQVTPTYAPVTTPLPTSAVPYDQRS	K141Q, S145T, P146Y, N149del, T152P, S161A	32
B21-762/794	B21 (OPG211)	Biotin-β-Ala-GLQSPNPPLRNPLPQHDDYSPPQVHRPPP	N762del, P763del, P764del, P765del, Y766del, R767del, Q768P, R771Q, G772H, Y777S, S794P	29
B29/C23-196	B29/C23 (OPG1)	Biotin-β-Ala-GSNISHKKVSYKDIIGSTIVDTK	E196K	23
E3-62/80	E3 (OPG65)	Biotin-β-Ala-SSDDTPPRWSTTMDADTRPTDSDADAIIDD	I62T, F66S, M67T, T70M, E71D, K74T, P75R, D76P, A77T, A79S, M80D	30
E3-111/134	E3 (OPG65)	Biotin-β-Ala-VIPVKKIIYWKGVNPVTVINEYCQITRRRDWS	A111V, D116Y, D119G, A120V, I125V, K134R	32
H3-45/64	H3 (OPG108)	Biotin-β-Ala-VKDNEVMQEKRDVVIVNDDPDHYKDYVF	P45Q, N49D, K54N, A64V	28
MPXpep	A27 (OPG154)	Biotin-β-Ala-TEFFSTKAAKNPETKREAIVKAYGDDNEETLKQ	K27N, A30T, D39Y	34
Random *	-	Biotin-β-Ala- VTIKEYTATQRKLNFNEKDKESPEAADKTAEGF	-	33

* Peptides that contain a C-terminus amide group. Red text indicates locations of amino acids unique to MPXV. Protein names refer to VACV Copenhagen strain. Orthopoxvirus genes (OPG) are indicated according to the recently reported classification [46].

**Table 3:** Characteristics of Ghanian subjects associated with OPXV and MPXV IgG results.

Age (years)	OPXV IgG ELISA		MPXV Peptide IgG ELISA	
	No. (%) Positive	No. (%) Negative	No. (%) Positive	No. (%) Negative
≤23	39 (70%)	17 (30%)	4 (7%)	52 (93%)
>23	24 (71%)	10 (29%)	7 (21%)	27 (82%)

## Data Availability

All data are made available through the manuscript or the Supplementary Materials.

## Supplementary Materials

The following supporting information can be downloaded at: https://www.mdpi.com/article/10.3390/pathogens12030396/s1, Figure S1–26: sequence alignments for MPXV proteins. Figure S27. Optimization of peptide concentration for MPXV peptide-based ELISA. Table S1: Analysis of OPXV proteins immunogenicity after vaccination with VACV or challenge with MPXV.
