# Supplementary material for: Design and Optimization of a Monkeypox virus Specific Serological Assay
Source: Pathogens. 2023 Mar 1;12(3):396. doi: 10.3390/pathogens12030396 (PMC10054672; doi:10.3390/pathogens12030396)
Supplement: Supplementary file 1 [file pathogens-12-00396-s001.zip › pathogens-2219265-SI.pdf]

pathogens

Supplementary Material

Design and Optimization of a *Monkeypox virus* Specific Serological Assay

Taha Y Taha[1], Michael B. Townsend[3], Jan Pohl[2], Kevin L Karem[3], Inger K Damon[3], Placide Mbala Kingebeni[4], Jean-Jacques Muyembe Tamfum[4], James W Martin[5], Phillip R. Pittman[5], John W Huggins[5], Panayampalli Subbian Satheshkumar[3], Dennis A Bagarozzi, Jr.[1], Mary G. Reynolds[3], Laura Hughes[1].

[1] Reagent and Diagnostic Services Branch, Division of Scientific Resources, National Center for Emerging and Zoonotic Infectious Diseases, Centers for Disease Control and Prevention

[2] Biotechnology Core Facility Branch, Division of Scientific Resources, National Center for Emerging and Zoon-otic Infectious Diseases, Centers for Disease Control and Prevention

[3] Poxvirus and Rabies Branch, Division of High Consequence Pathogens and Pathology, National Center for Emerging and Zoonotic Infectious Diseases, Centers for Disease Control and Prevention

[4] Institut National de Recherche Biomédicale, Ministère de la Santé Publique, Kinshasa-Gombe B.P. 1197, Democratic Republic of the Congo (DRC)

[5] Department of Clinical Research, Division of Medicine, U.S. Army Medical Research Institute of Infectious Diseases (USAMRIID), Fort Detrick, MD, USA.

* Correspondence: Laura Hughes; bkz2@cdc.gov

Table of Contents

Table S1. Analysis of OPXV proteins immunogenicity after vaccination with VACV or challenge with MPXV......4

Figure S1. Sequence alignment for protein A4. 5

Figure S2. Sequence alignment for protein A10. Page 1 of 3......6

Figure S3. Sequence alignment for protein A11. Page 1 of 2......9

Figure S4. Sequence alignment for protein A13. 11

Figure S5. Sequence alignment for protein A14. 12

Figure S6. Sequence alignment for protein A25. Page 1 of 3......13

Figure S7. Sequence alignment for protein A26. Page 1 of 3......16

Figure S8. Sequence alignment for protein A33. 19

Figure S9. Sequence alignment for protein A34. 20

Figure S10. Sequence alignment for protein A46. 21

Figure S11. Sequence alignment for protein A56. 22

Figure S12. Sequence alignment for protein B5. Page 1 of 2......23

Figure S13. Sequence alignment for protein B21-B22. Page 1 of 12......25

Figure S14. Sequence alignment for protein B29-C23. Page 1 of 3......37

Figure S15. Sequence alignment for protein D8. Page 1 of 2......40

Figure S16. Sequence alignment for protein D13. Page 1 of 3......42

Figure S17. Sequence alignment for protein E3......45

Figure S18. Sequence alignment for protein F2......46

Figure S19. Sequence alignment for protein F13. Page 1 of 2. 47

Figure S20. Sequence alignment for protein H3. Page 1 of 2......49

Figure S21. Sequence alignment for protein H5. Page 1 of 2......51

Figure S22. Sequence alignment for protein H6. Page 1 of 2......53

Figure S23. Sequence alignment for protein I1. Page 1 of 2. 55

Figure S24. Sequence alignment for protein I3. Page 1 of 2. 57

Figure S25. Sequence alignment for protein L1......59

Figure S26. Sequence alignment for protein L4. Page 1 of 2. 60

Figure S27. Optimization of peptide concentration for MPXV peptide-based ELISA. **62**

References: **62**

Table S1. Analysis of OPXV proteins immunogenicity after vaccination with VACV or challenge with MPXV.

Protein Function/Location	OPXV Protein[f]	Mean MFI (units)[a]		% Response[b]		% Difference of mean MFI[e]
		Post-MPXV Challenge[c] (n = 5)	Post-VACV Vaccination[d] (n = 7)	Post-MPXV Challenge (n = 5)	Post-VACV Vaccination (n = 7)	
**Intracellular Mature Virion**	A13	1,0346.8	2360.6	100.0	85.7	125.7
	A14	946.8	92.7	80.0	28.6	164.3
	A25	3,1574.0	16602.4	100.0	100.0	62.2
	A26	391.0	147.3	80.0	28.6	90.5
	D8	6934.4	2011.6	100.0	100.0	110.1
	H3	6755.0	1788.8	100.0	100.0	116.3
**Extracellular Enveloped Virion**	A33	150.2	−1.7	40.0	0.0	204.7
	A34	665.4	101.1	80.0	14.3	147.3
	A56	1734.0	2039.8	100.0	100.0	−16.2
	B5	3950.8	126.7	100.0	42.9	187.6
	F13	353.6	21.4	80.0	14.3	177.2
**Core/Enzyme**	A4	6658.8	208.2	100.0	42.9	187.9
	A10	3013.4	814.4	100.0	85.7	114.9
	F2	1405.2	58.3	100.0	14.3	184.1
	H5	265.4	−22.0	60.0	14.3	236.1
	H6	230.0	88.7	60.0	14.3	88.7
	I1	1,5396.8	6170.9	100.0	100.0	85.6
	I3	128.4	53.0	40.0	0.0	83.1
	L4	1411.2	211.9	100.0	42.9	147.8
**Evasion/Virulence**	A46	258.2	7.4	40.0	0.0	188.9
	B29	2841.4	64.7	100.0	28.6	191.1
	E3	342.6	505.7	80.0	85.7	−38.5
**Other**	A11	3580.8	443.1	100.0	57.1	156.0
	B21–22	-	-	-	-	-
	D13	8270.0	1735.4	100.0	100.0	130.6
	L1	-	-	-	-	-

[a] Data extracted with permission from Townsend et al. [38] [b] Percentage of prairie dogs eliciting an immune response to the OPXV protein as detected by the microarray. [c] Challenge was performed with Congo Basin MPXV-ROC-2003-385. [d] Vaccination was performed with Dryvax® vaccine. [e] Percentage difference was calculated as 100 times (MPXV mean MFI – VACV mean MFI)/Average (MPXV mean MFI, VACV mean MFI). [f] protein names refer to VACV Copenhagen strain.

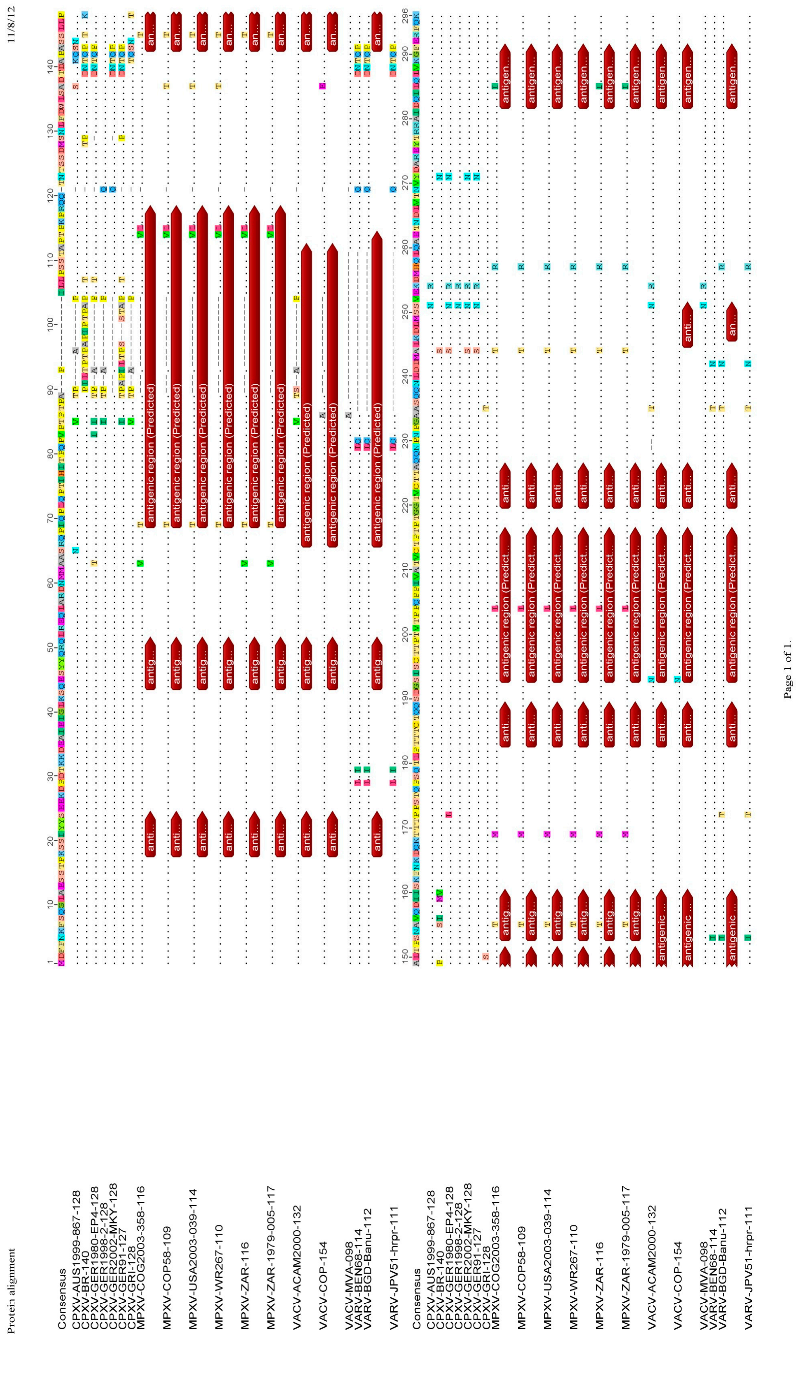

Figure S1. Sequence alignment for protein A4.

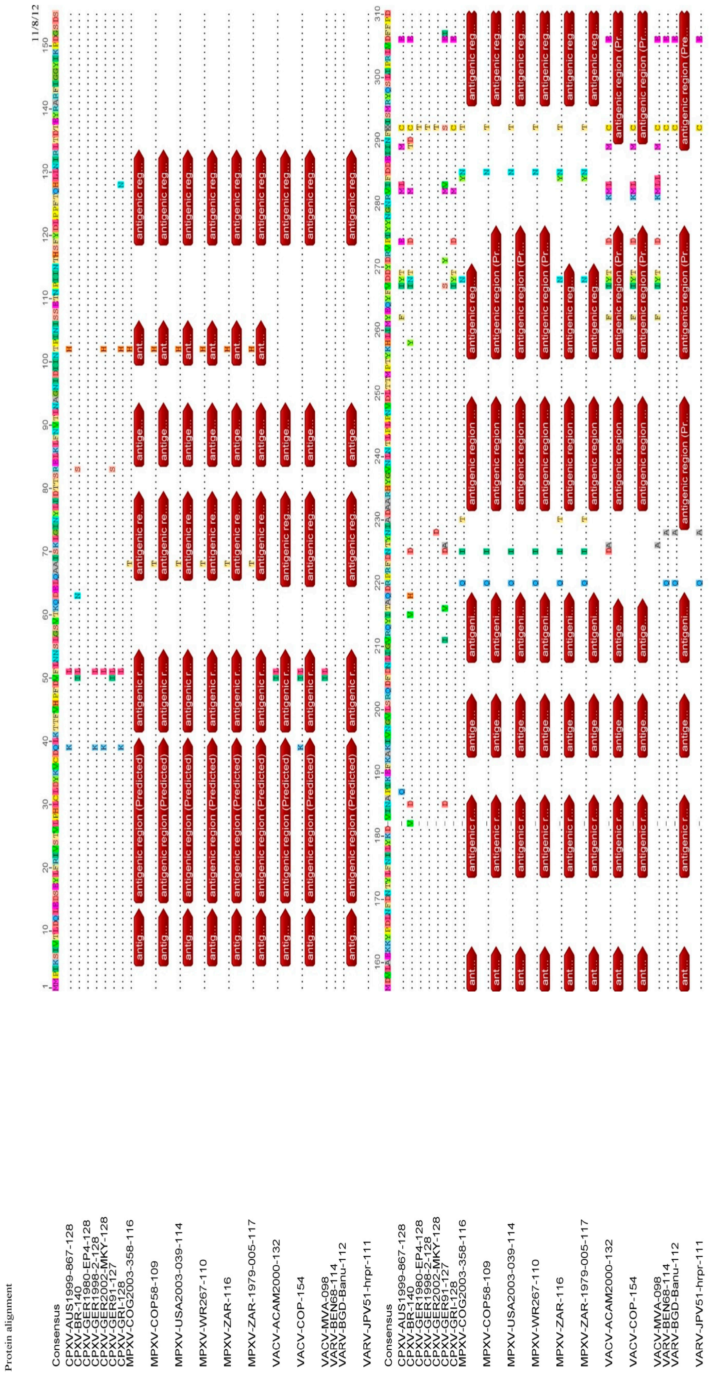

Figure S2. Sequence alignment for protein A10. Page 1 of 3.

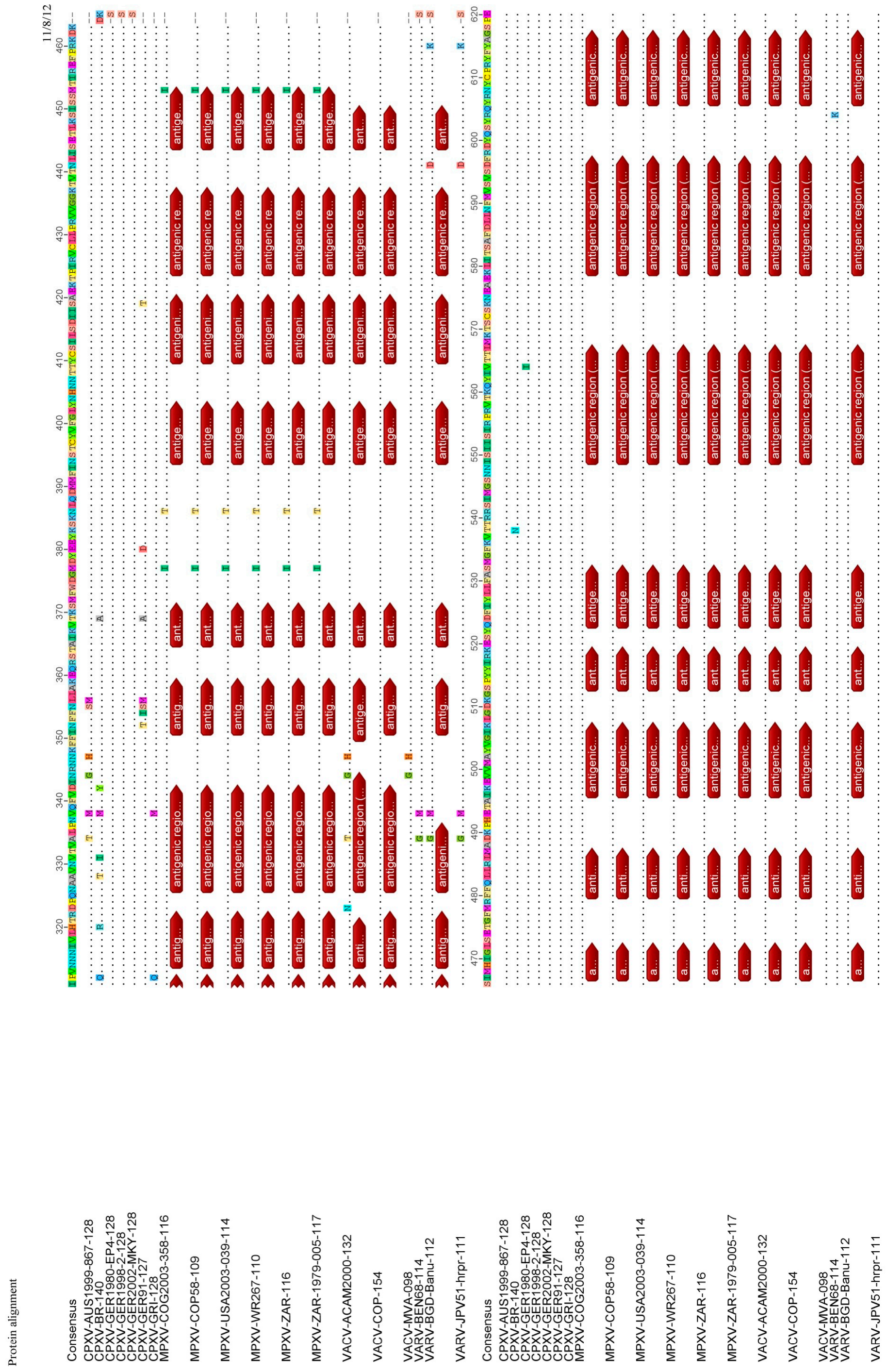

Figure S2. Sequence alignment for protein A10. Page 2 of 3.

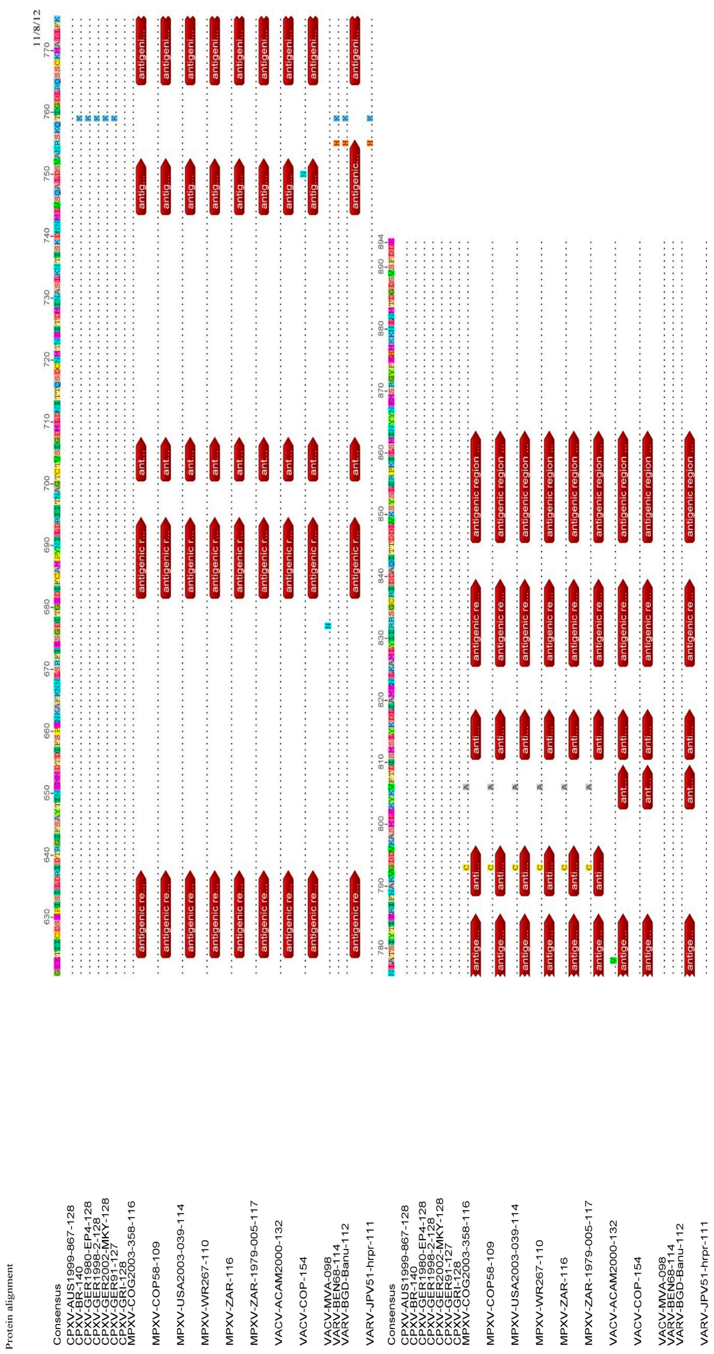

Figure S2. Sequence alignment for protein A10. Page 3 of 3.

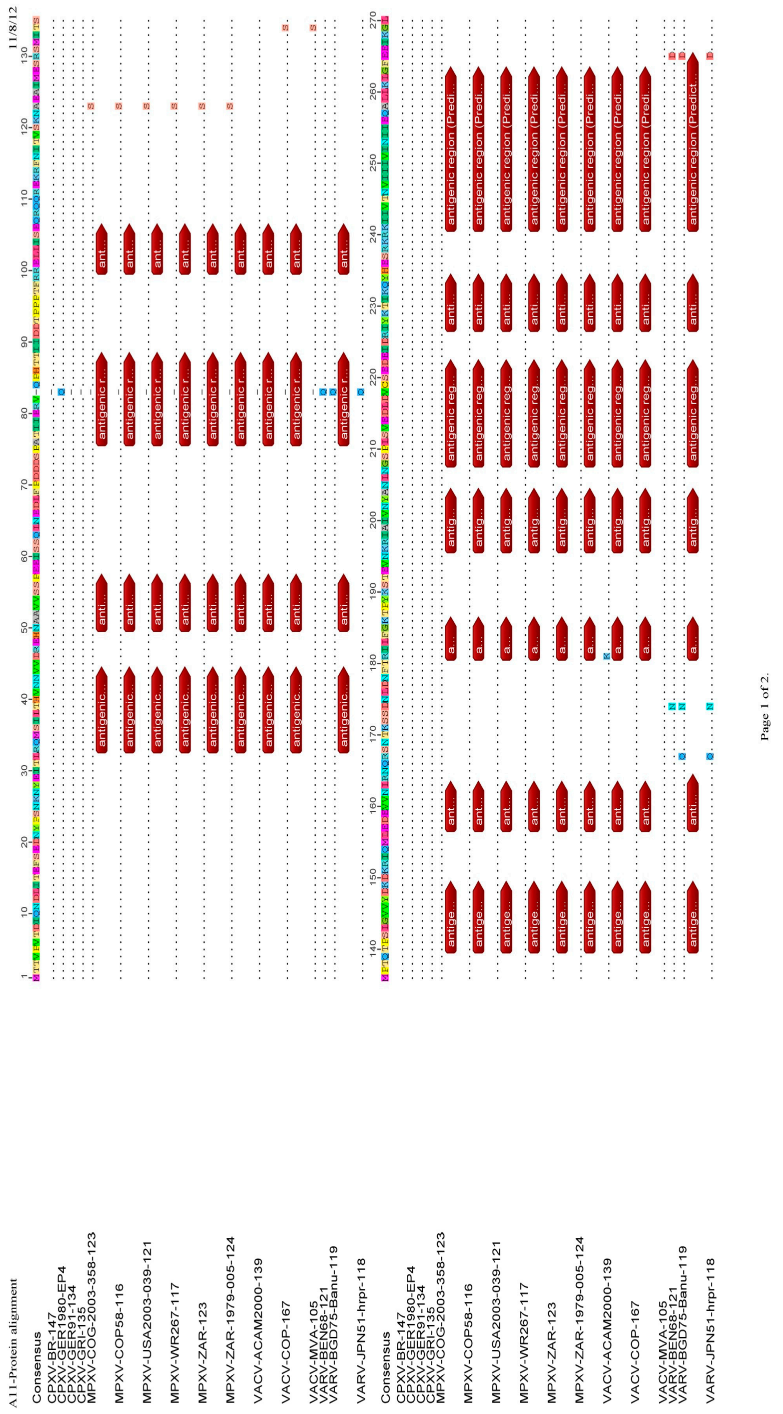

Figure S3. Sequence alignment for protein A11. Page 1 of 2.

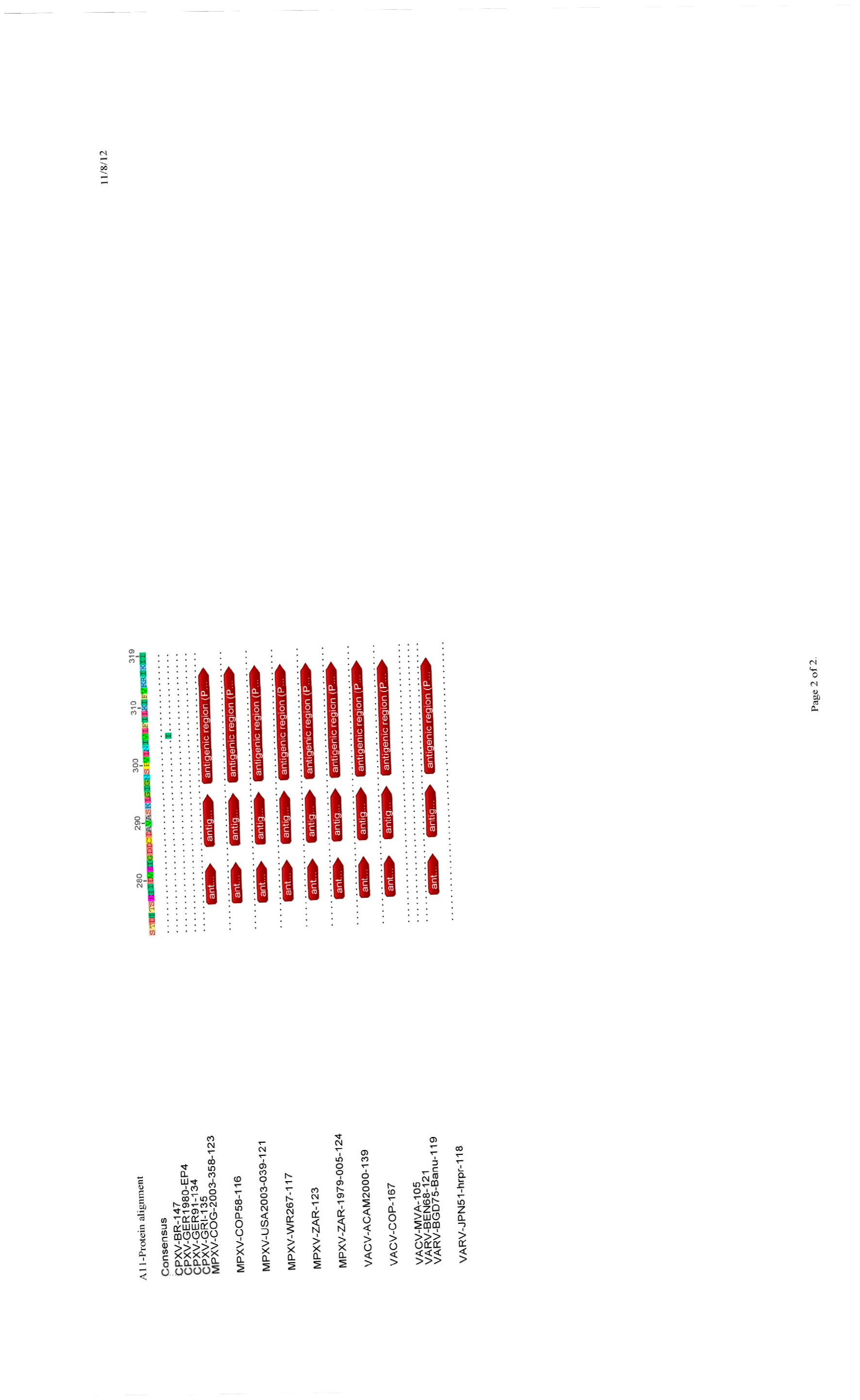

Figure S3. Sequence alignment for protein A11. Page 2 of 2.

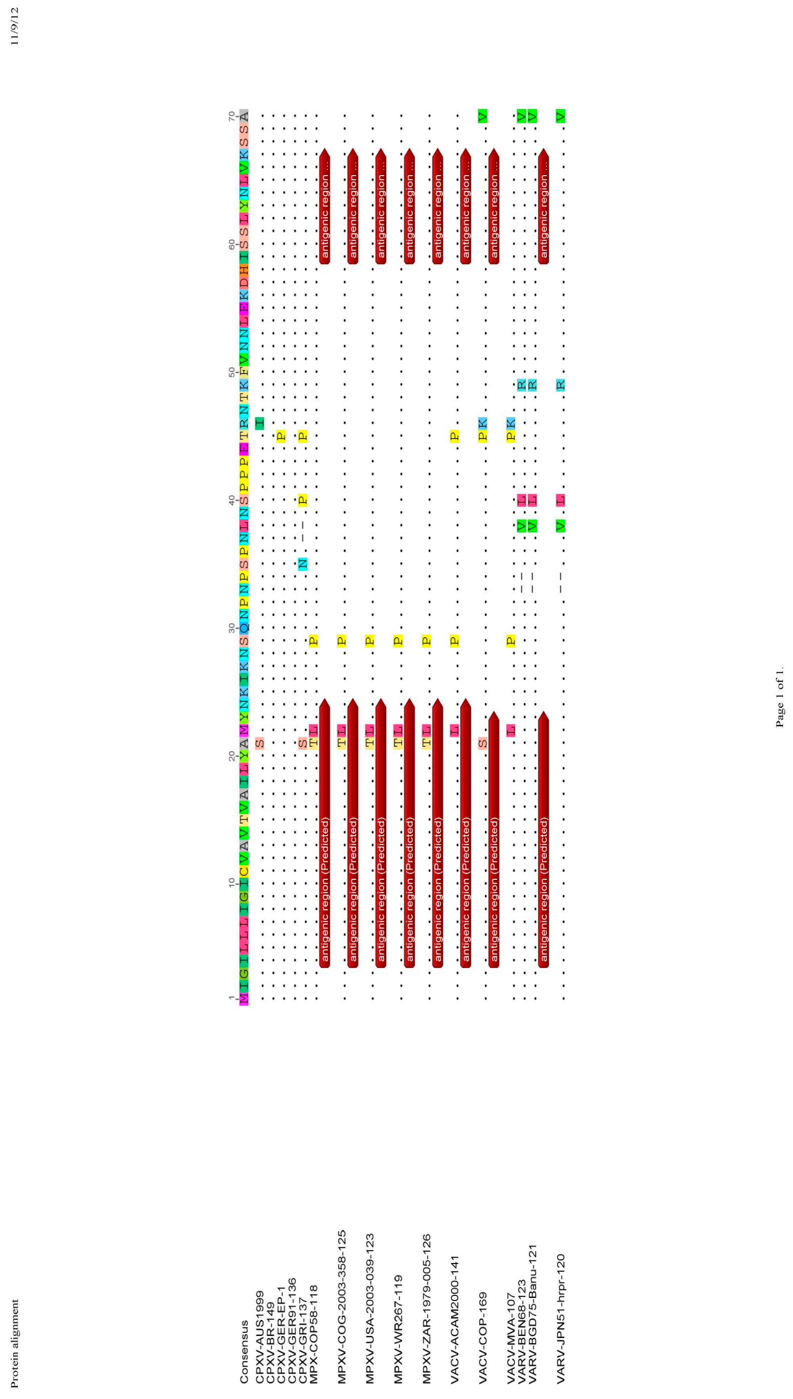

Figure S4. Sequence alignment for protein A13.

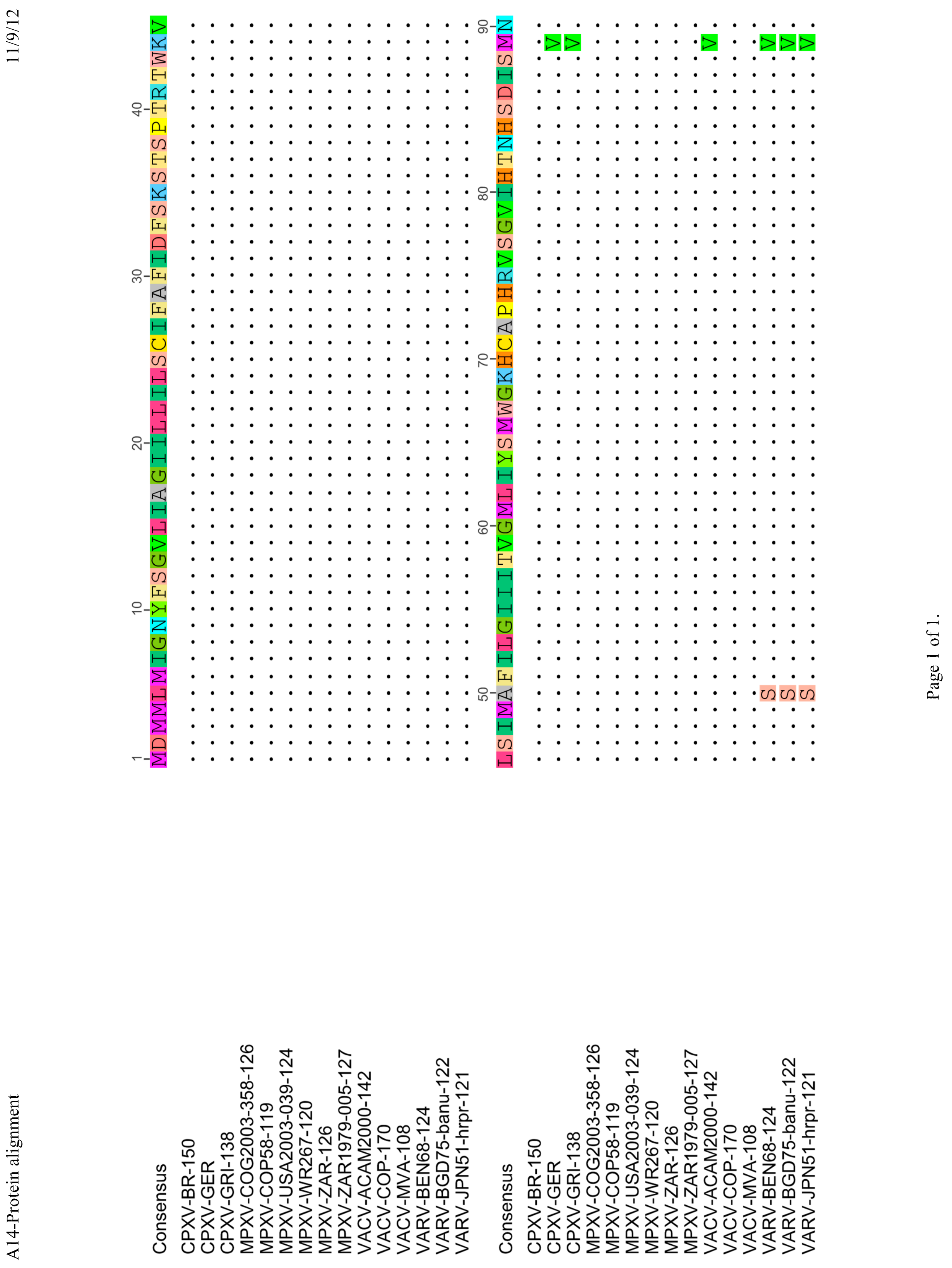

Figure S5. Sequence alignment for protein A14.

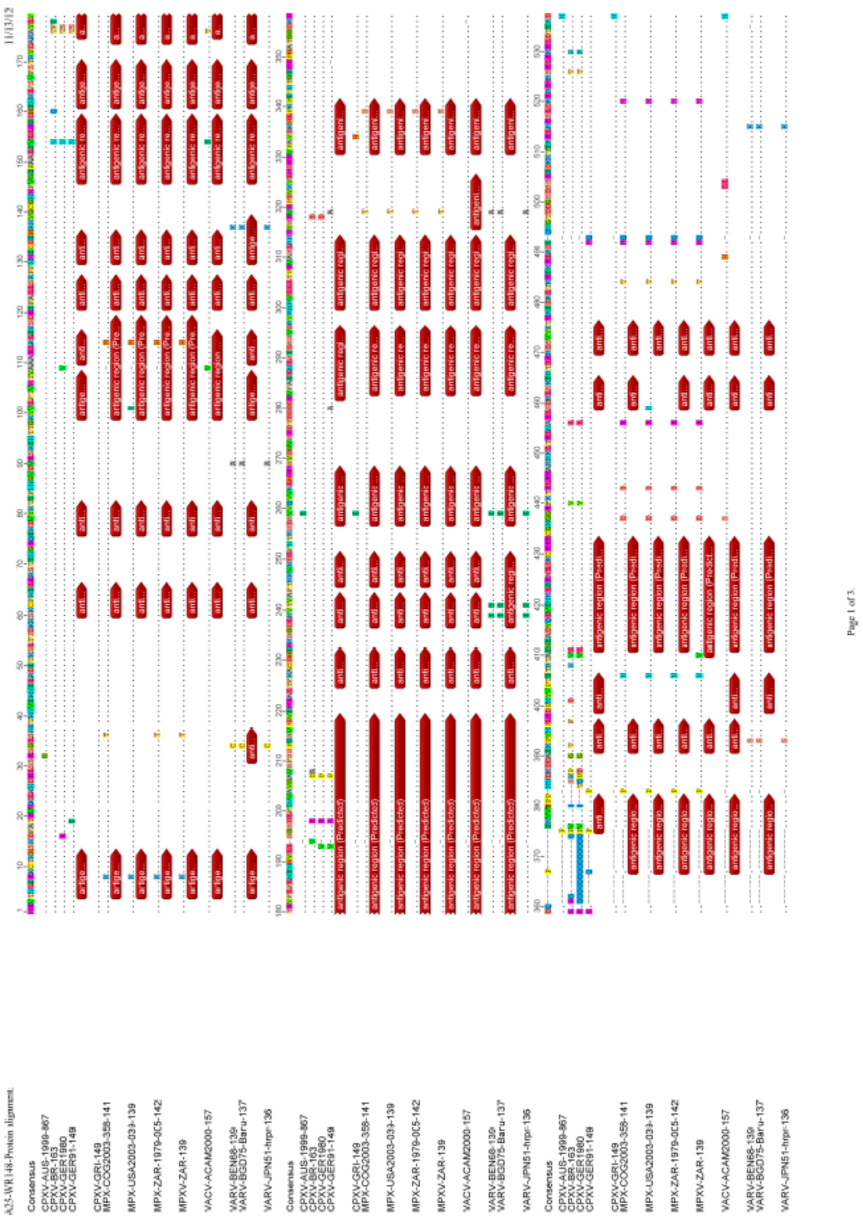

Figure S6. Sequence alignment for protein A25. Page 1 of 3.

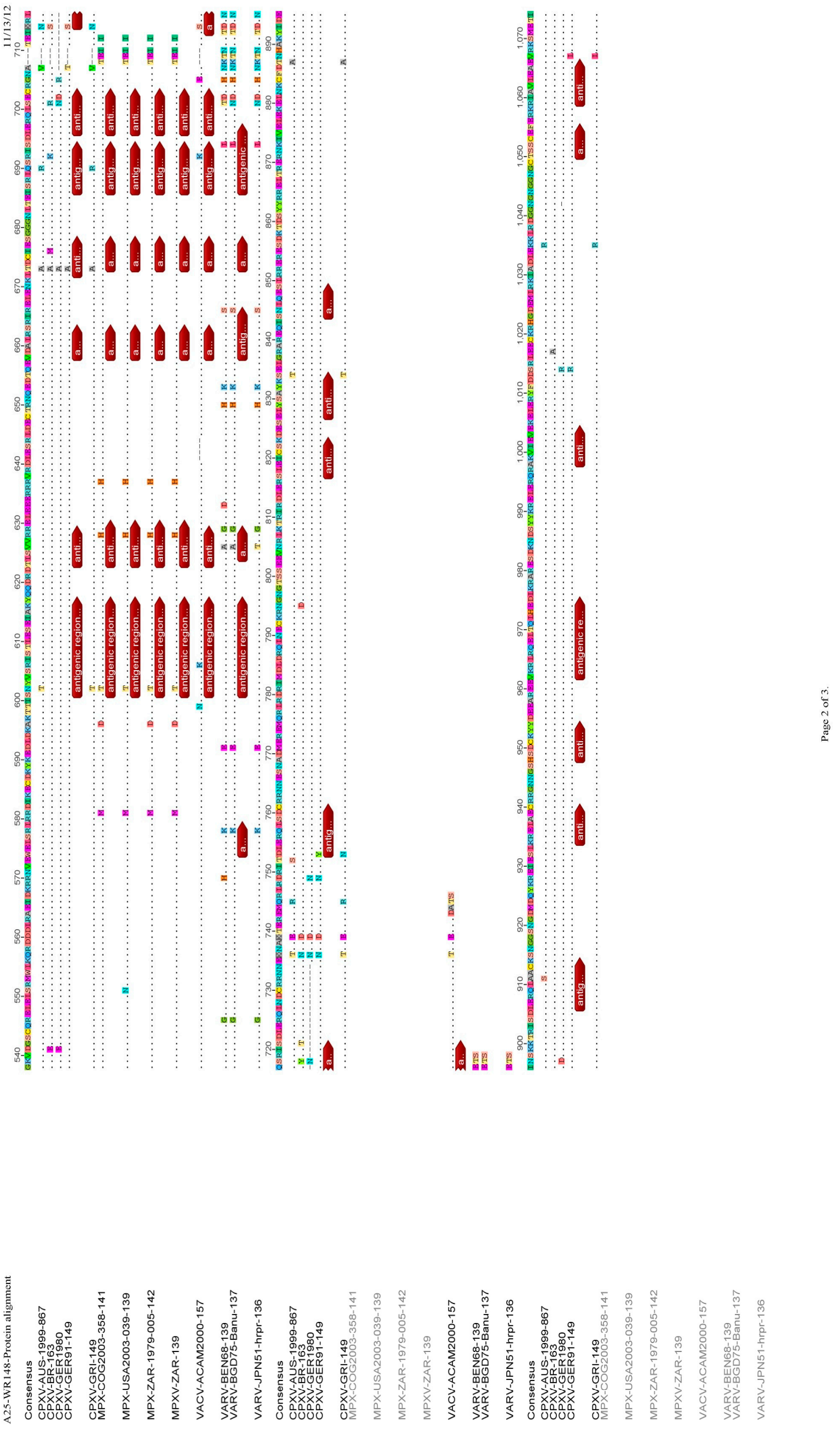

Figure S6. Sequence alignment for protein A25. Page 2 of 3.

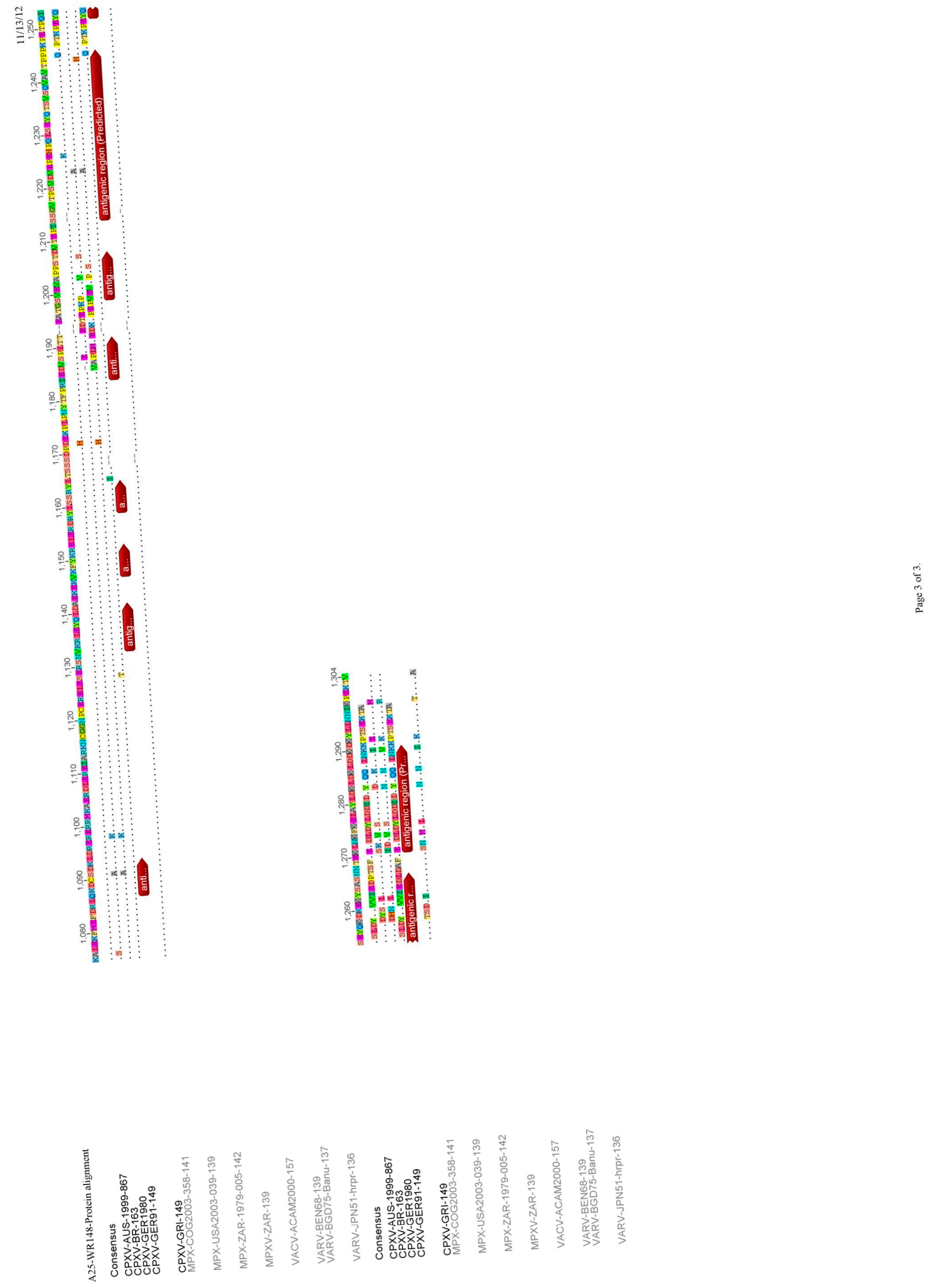

Figure S6. Sequence alignment for protein A25. Page 3 of 3.

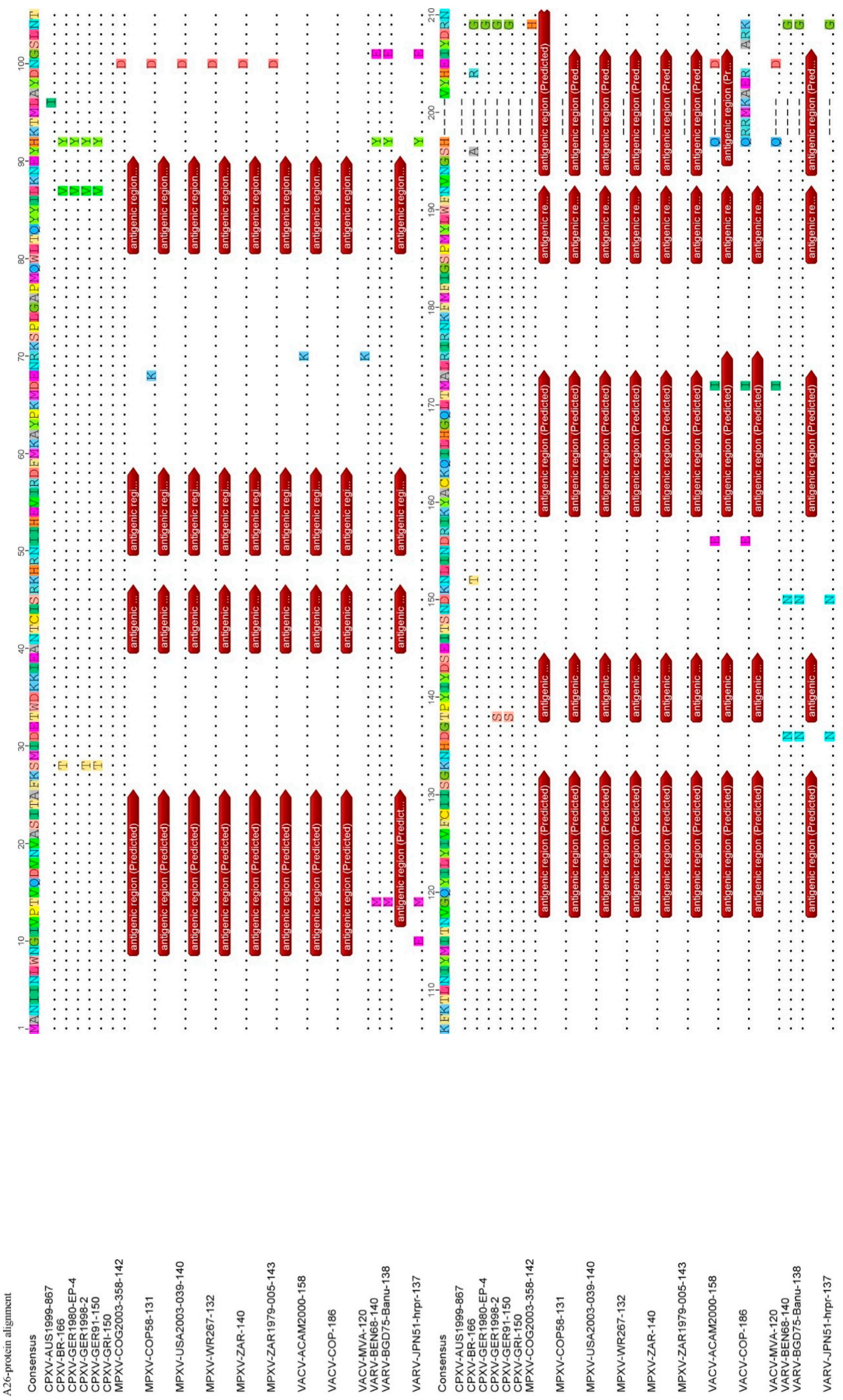

Figure S7. Sequence alignment for protein A26. Page 1 of 3.

Page 1 of 3.

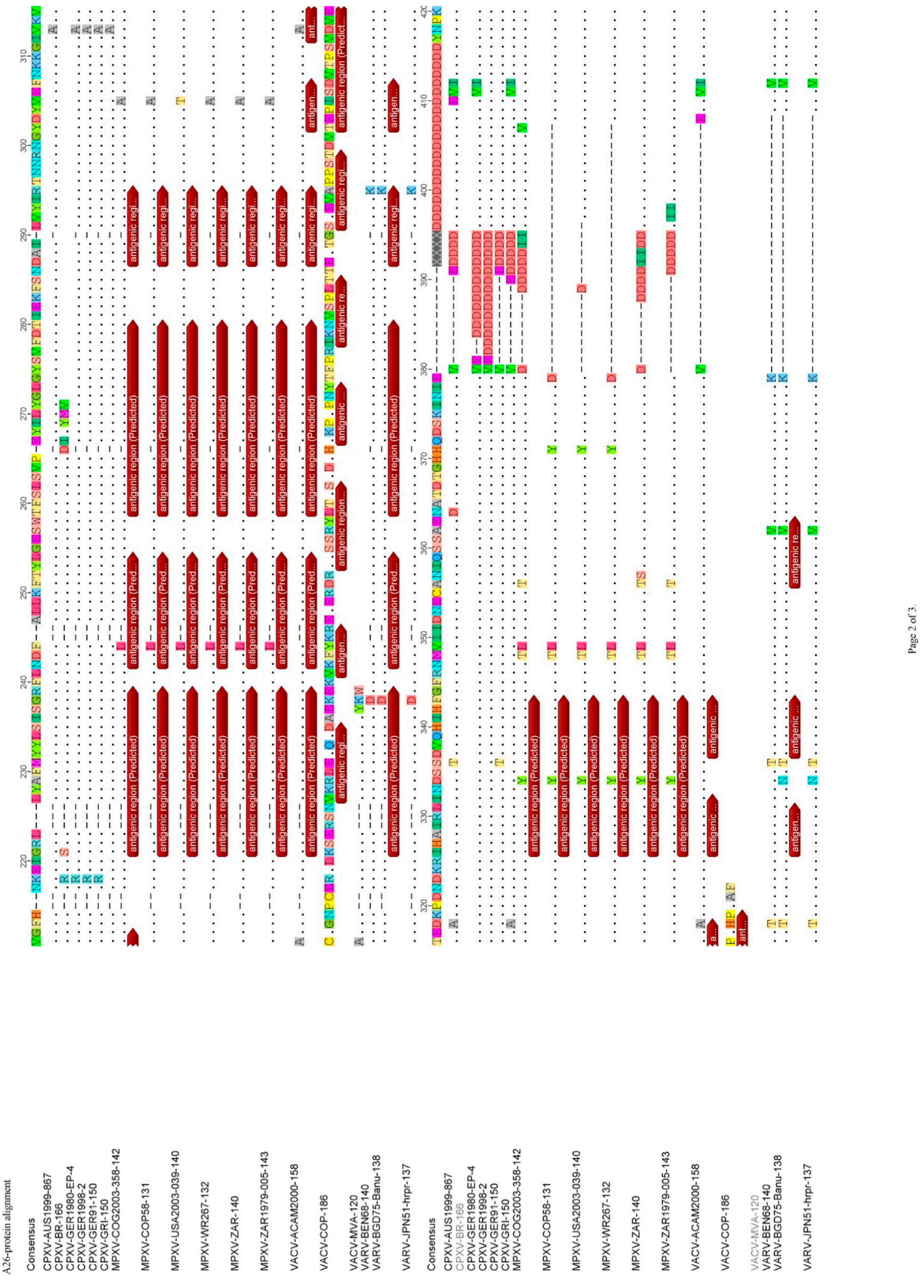

Figure S7. Sequence alignment for protein A26. Page 2 of 3.

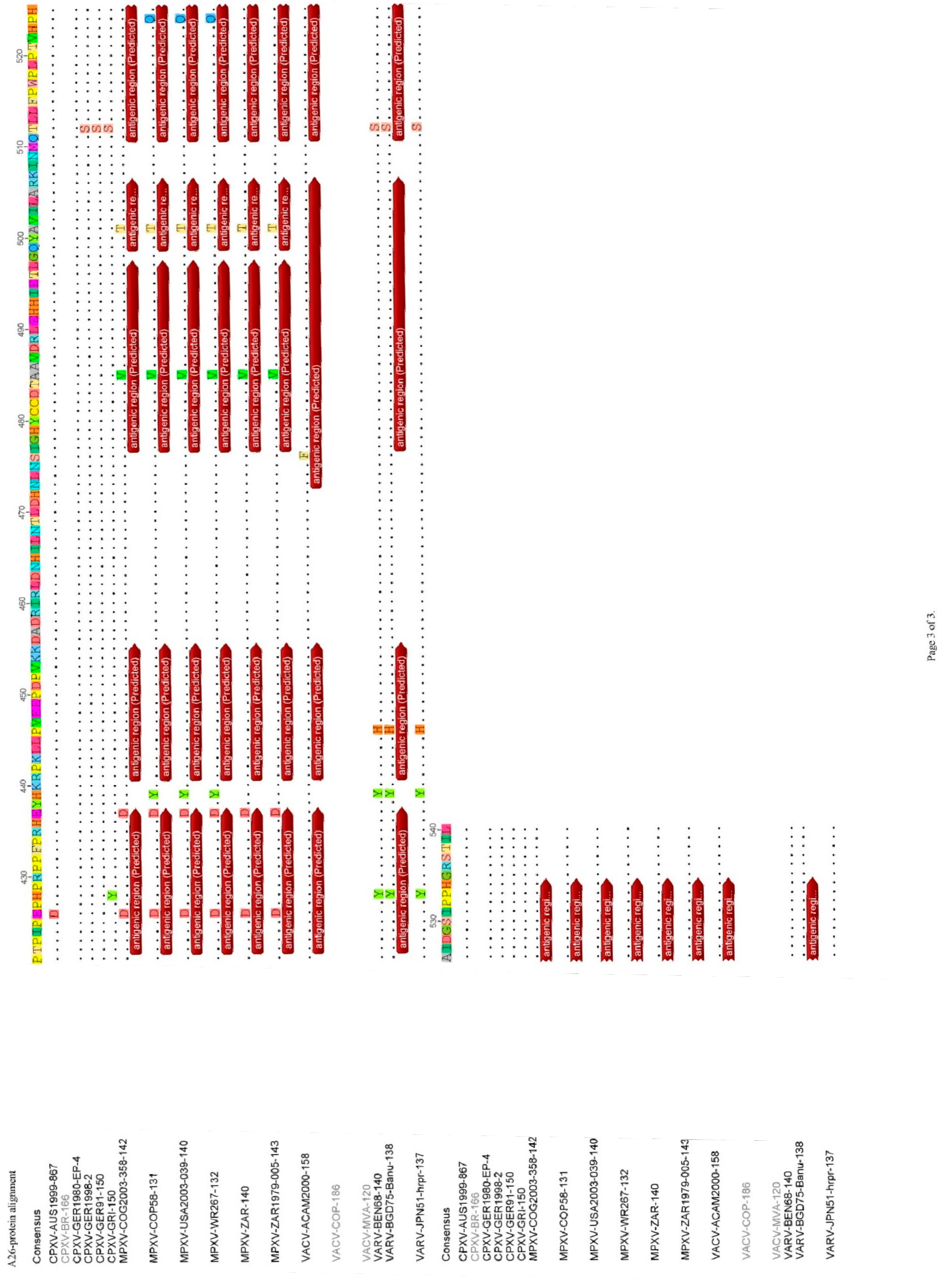

Figure S7. Sequence alignment for protein A26. Page 3 of 3.

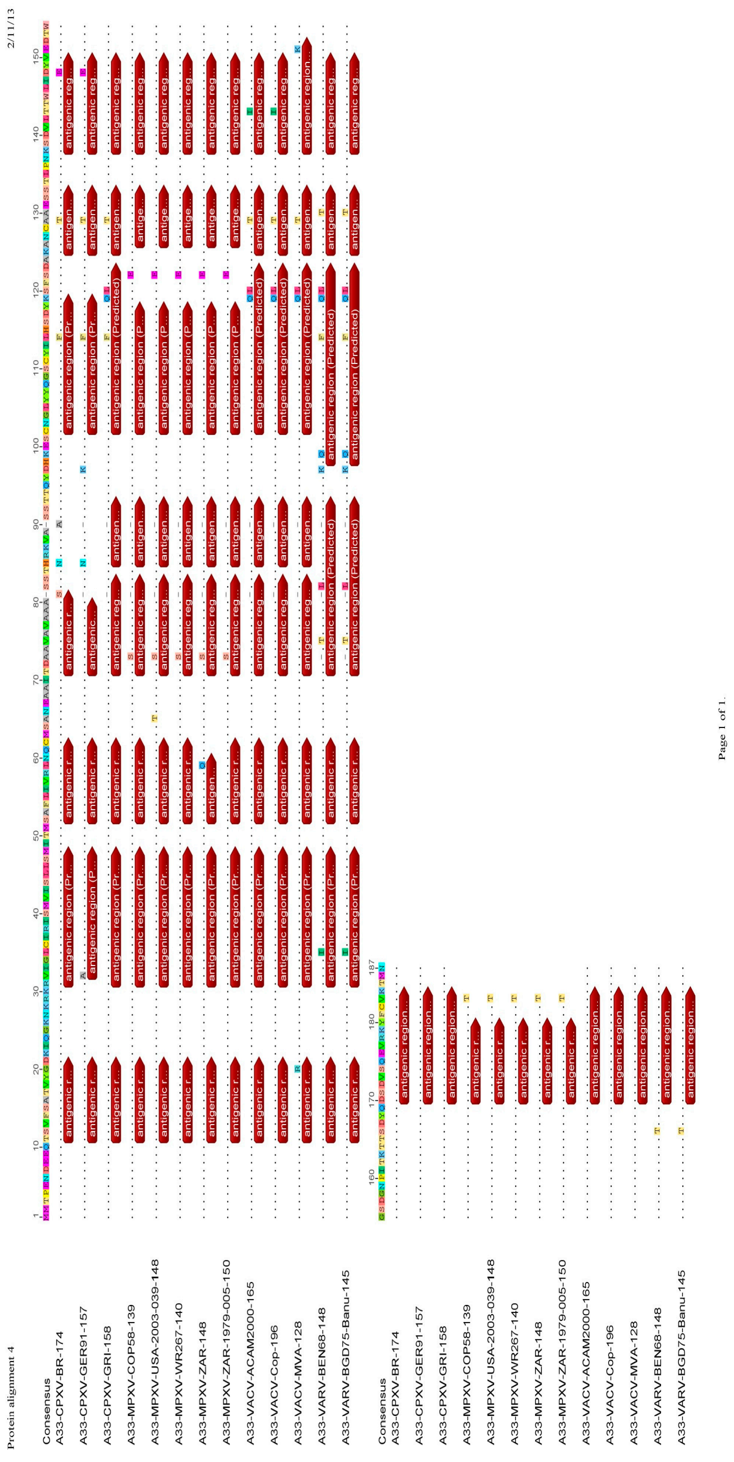

Figure S8. Sequence alignment for protein A33.

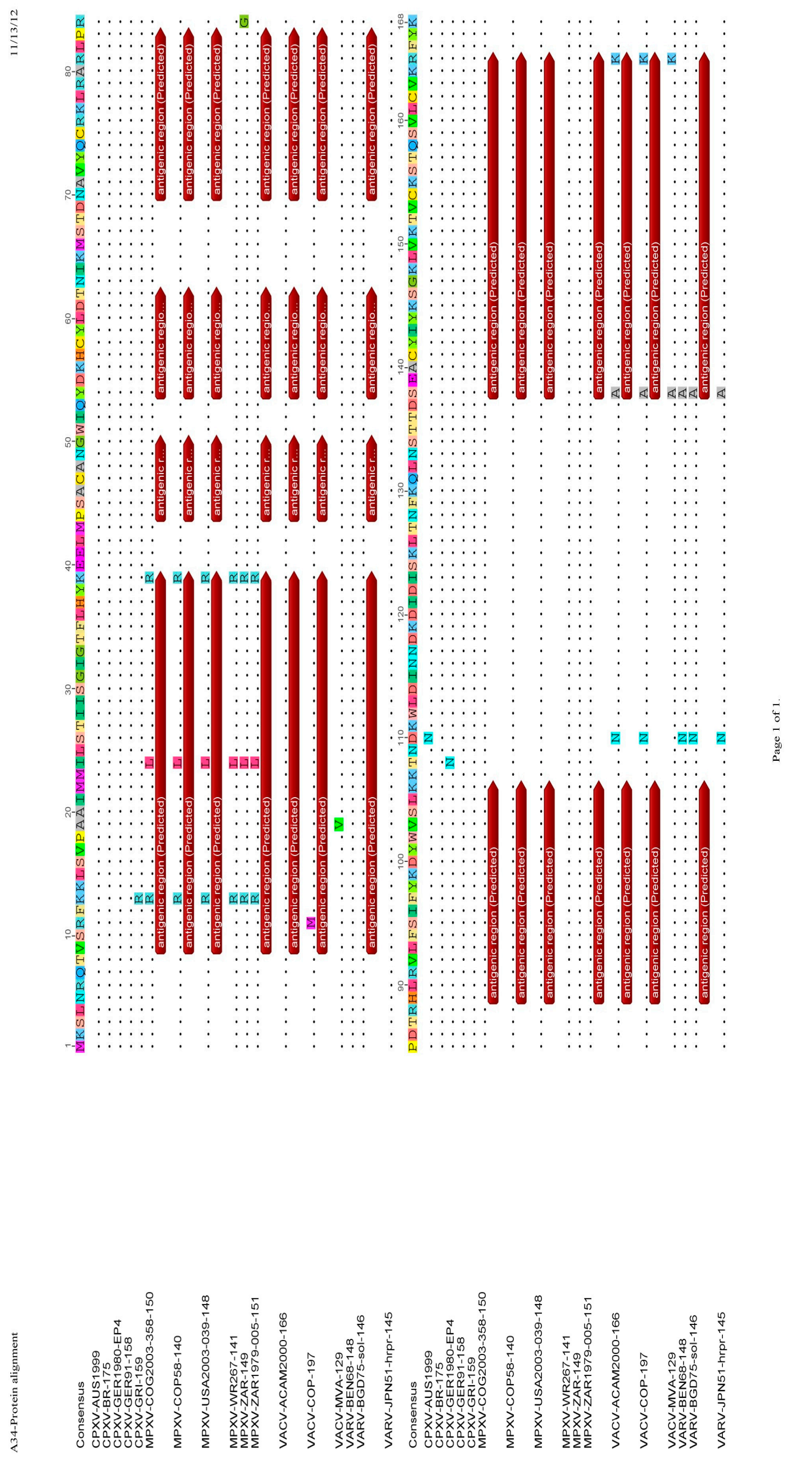

Figure S9. Sequence alignment for protein A34.

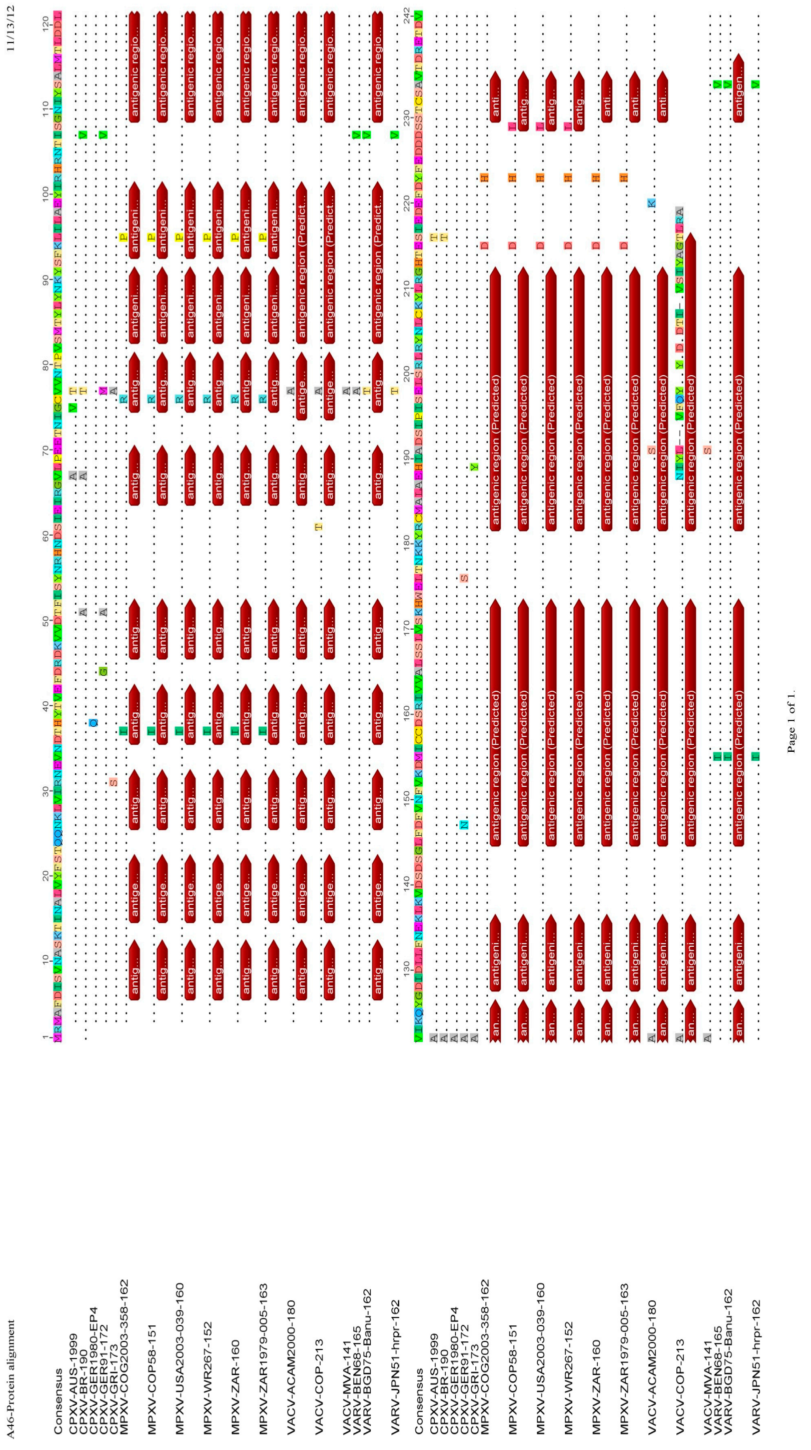

Figure S10. Sequence alignment for protein A46.

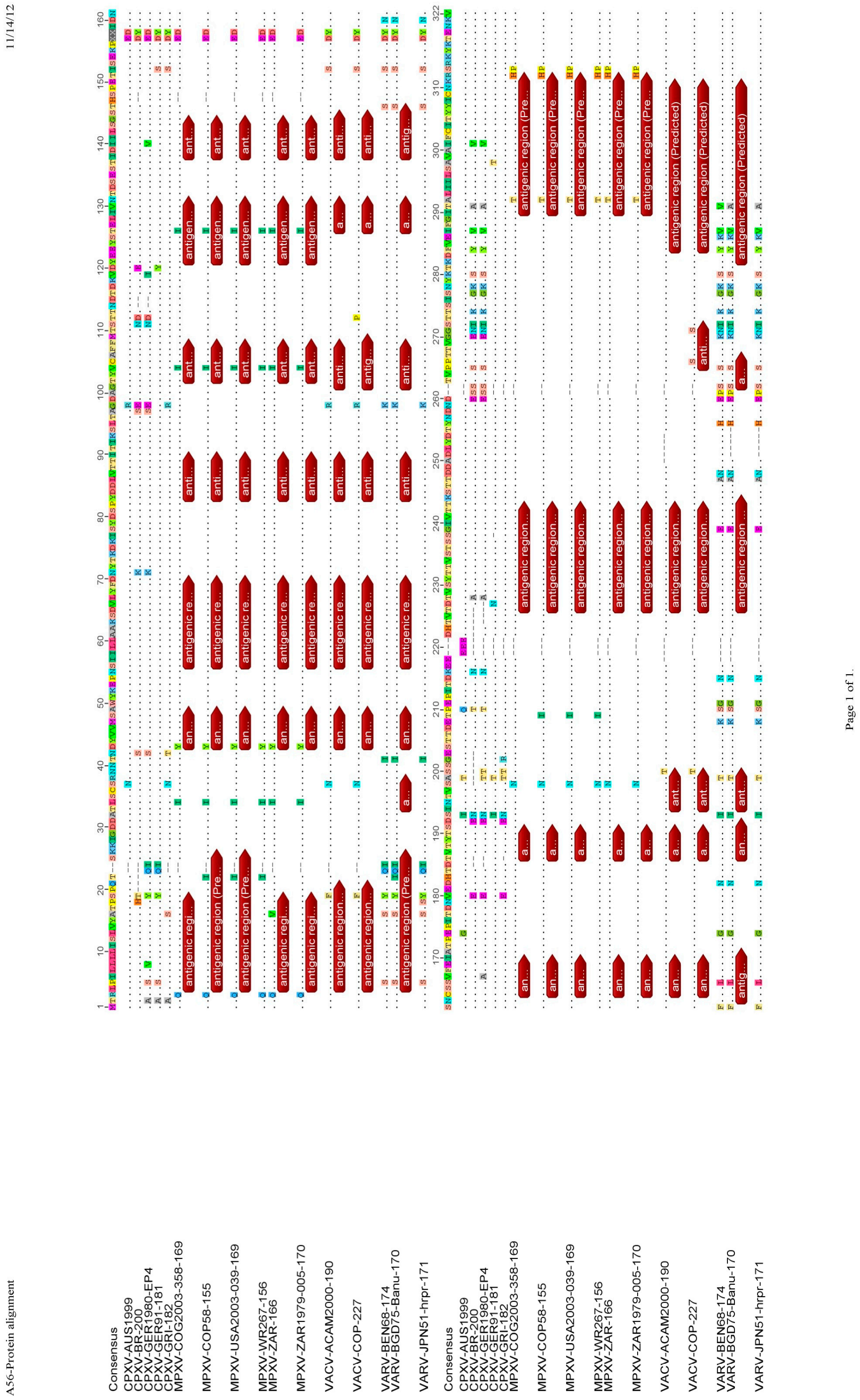

Figure S11. Sequence alignment for protein A56.

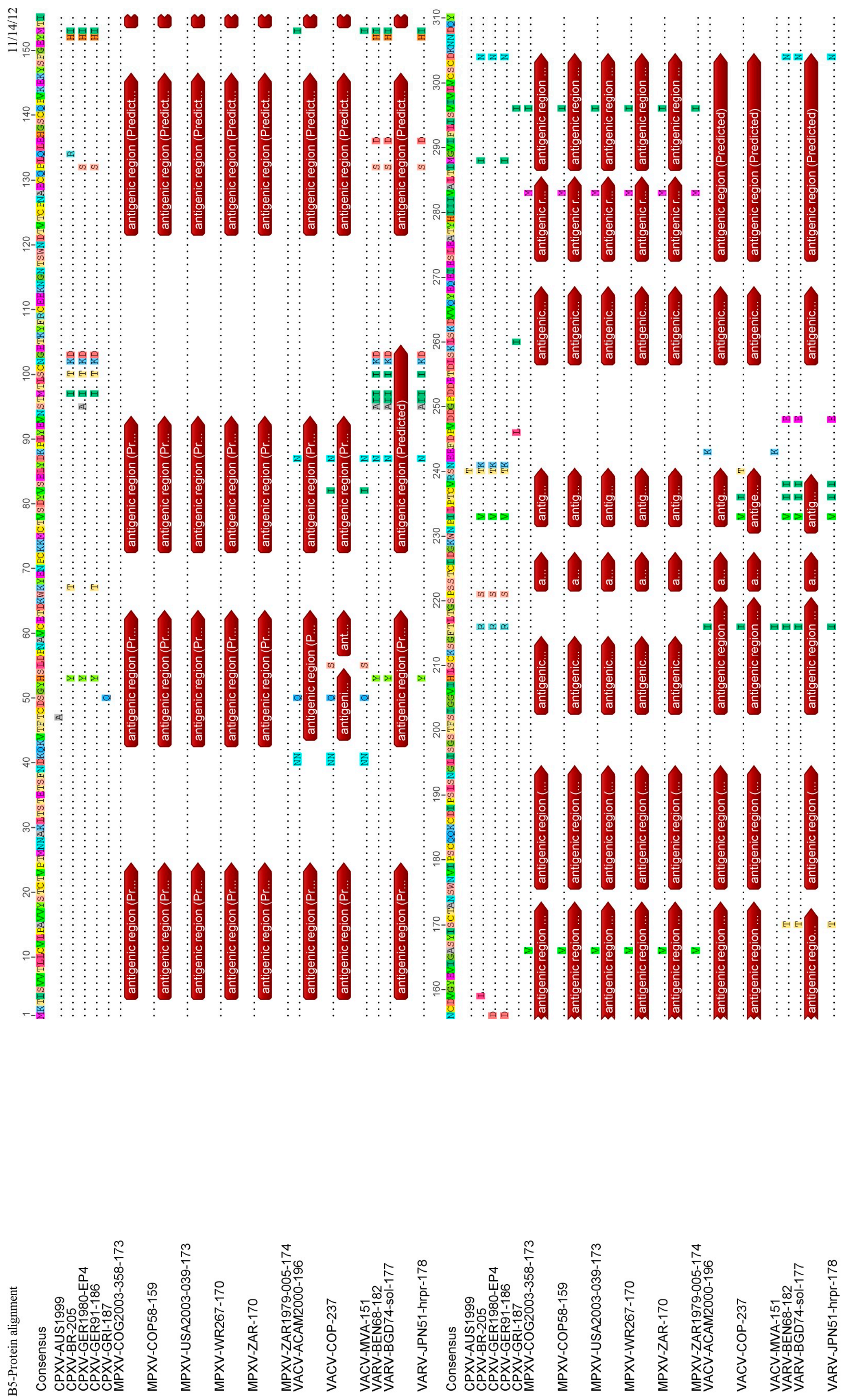

Figure S12. Sequence alignment for protein B5. Page 1 of 2.

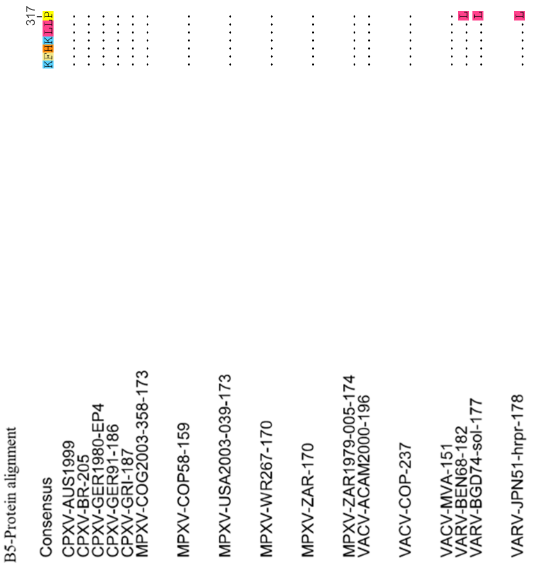

Figure S12. Sequence alignment for protein B5. Page 2 of 2.

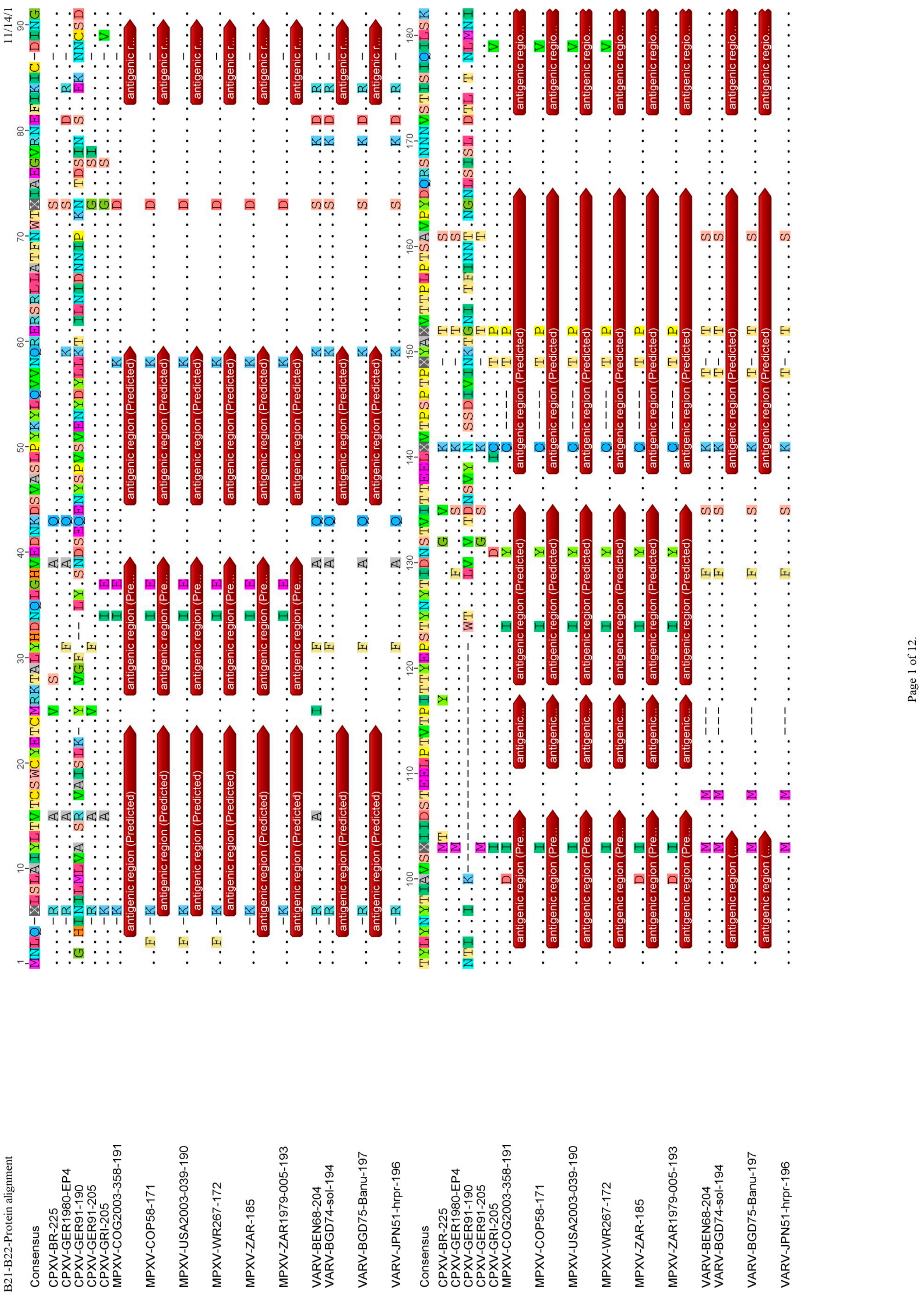

Figure S13. Sequence alignment for protein B21-B22. Page 1 of 12.

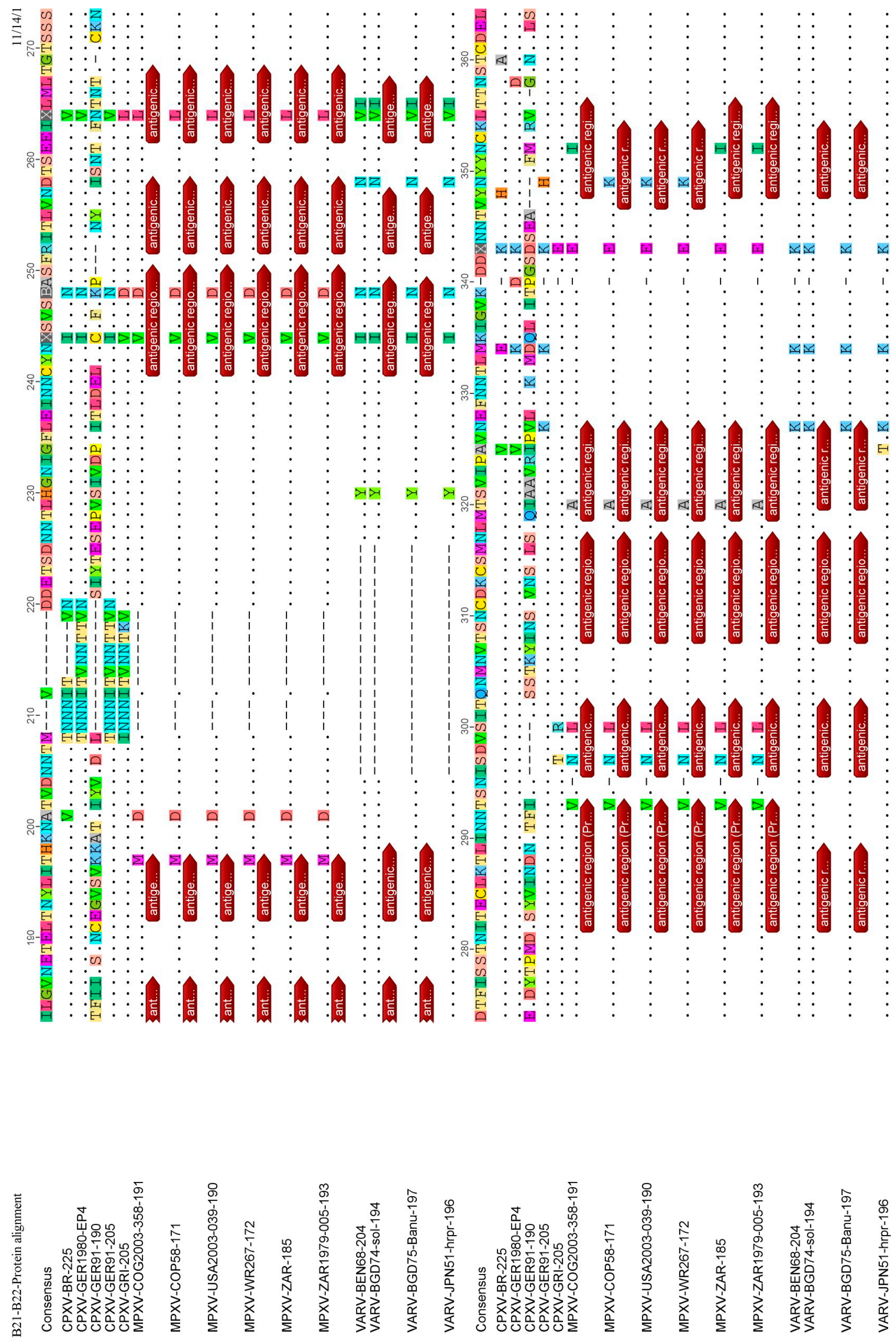

Figure S13. Sequence alignment for protein B21-B22. Page 2 of 12.

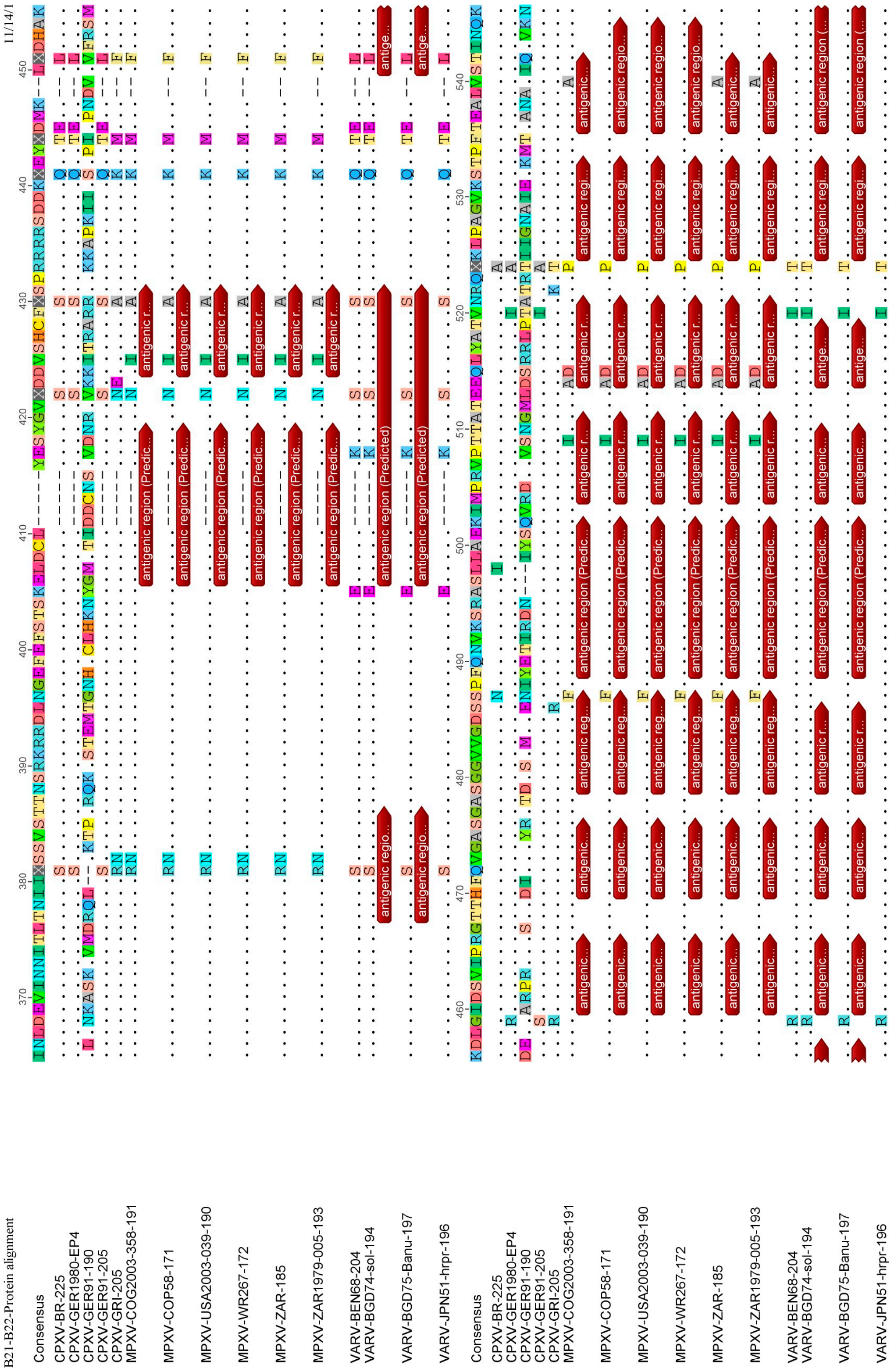

Figure S13. Sequence alignment for protein B21-B22. Page 3 of 12.

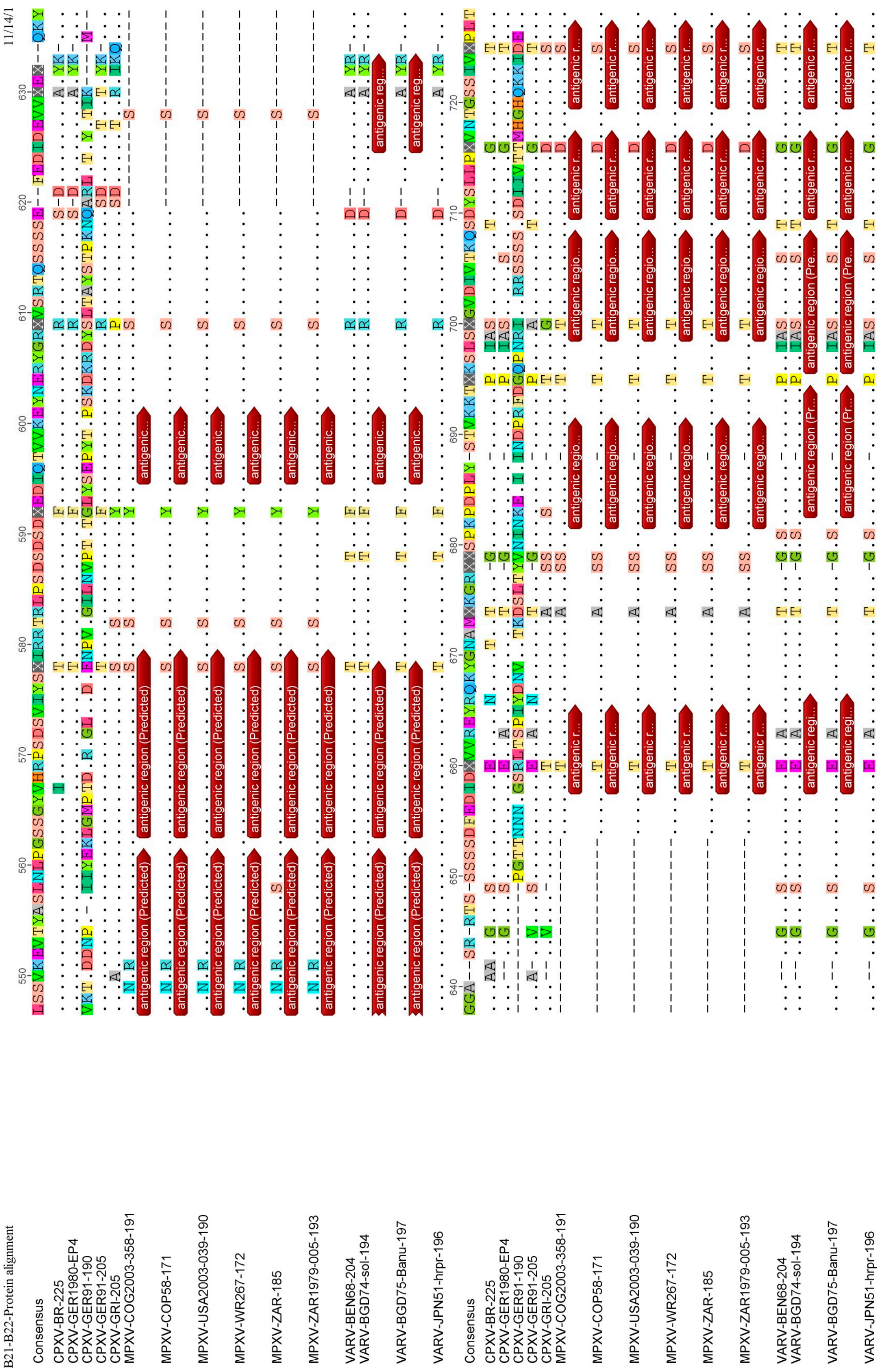

Figure S13. Sequence alignment for protein B21-B22. Page 4 of 12.

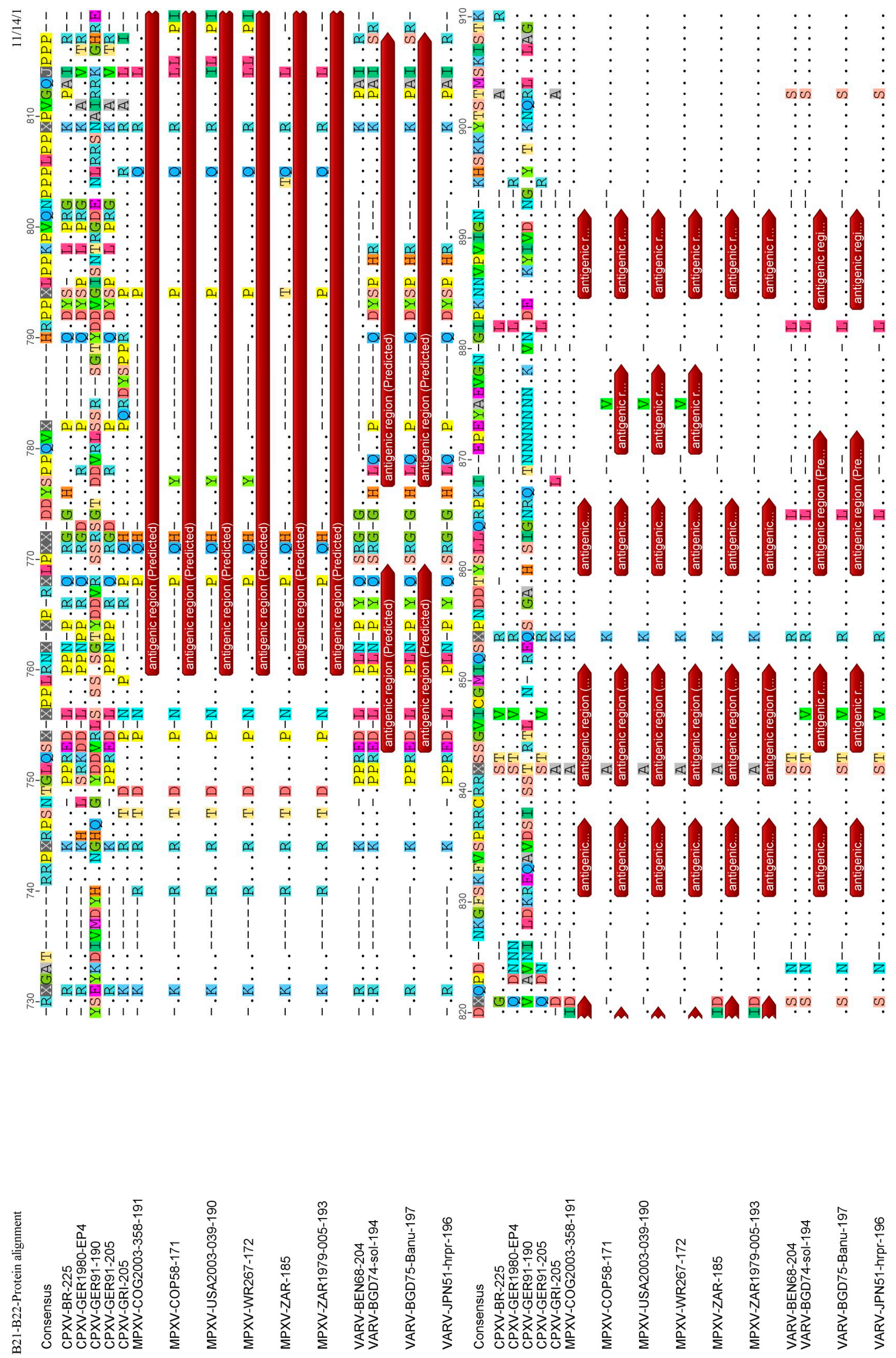

Figure S13. Sequence alignment for protein B21-B22. Page 5 of 12.

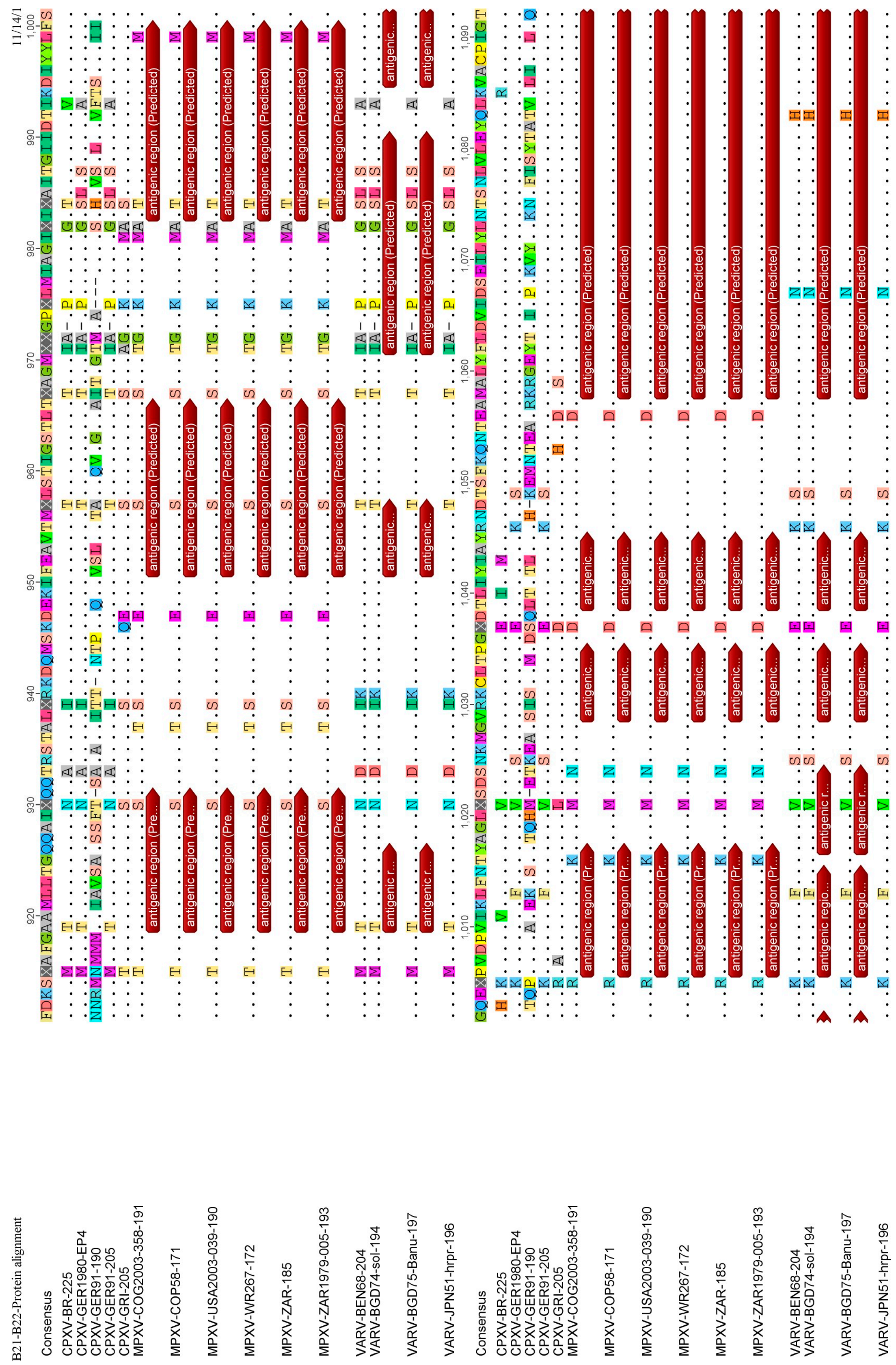

Figure S13. Sequence alignment for protein B21-B22. Page 6 of 12.

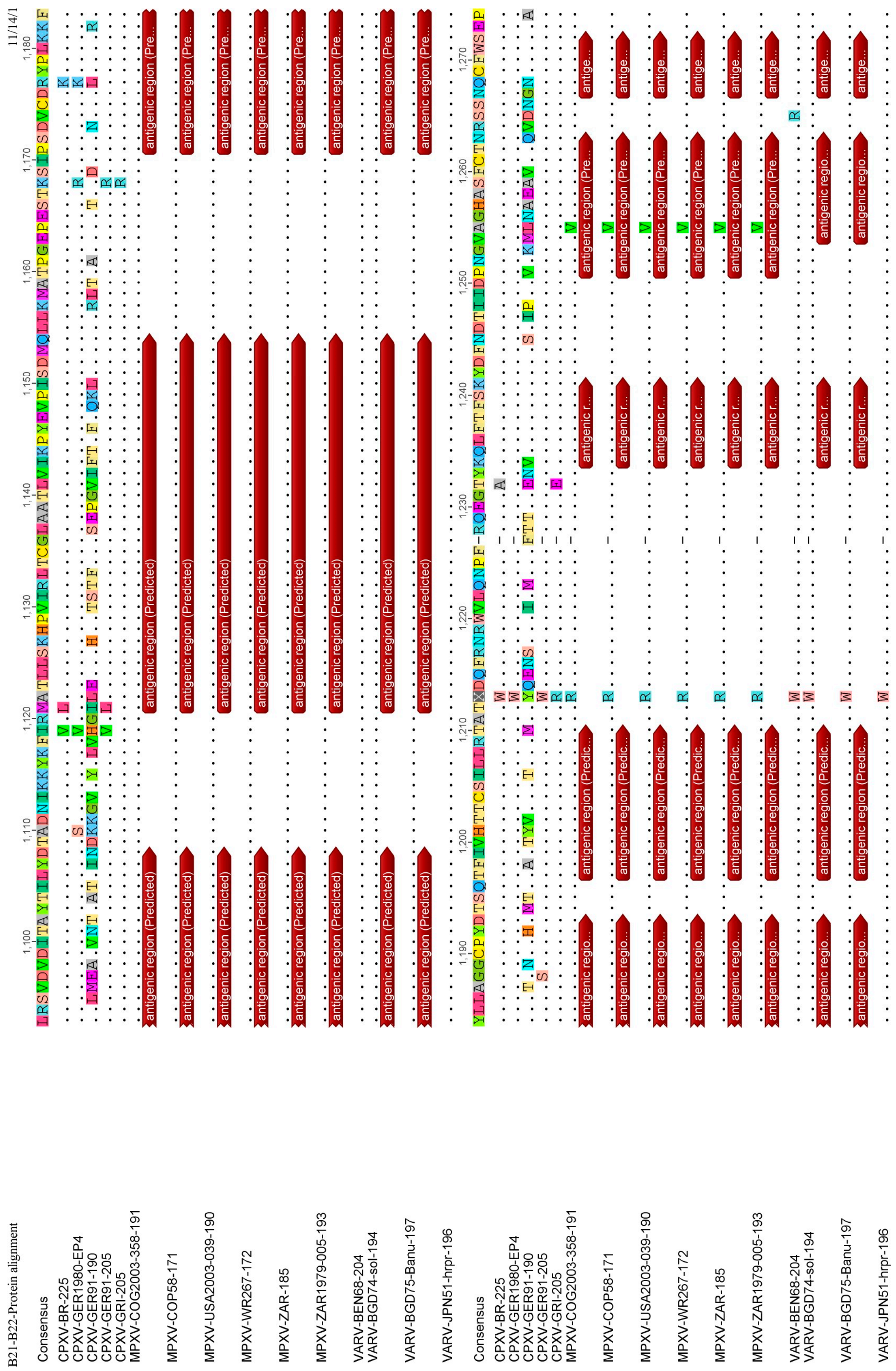

Figure S13. Sequence alignment for protein B21-B22. Page 7 of 12.

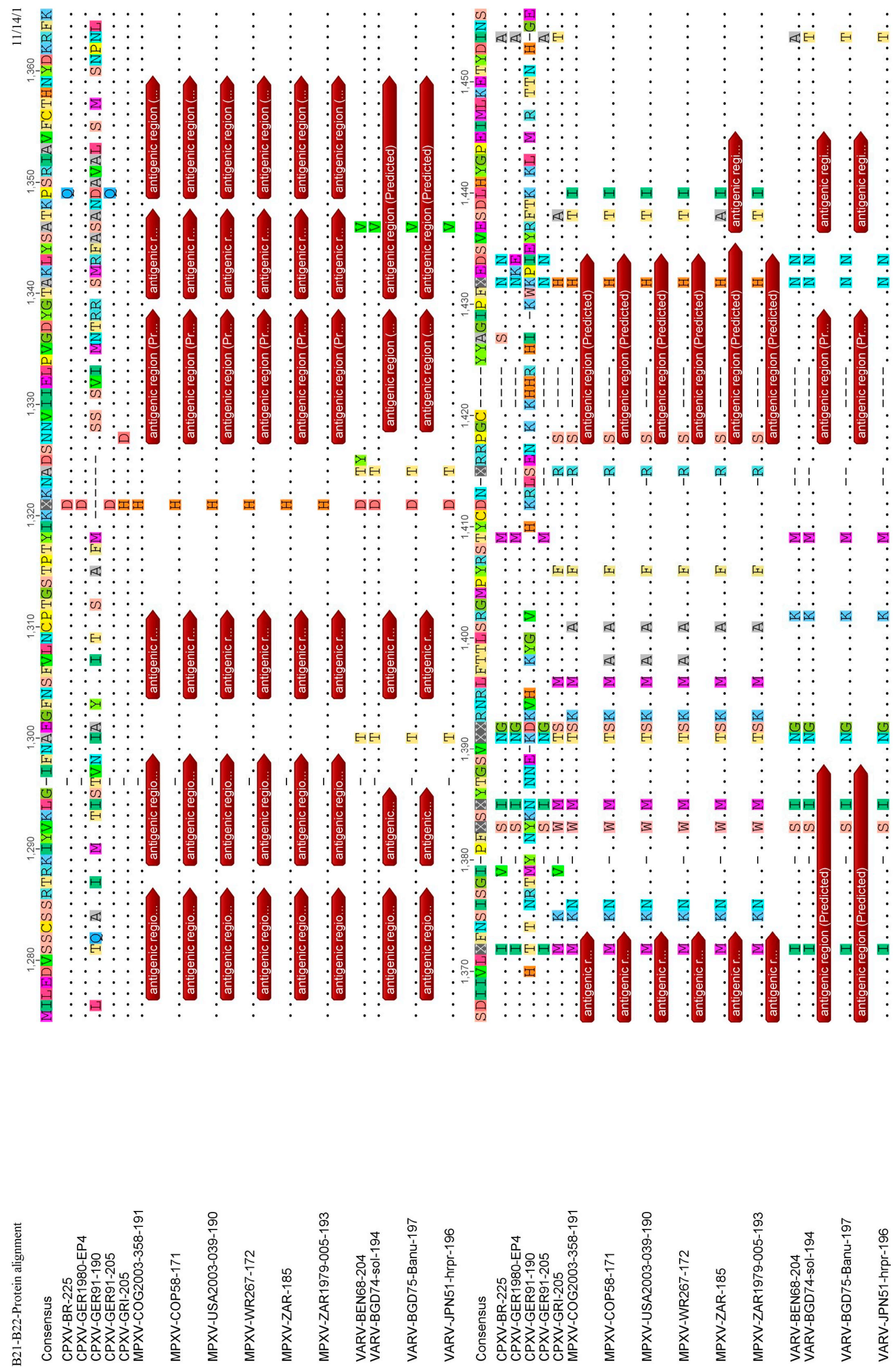

Figure S13. Sequence alignment for protein B21-B22. Page 8 of 12.

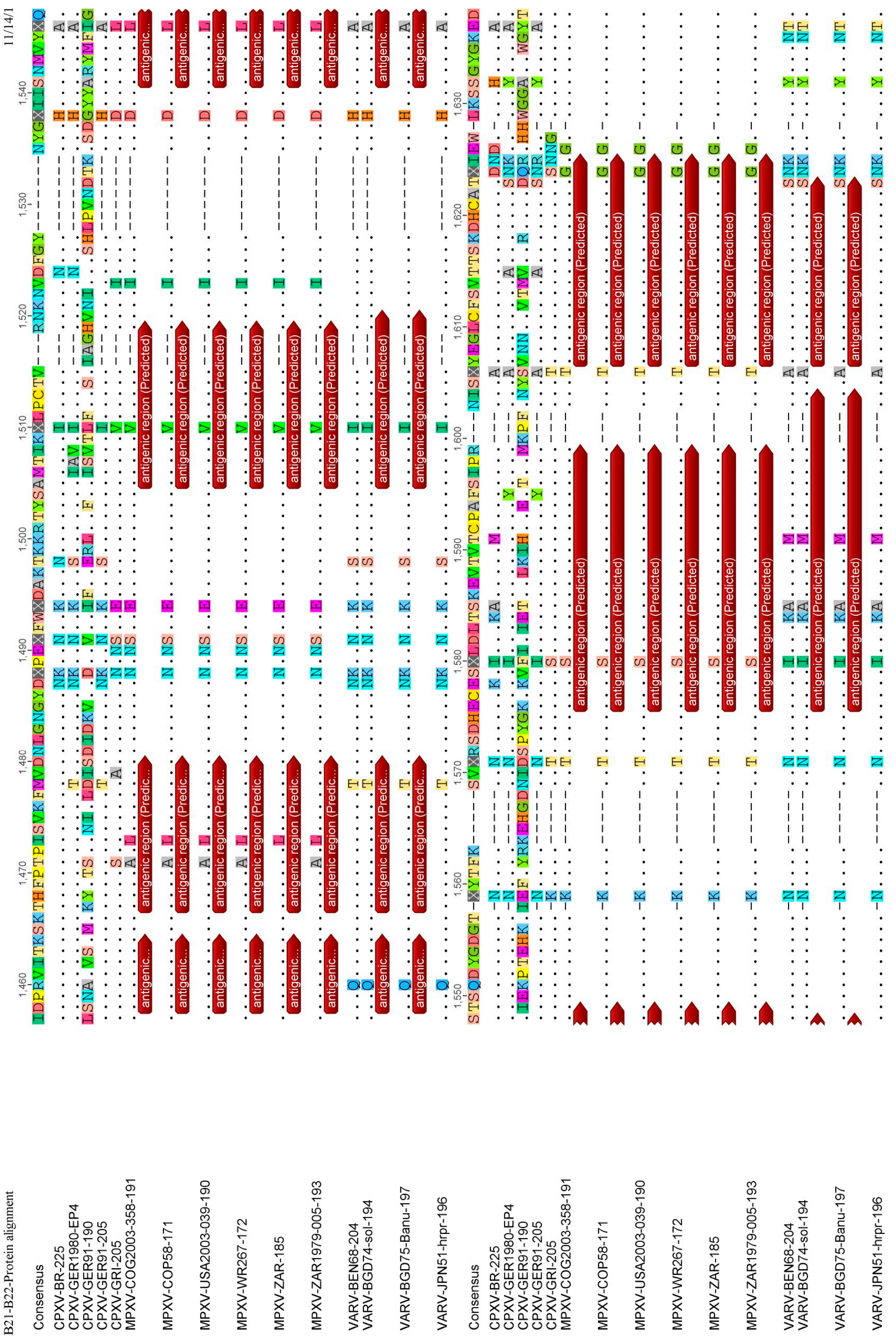

Figure S13. Sequence alignment for protein B21-B22. Page 9 of 12.

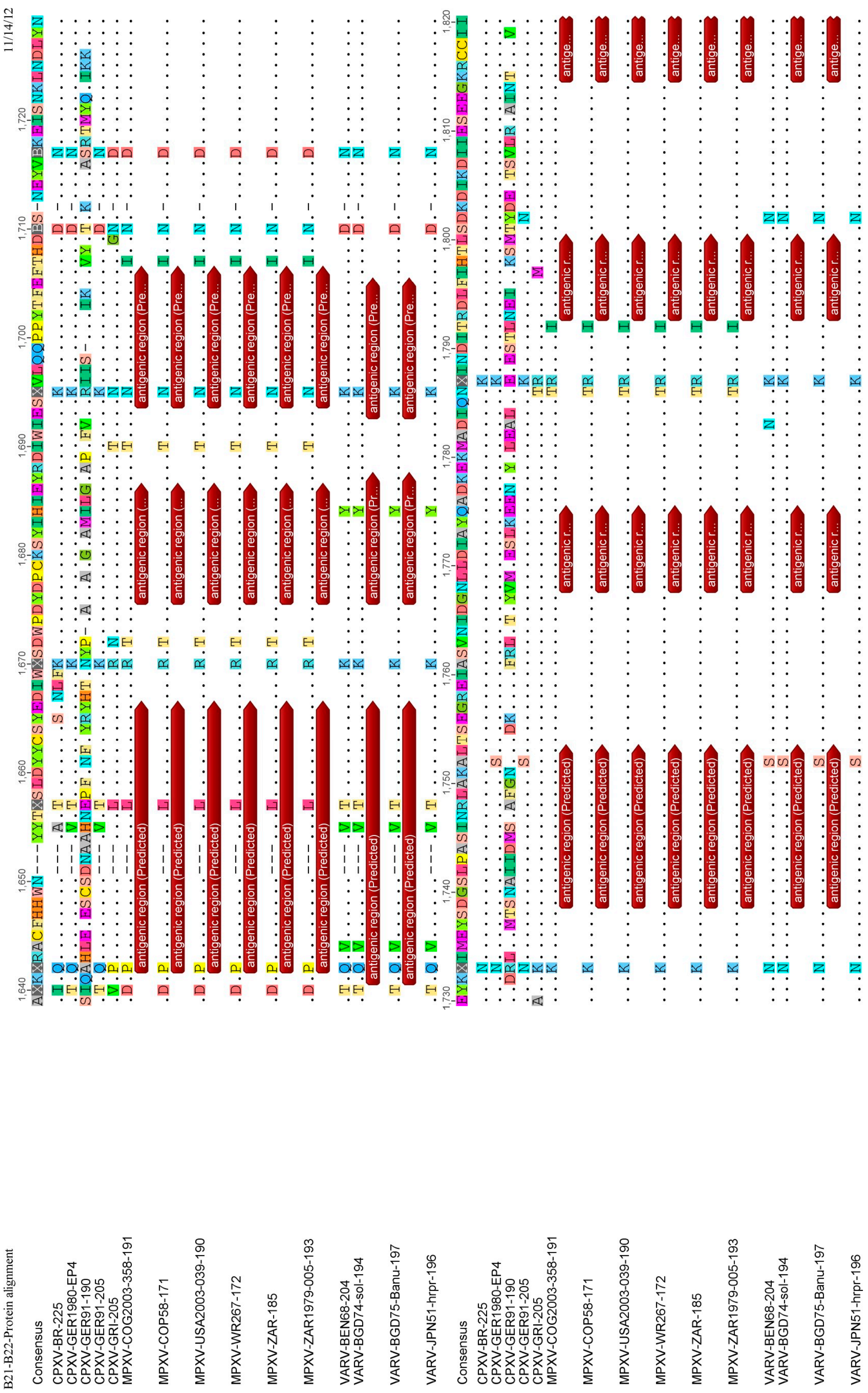

Figure S13. Sequence alignment for protein B21-B22. Page 10 of 12.

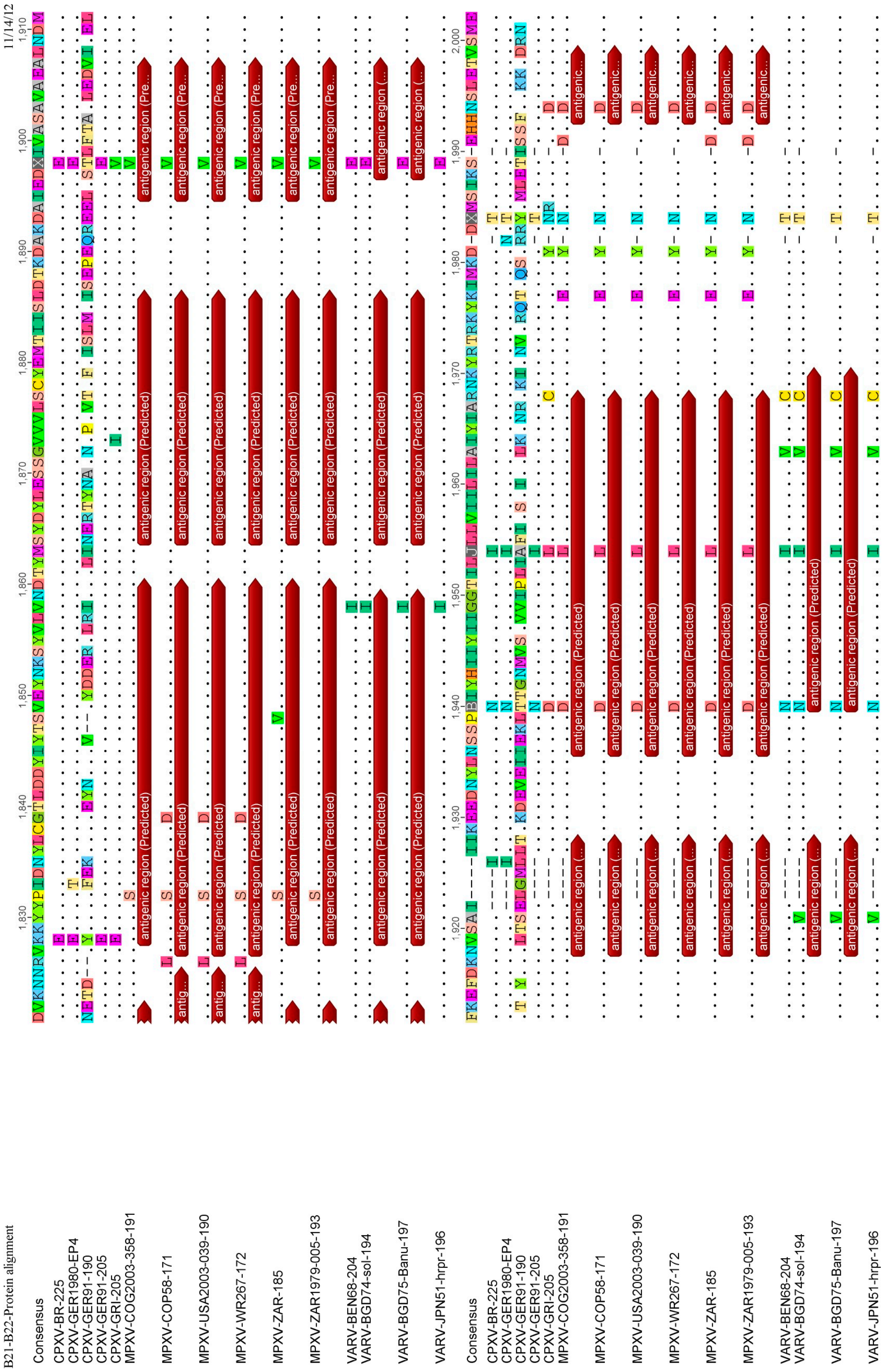

Figure S13. Sequence alignment for protein B21-B22. Page 11 of 12.

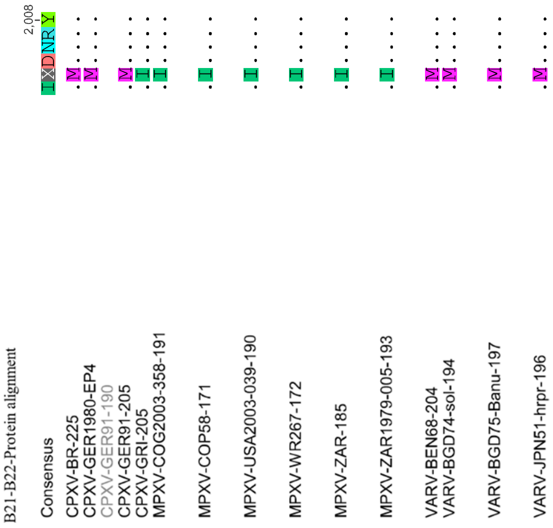

Figure S13. Sequence alignment for protein B21-B22. Page 12 of 12.

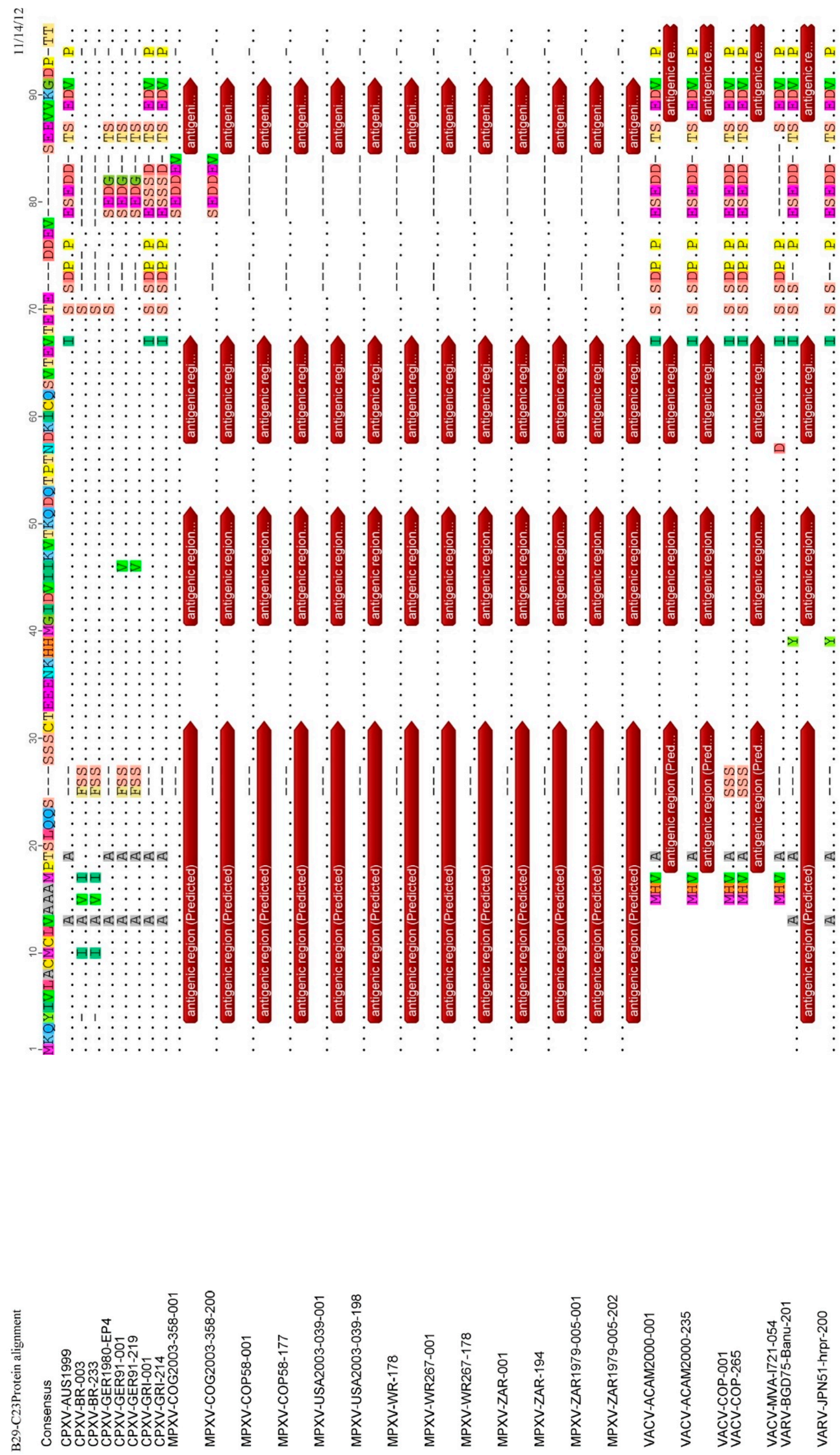

Figure S14. Sequence alignment for protein B29-C23. Page 1 of 3.

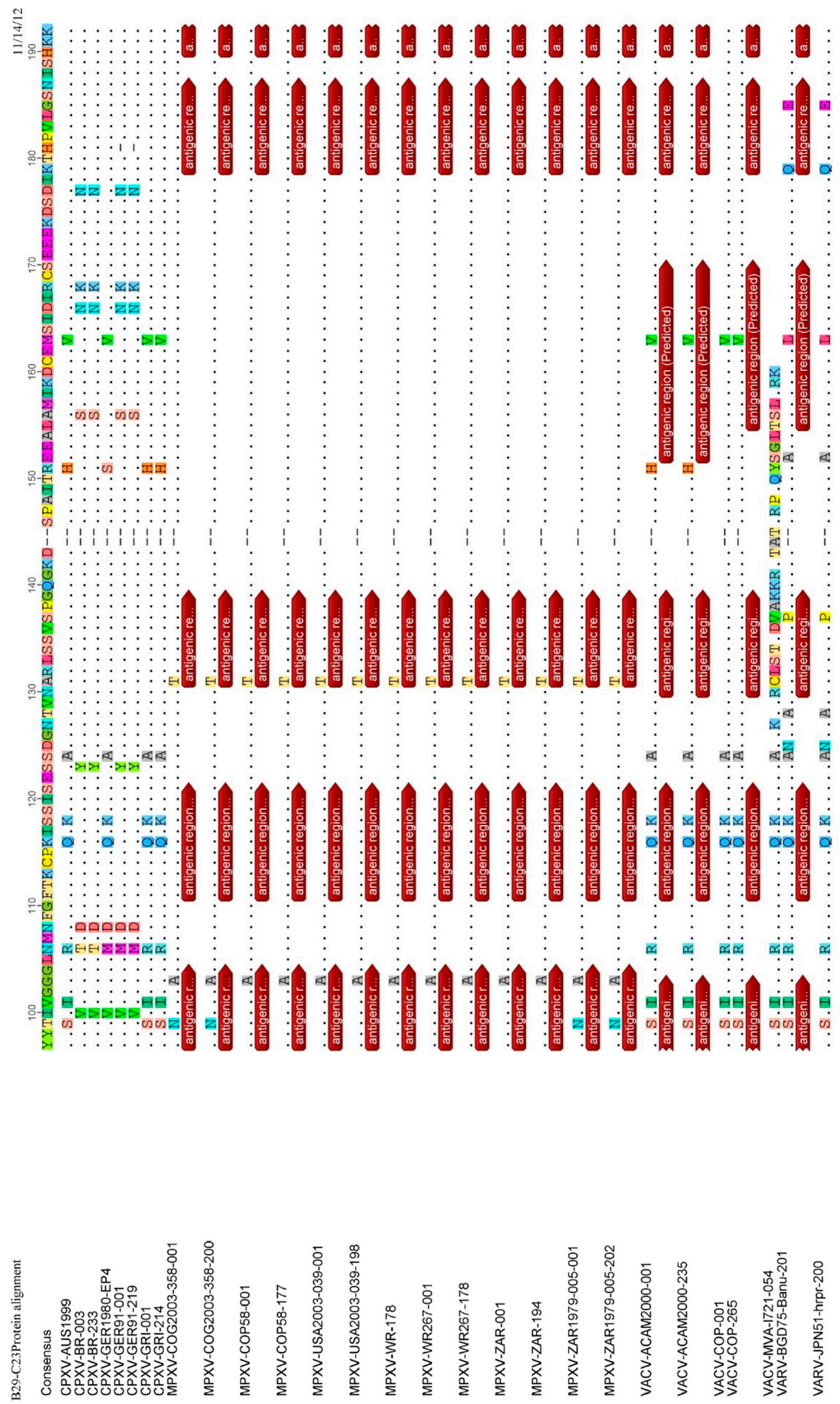

Figure S14. Sequence alignment for protein B29-C23. Page 2 of 3.

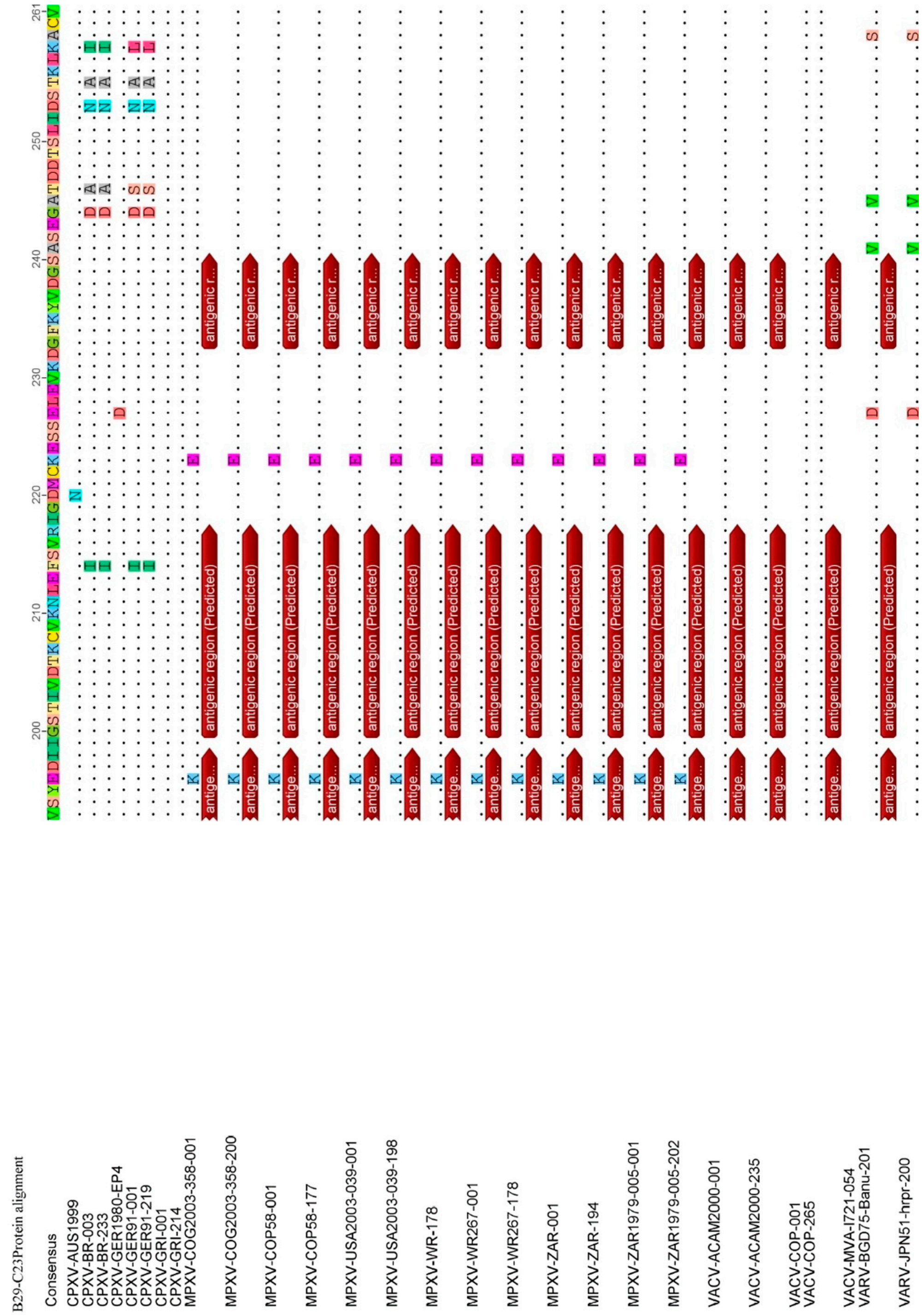

Figure S14. Sequence alignment for protein B29-C23. Page 3 of 3.

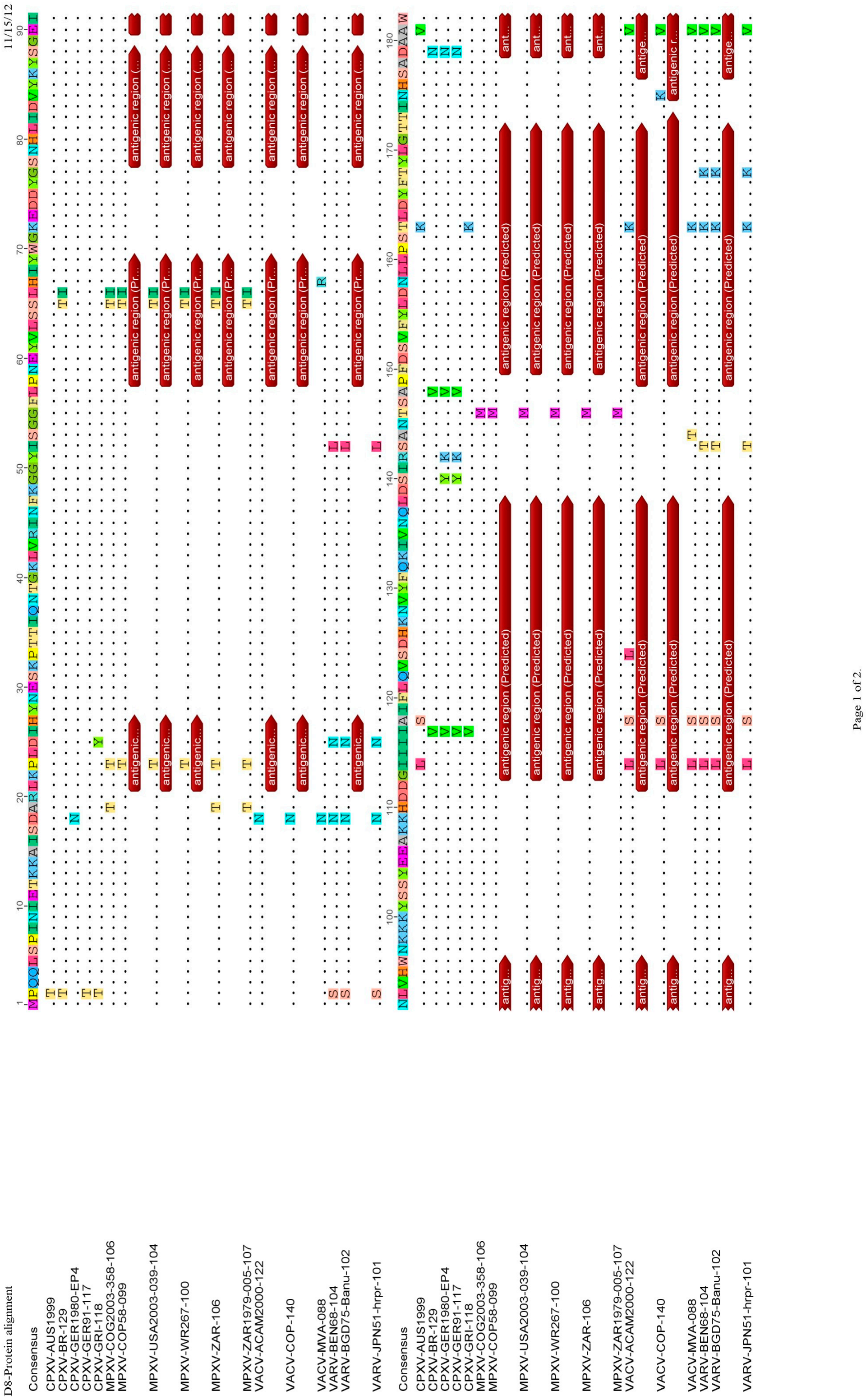

Figure S15. Sequence alignment for protein D8. Page 1 of 2.

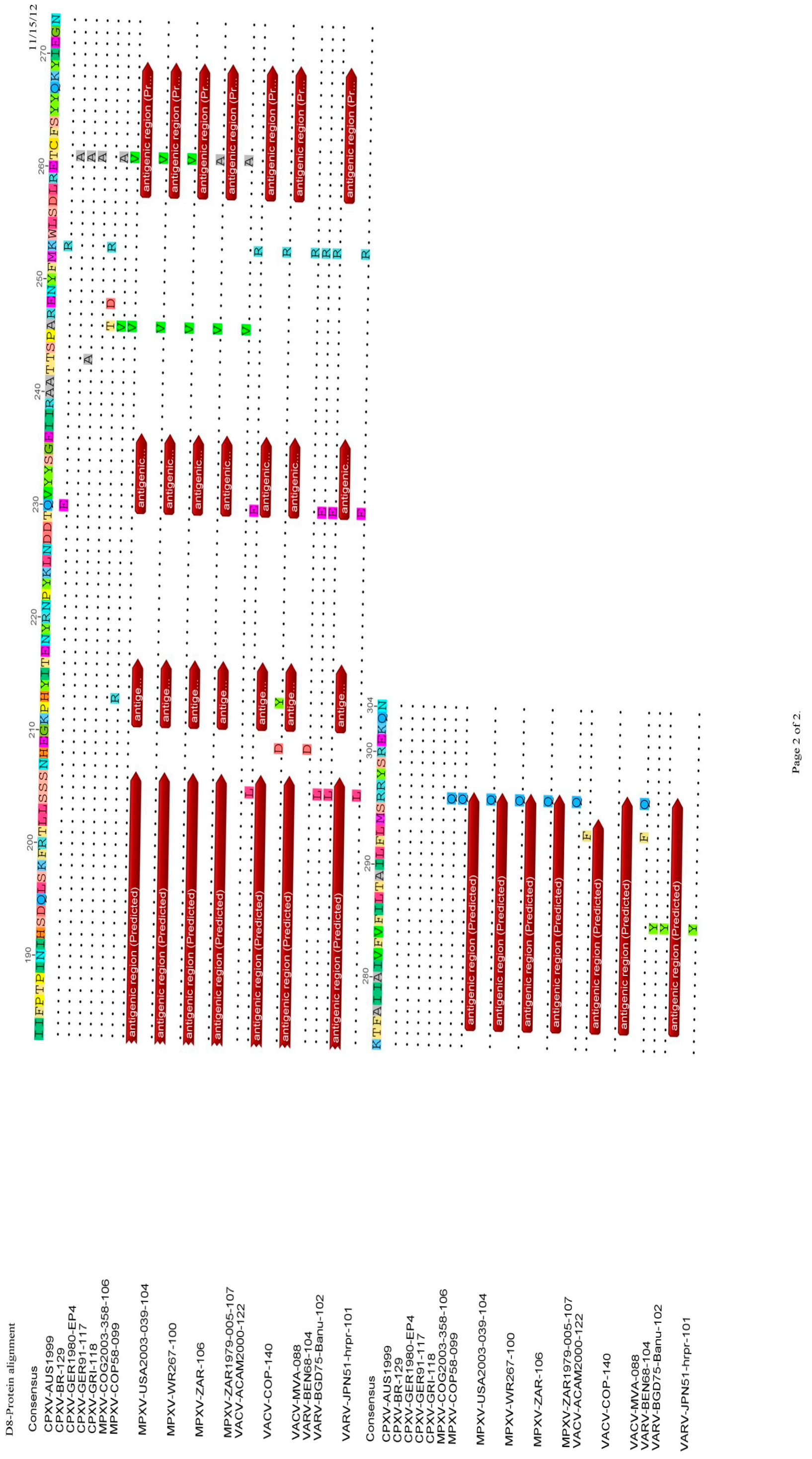

Figure S15. Sequence alignment for protein D8. Page 2 of 2.

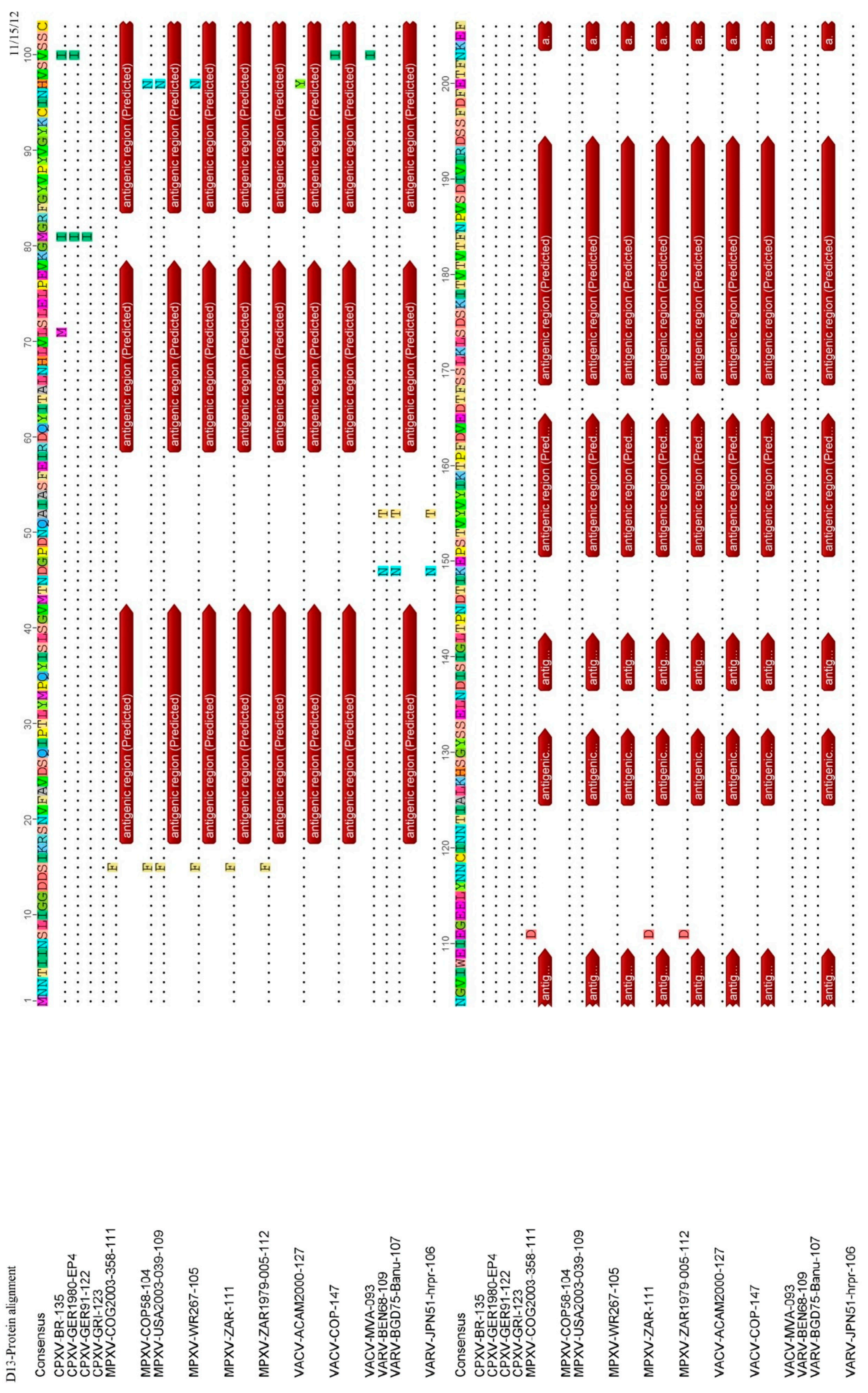

Figure S16. Sequence alignment for protein D13. Page 1 of 3.

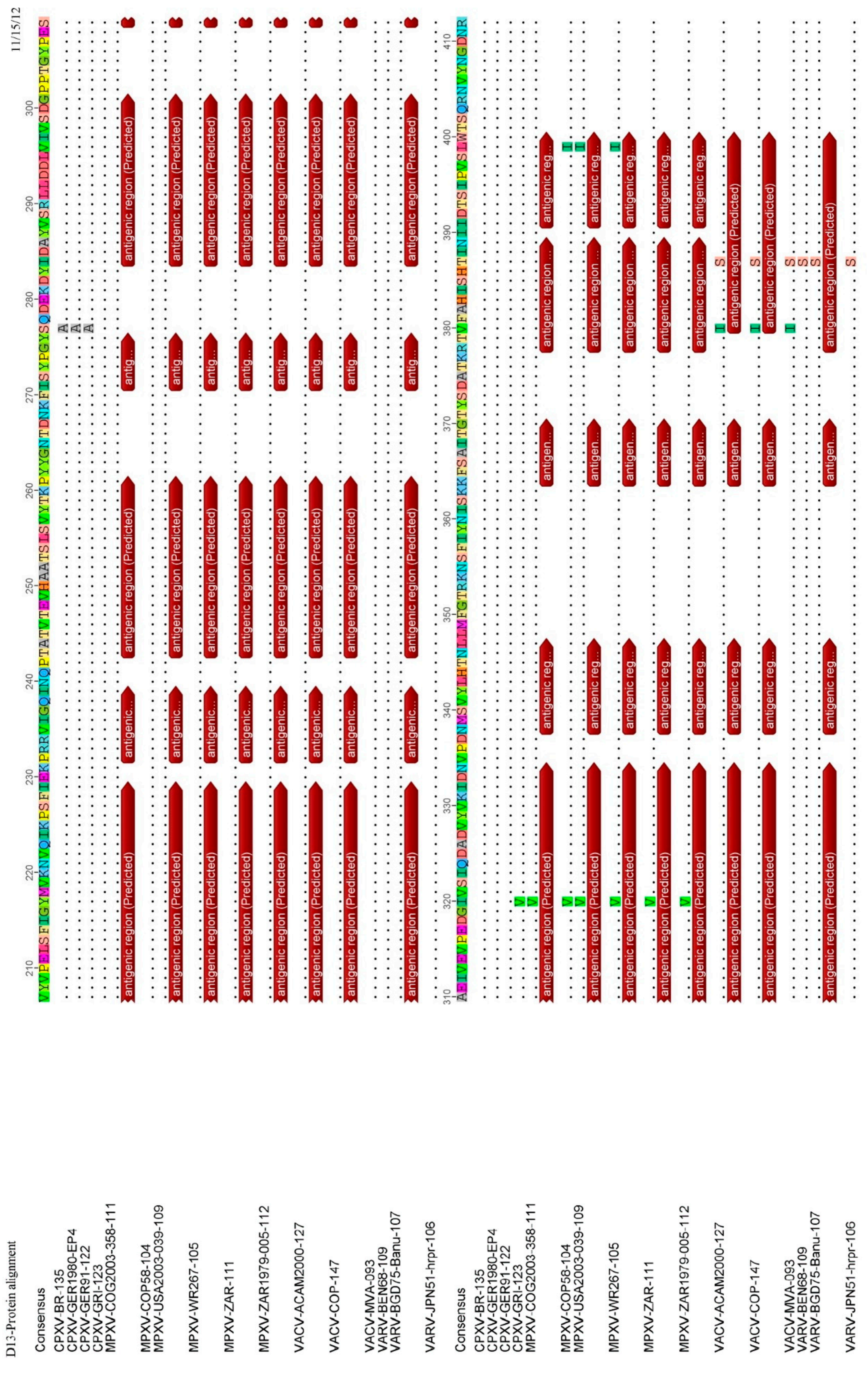

Figure S16. Sequence alignment for protein D13. Page 2 of 3.

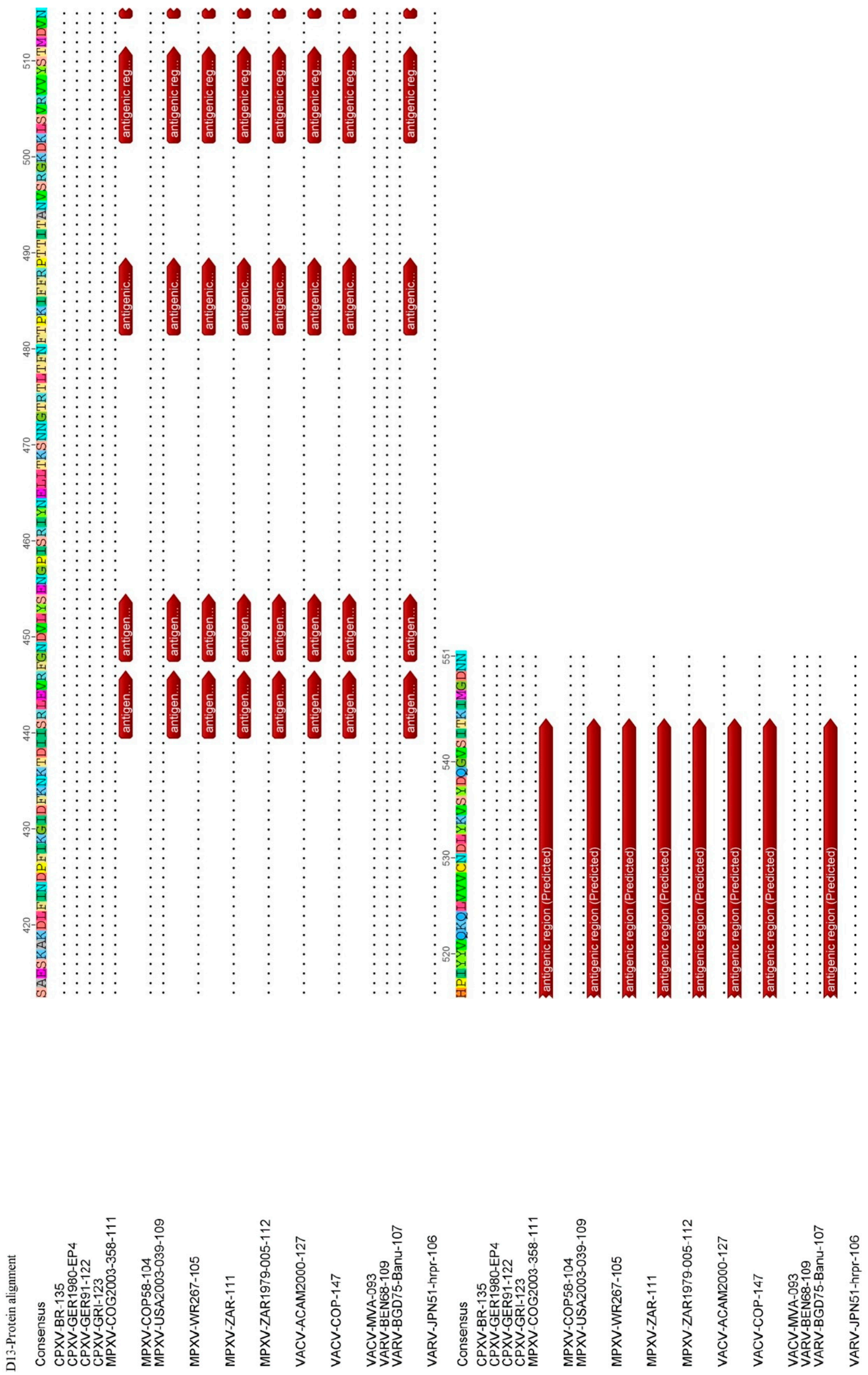

Figure S16. Sequence alignment for protein D13. Page 3 of 3.

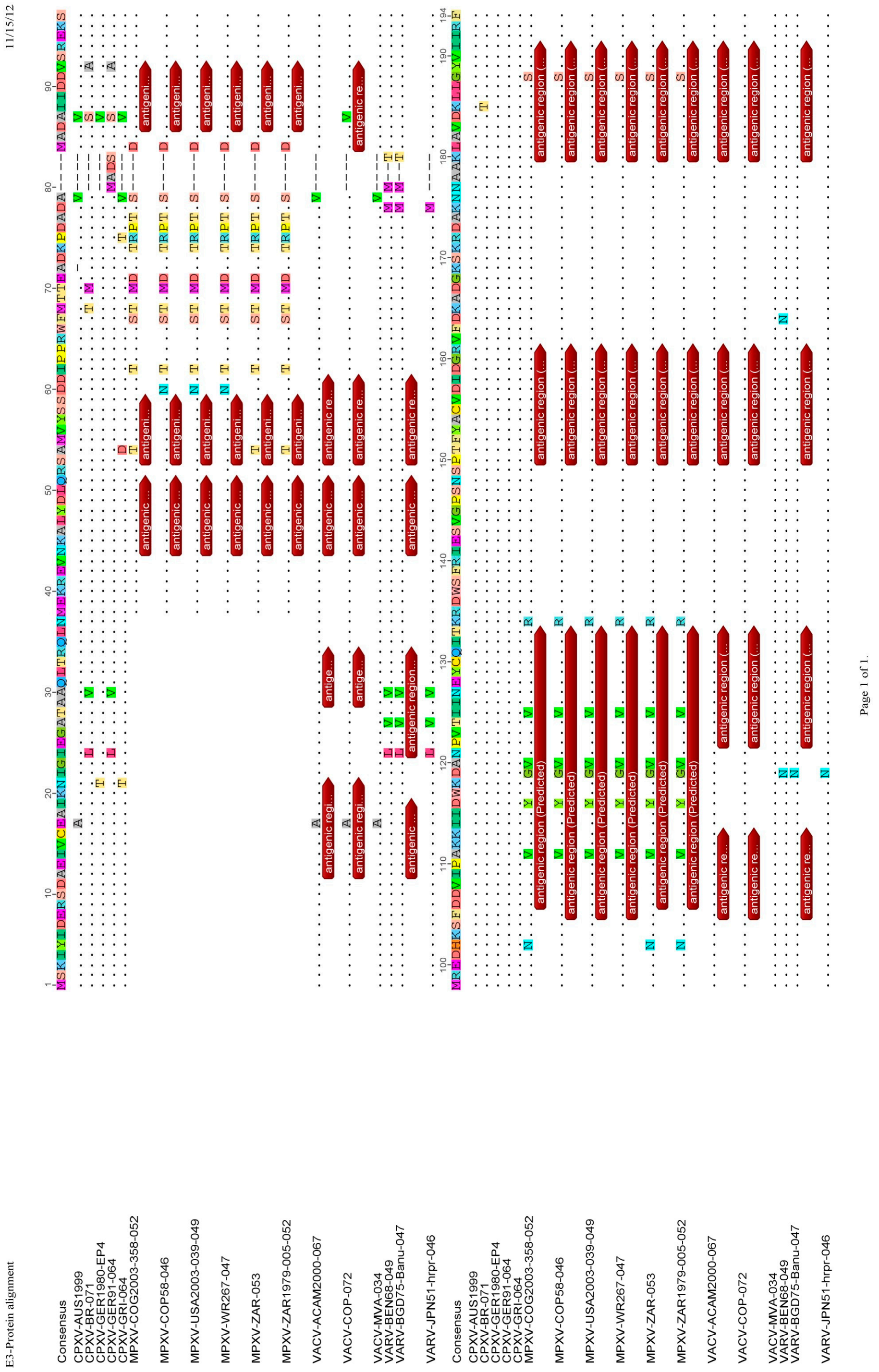

Figure S17. Sequence alignment for protein E3.

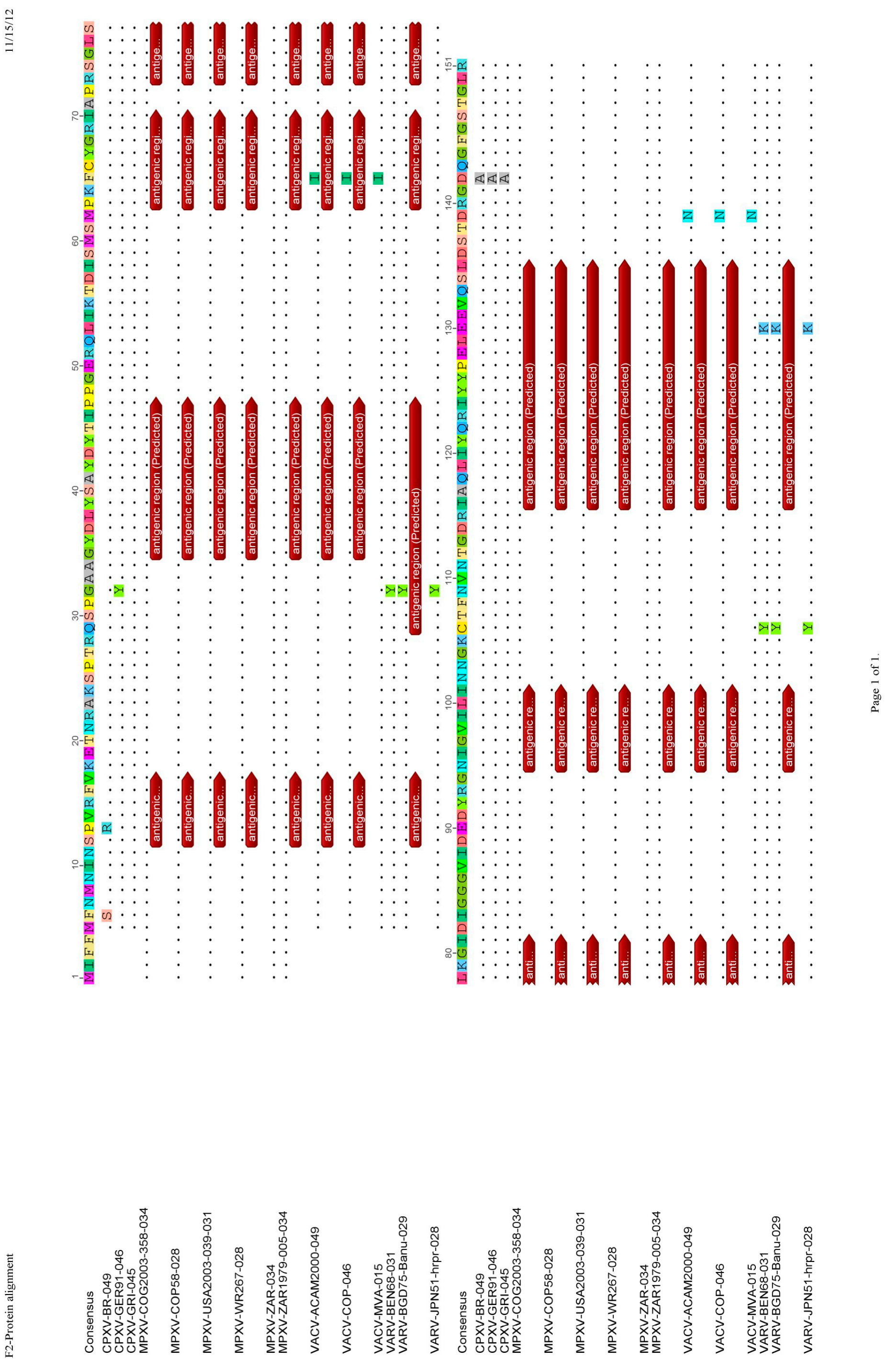

Figure S18. Sequence alignment for protein F2.

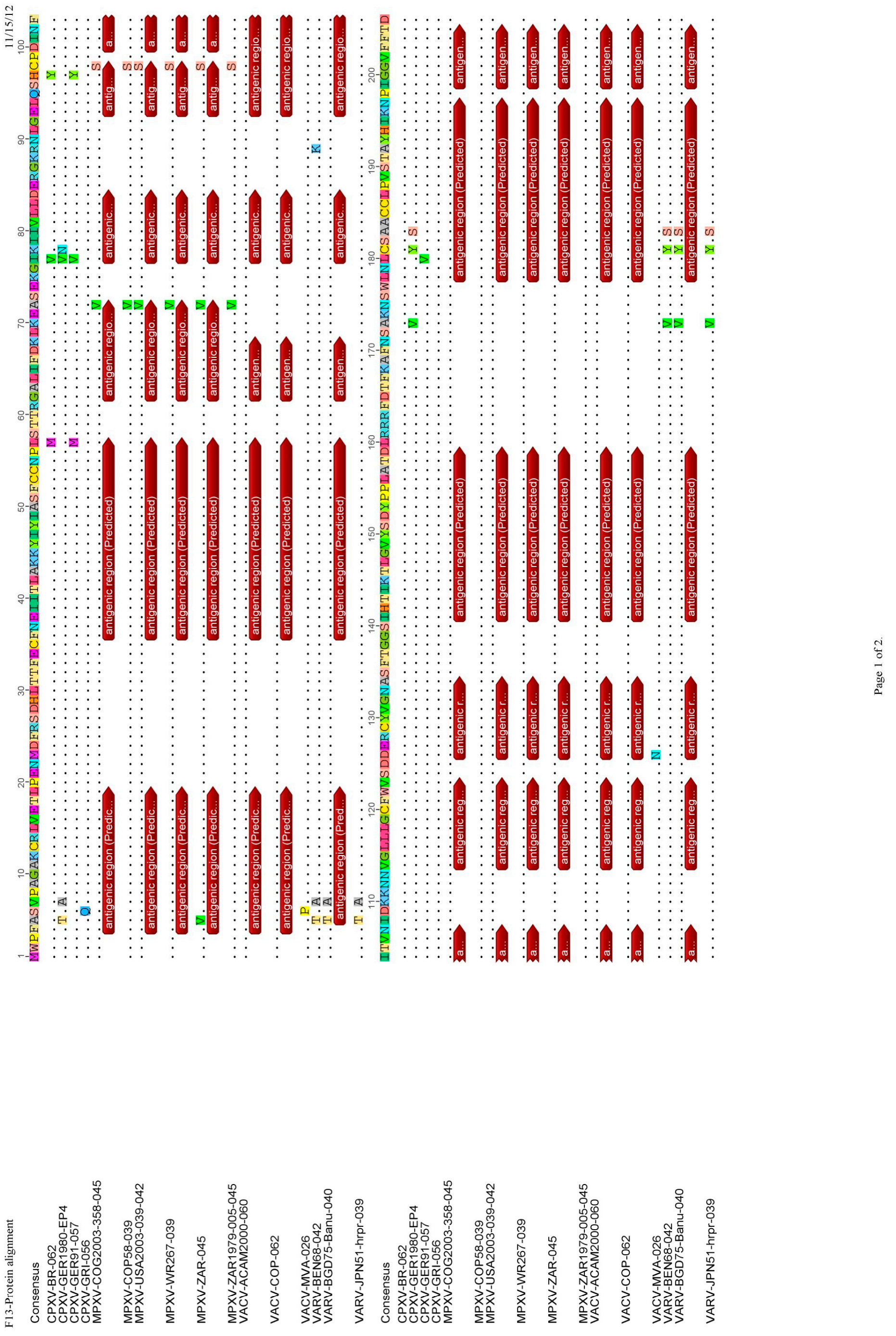

Figure S19. Sequence alignment for protein F13. Page 1 of 2.

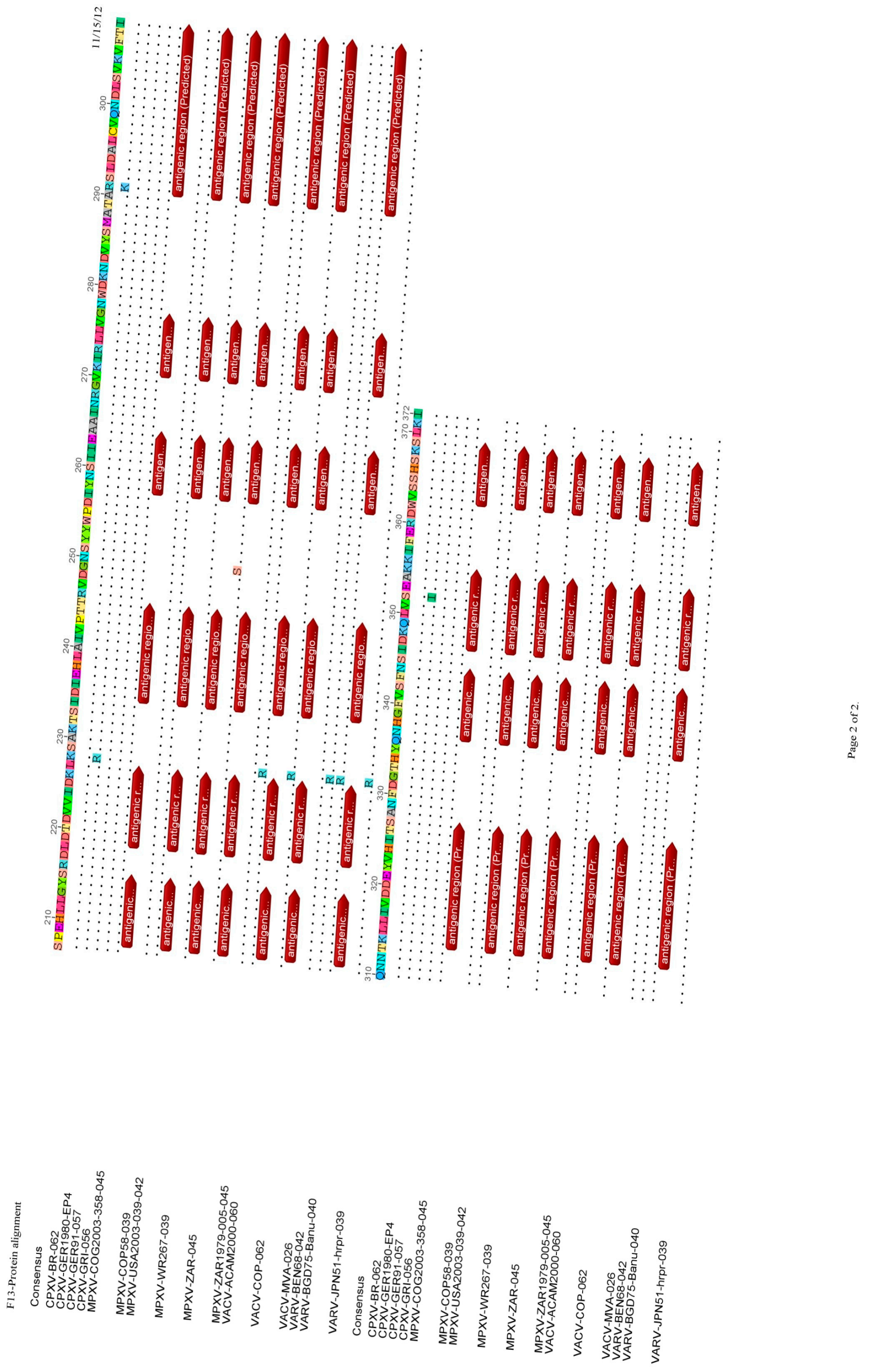

Figure S19. Sequence alignment for protein F13. Page 2 of 2.

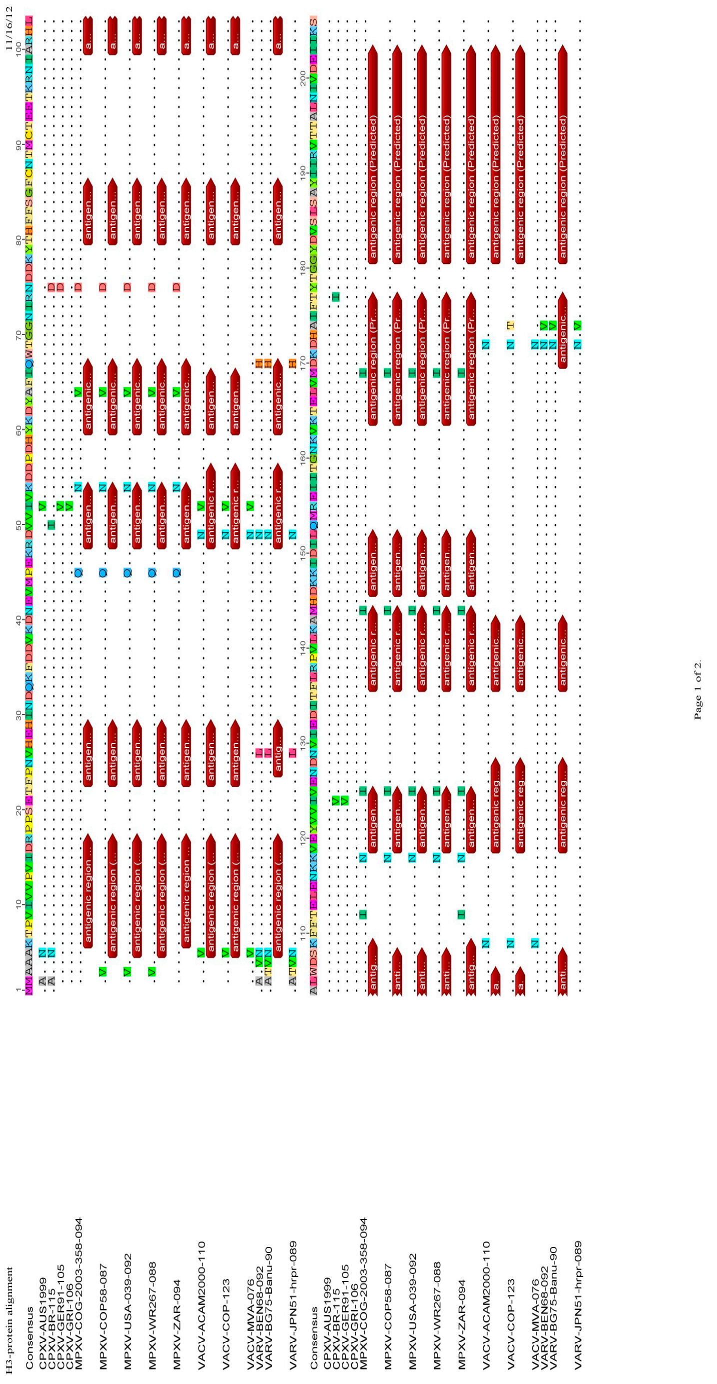

Figure S20. Sequence alignment for protein H3. Page 1 of 2.

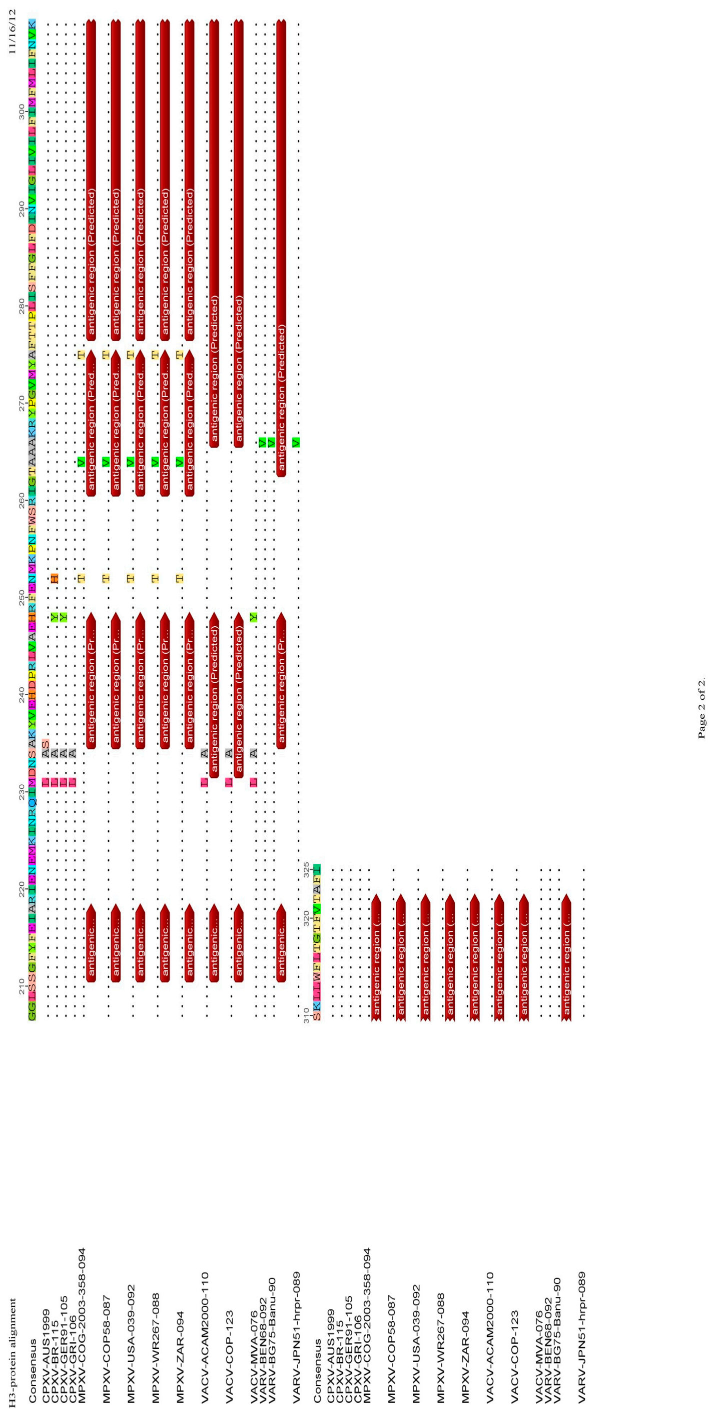

Figure S20. Sequence alignment for protein H3. Page 2 of 2.

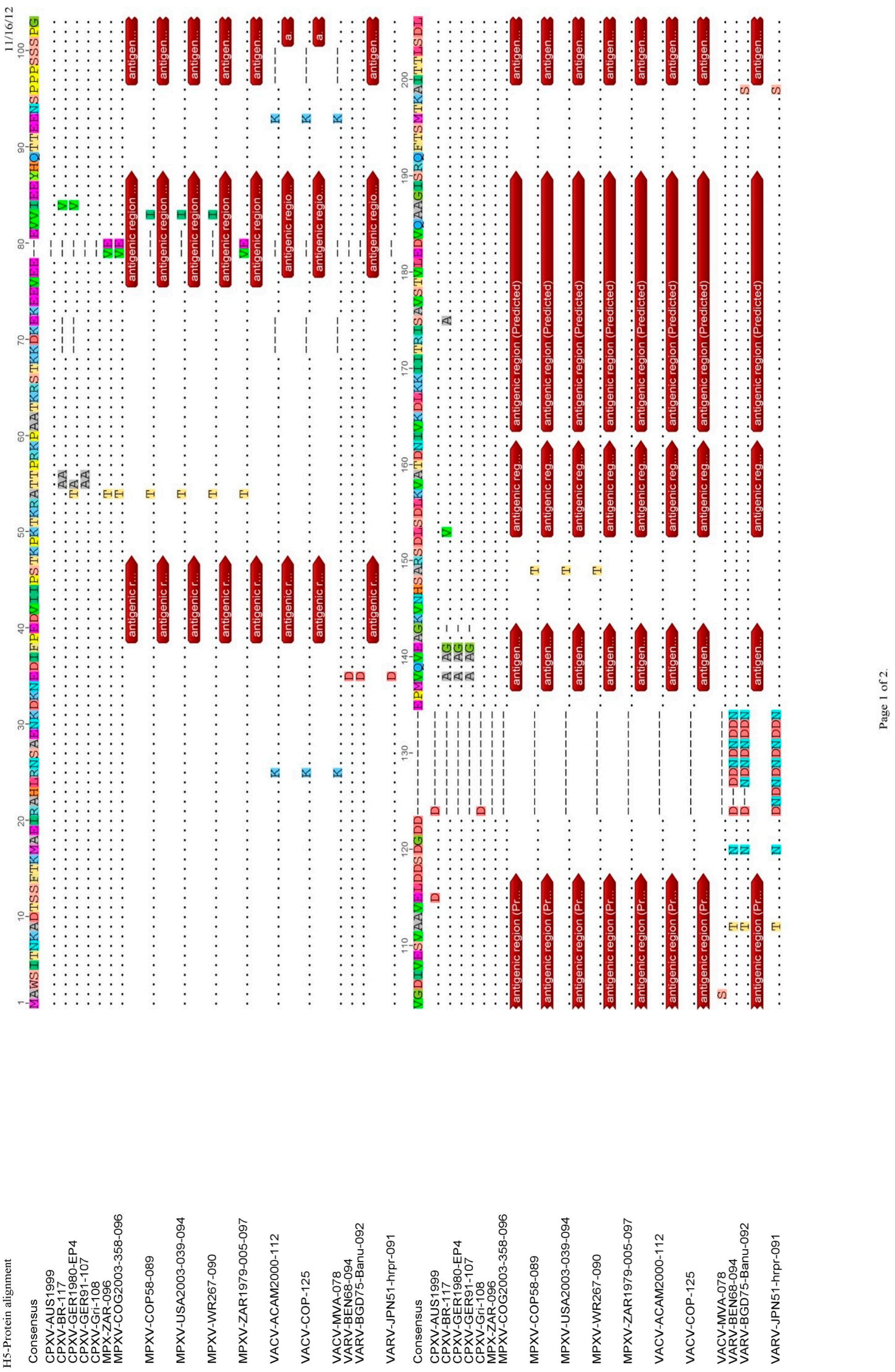

Figure S21. Sequence alignment for protein H5. Page 1 of 2.

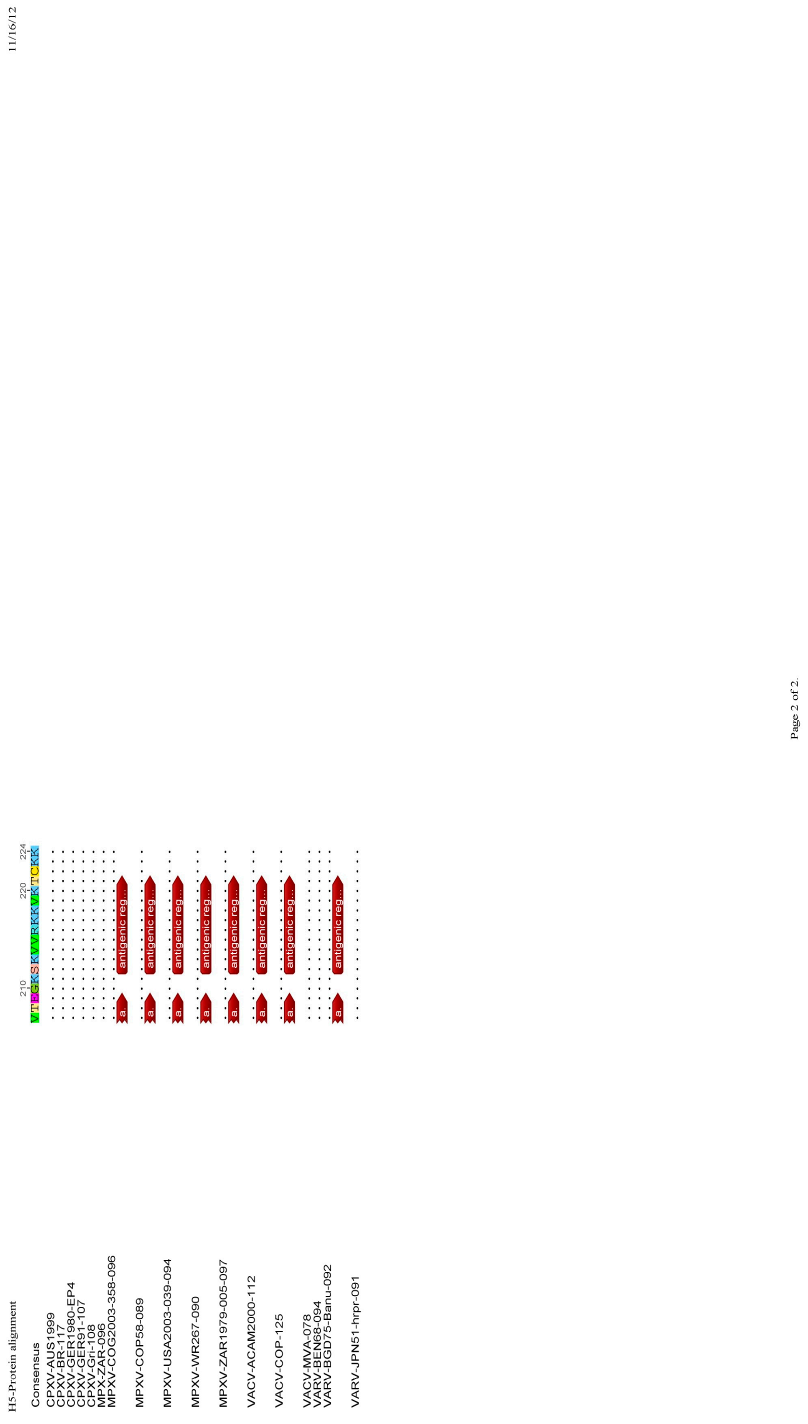

Figure S21. Sequence alignment for protein H5. Page 2 of 2.

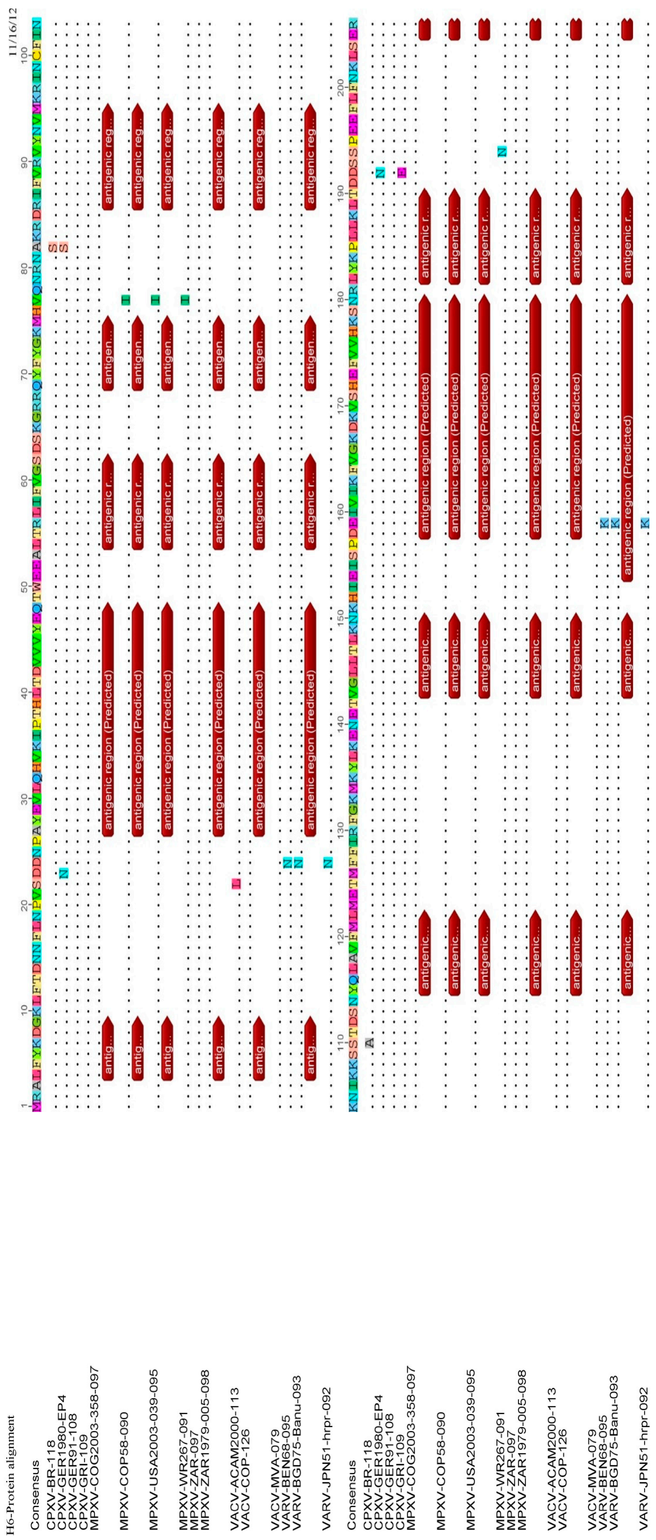

Figure S22. Sequence alignment for protein H6. Page 1 of 2.

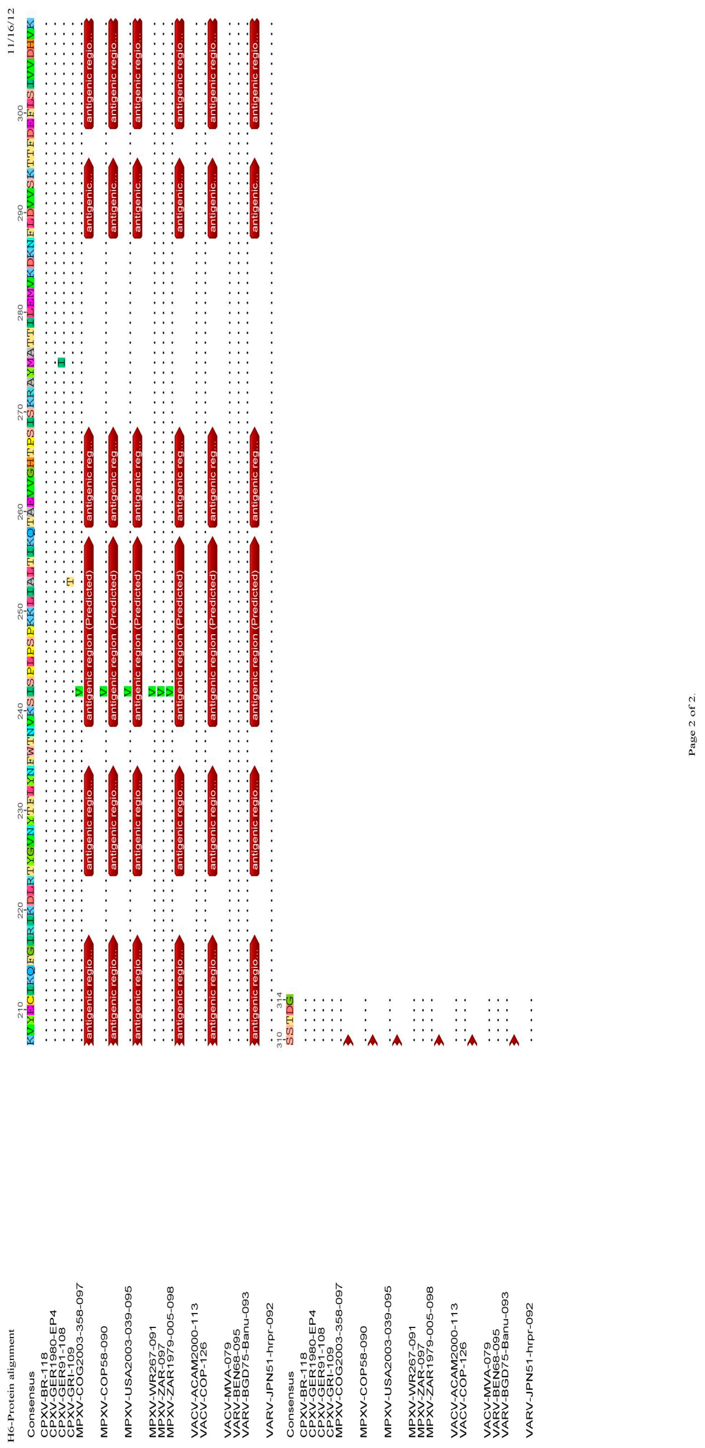

Figure S22. Sequence alignment for protein H6. Page 2 of 2.

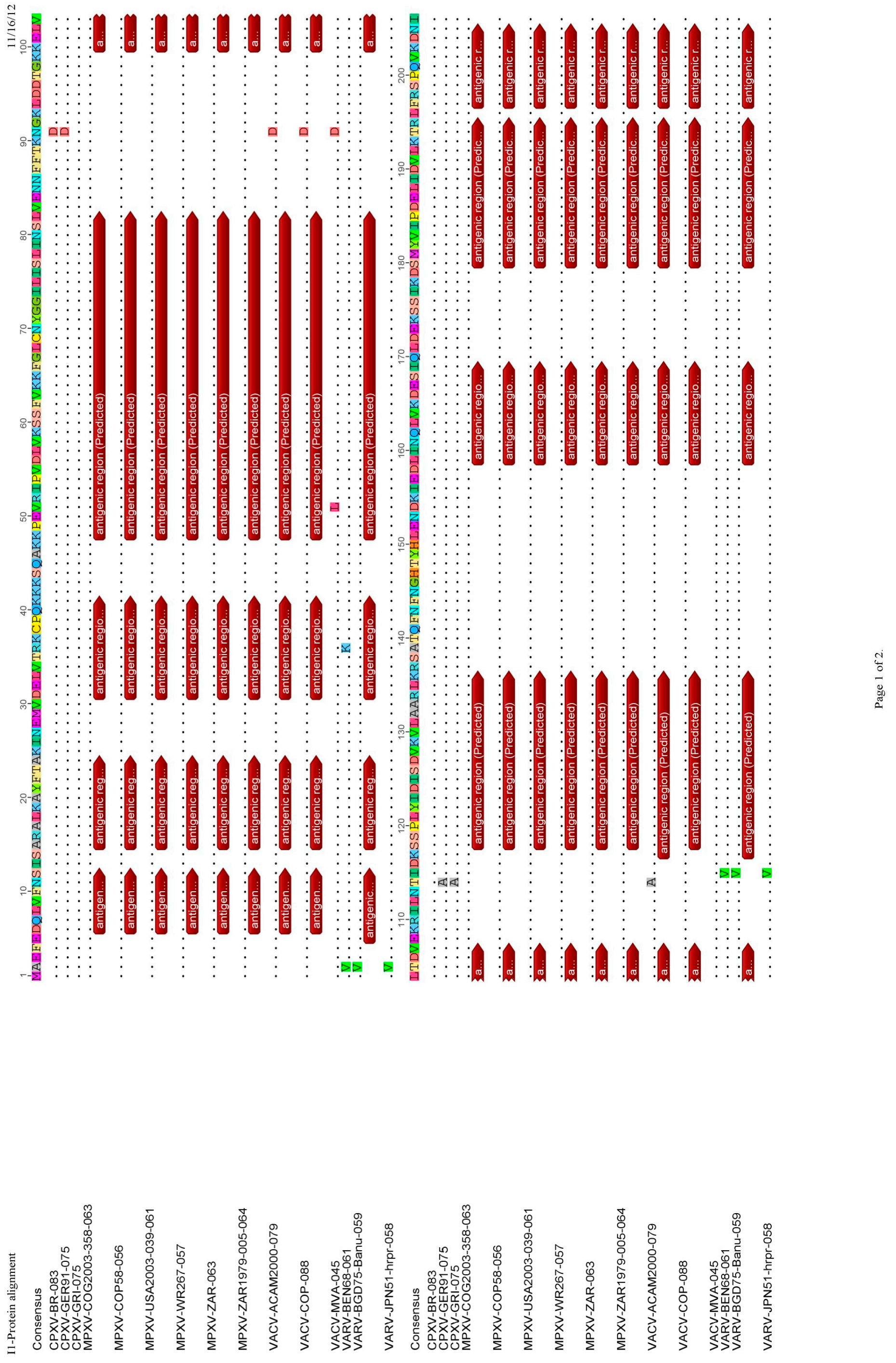

Figure S23. Sequence alignment for protein I1. Page 1 of 2.

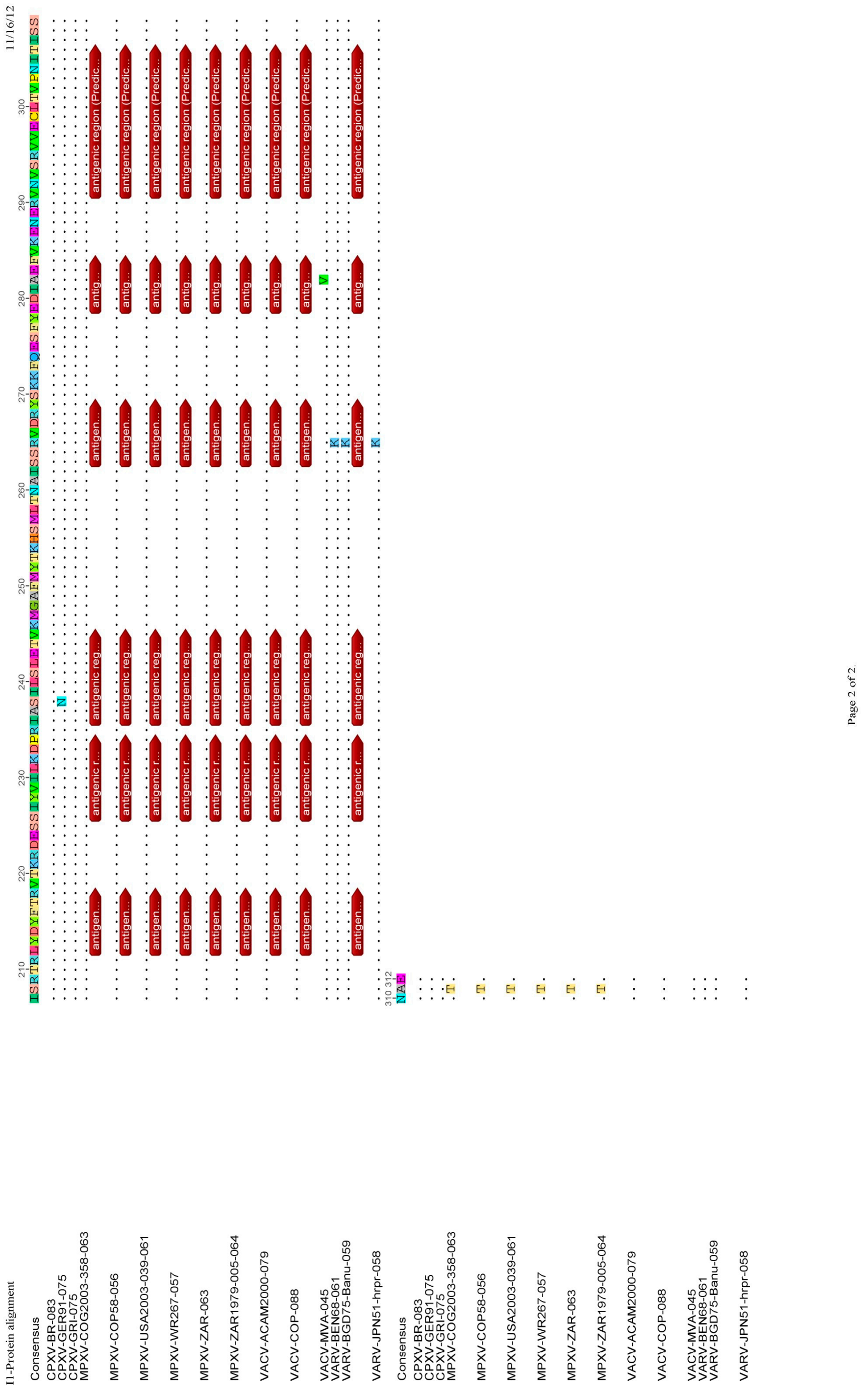

Figure S23. Sequence alignment for protein I1. Page 2 of 2.

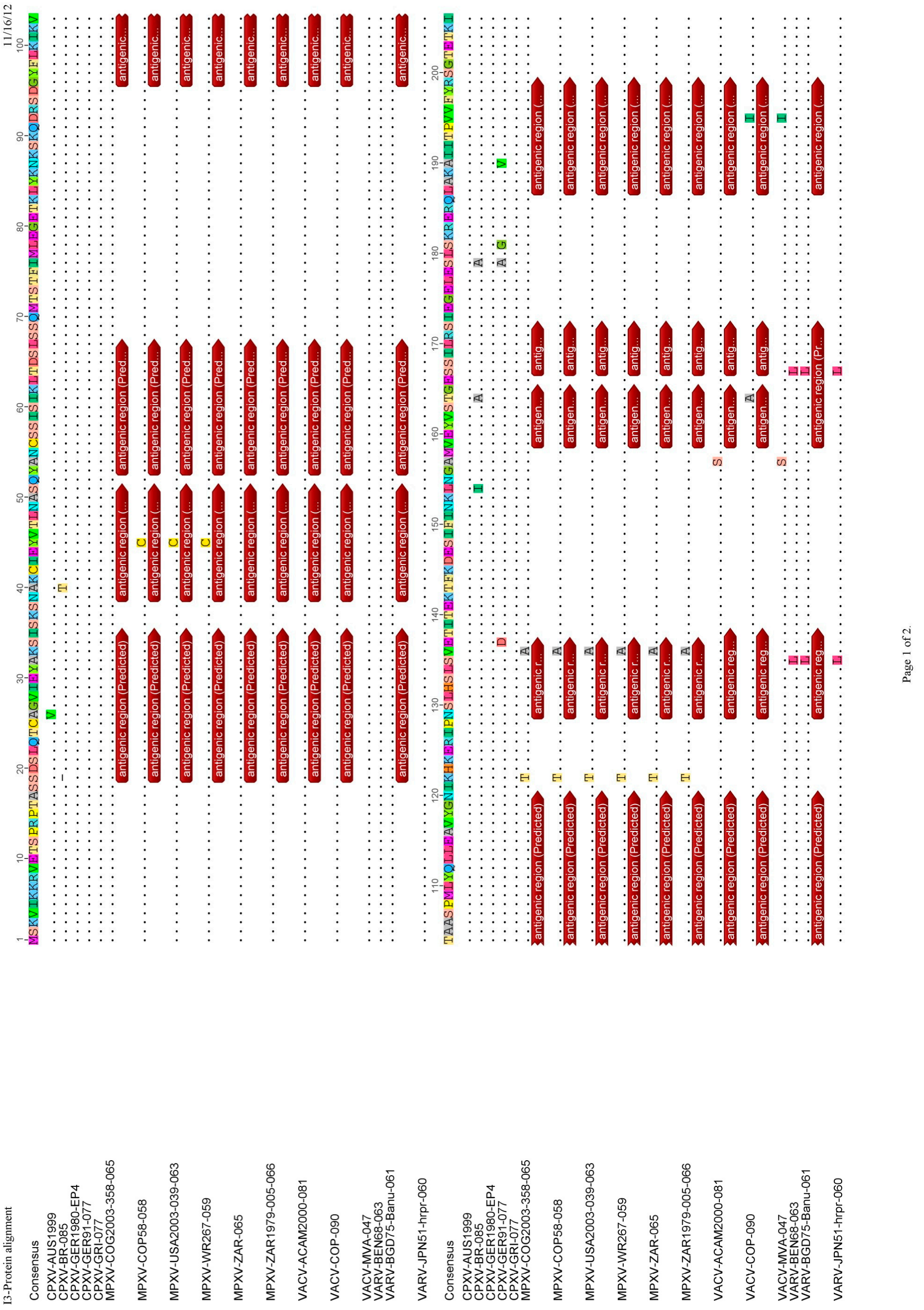

Figure S24. Sequence alignment for protein I3. Page 1 of 2.

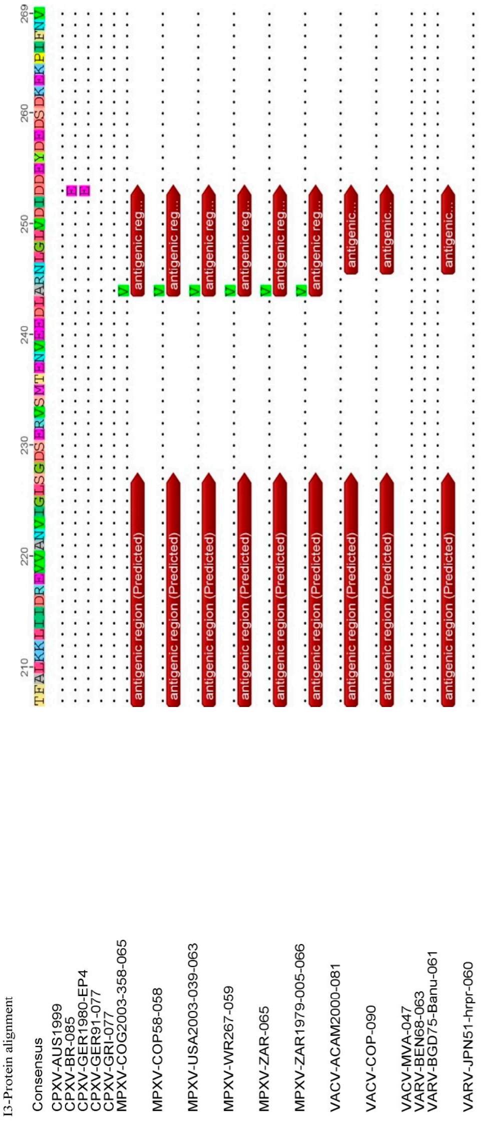

Page 2 of 2.

Figure S24. Sequence alignment for protein I3. Page 2 of 2.

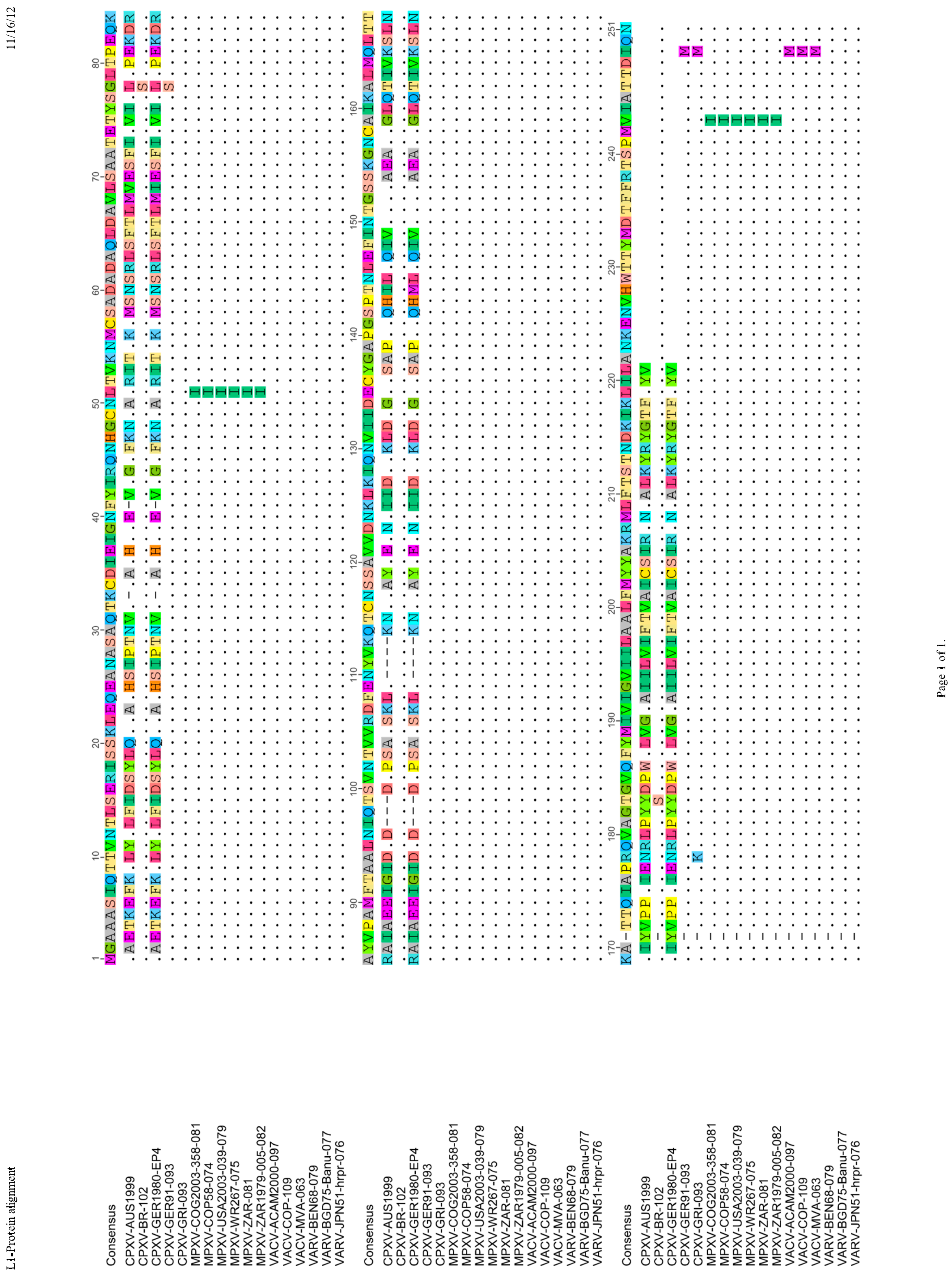

Figure S25. Sequence alignment for protein L1.

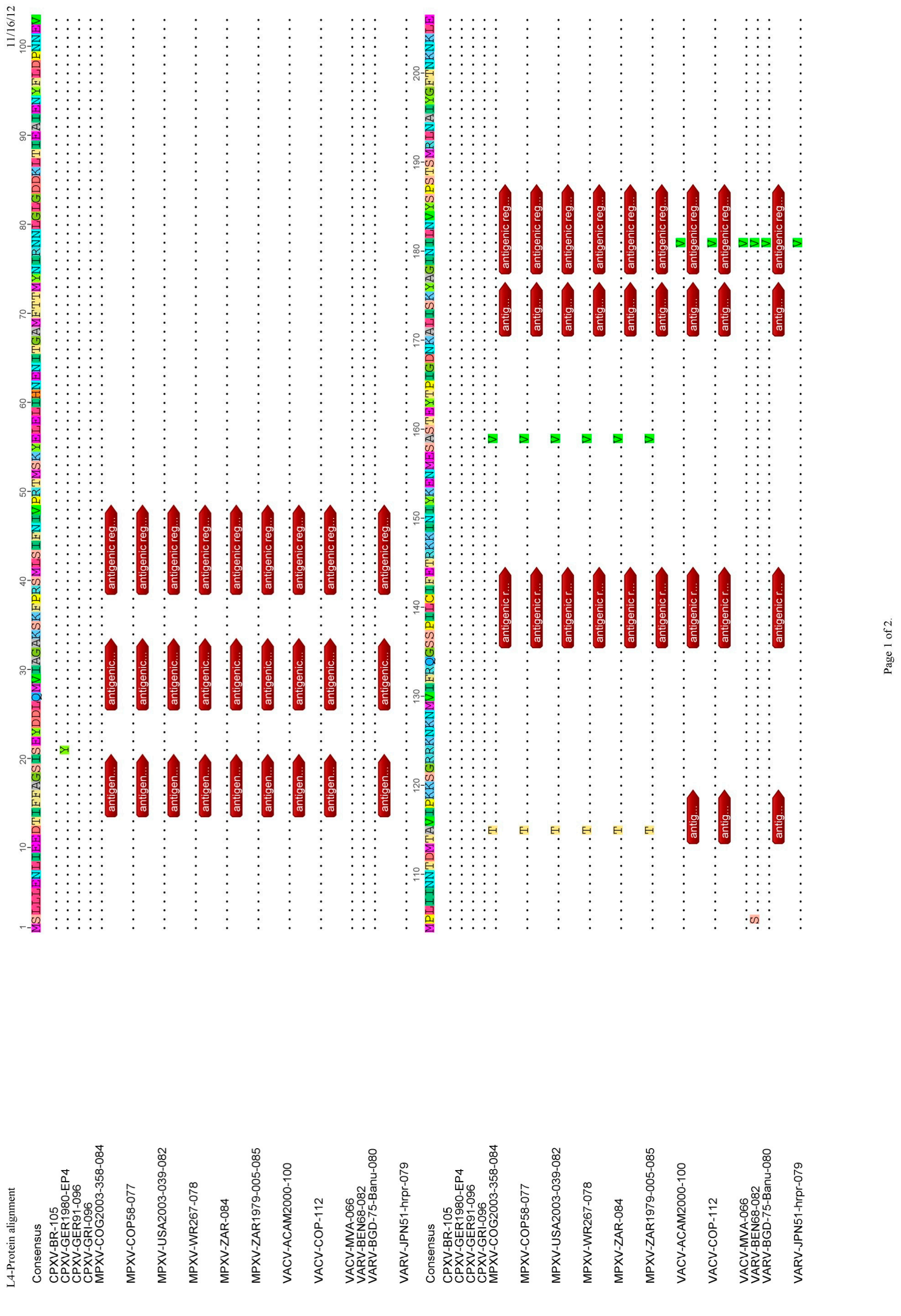

Figure S26. Sequence alignment for protein L4. Page 1 of 2.

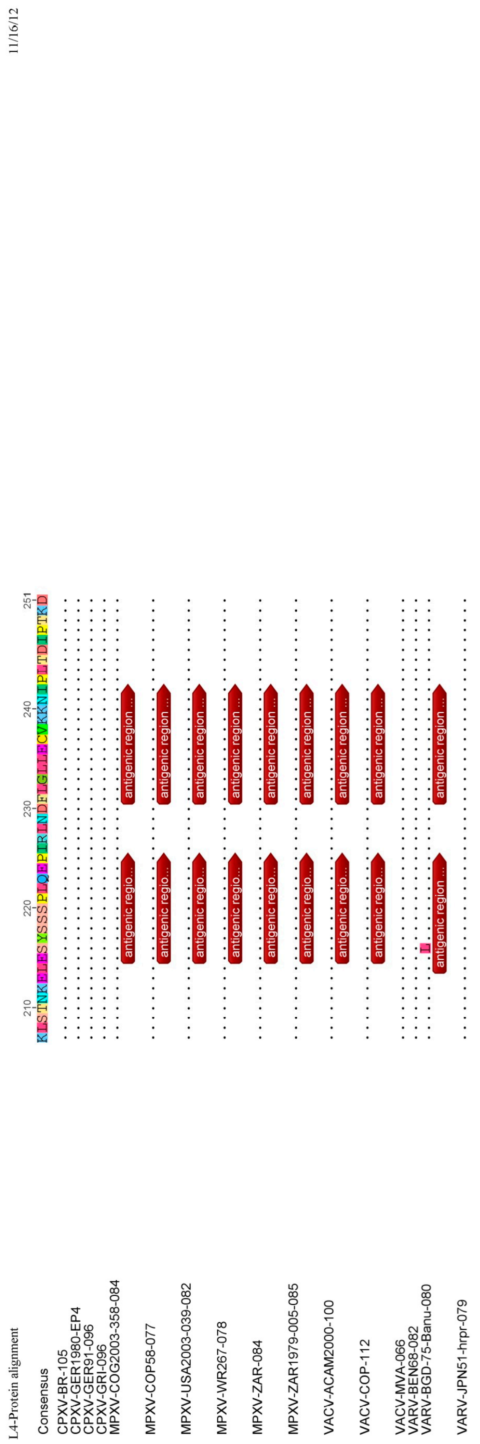

Figure S26. Sequence alignment for protein L4. Page 2 of 2.

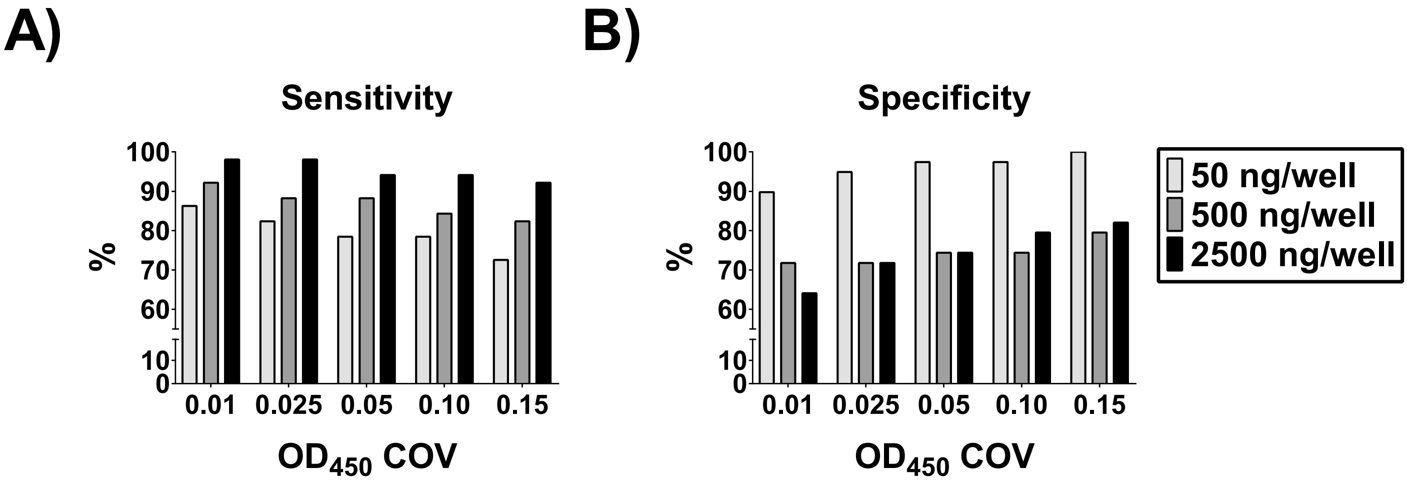

Figure S27. Optimization of peptide concentration for MPXV peptide-based ELISA. The concentration of the peptide combination A33 + E3 + A56 was varied per each well and then ELISA conducted to screen all of the DRC samples as well as the VACV vaccine study samples. Sensitivity **(A)** and specificity **(B)** were calculated for each of the peptide concentrations at different OD450 COVs.

References:

38. Townsend, M.B.; Keckler, M.S.; Patel, N.; Davies, D.H.; Felgner, P.; Damon, I.K.; Karem, K.L. Humoral immunity to smallpox vaccines and monkeypox virus challenge: Proteomic assessment and clinical correlations. *J. Virol.* **2013**, *87*, 900–911. https://doi.org/10.1128/jvi.02089-12.
